# 4-Phenylbutyric acid activates an NF-κB-Egr-1 axis to control myoblast proliferation and ECM gene expression

**DOI:** 10.1016/j.isci.2026.117620

**Published:** 2026-09-24

**Authors:** Kana Tominaga, Naoomi Tominaga

**Affiliations:** 1 Division of Neuropsychiatry, Department of Neuroscience, Yamaguchi University Graduate School of Medicine, 1-1-1 Minami-kogushi, Ube, Yamaguchi 755-8505, Japan; 2 Division of Clinical Laboratory Sciences, Department of Nursing and Laboratory Science, Yamaguchi University Graduate School of Medicine, 1-1-1 Minami-kogushi, Ube, Yamaguchi 755-8505, Japan; 3 Division of Advanced Cell Therapy, Yamaguchi University Research Institute for Cell Design Medical Science (RICeD), 1-1-1 Minami-kogushi, Ube, Yamaguchi 755-8505, Japan

**Keywords:** 4-phenylbutyric acid, HDAC5, myoblast proliferation, NF-κB/Egr-1 signaling, extracellular matrix remodeling

## Abstract

Myogenic progression is tightly controlled by epigenetic mechanisms. Here, we demonstrate that the histone deacetylase inhibitor 4-phenylbutyric acid (4-PBA) selectively promotes myoblast proliferation without inducing myotube differentiation. Mechanistically, 4-PBA increases histone H3 acetylation at lysines 18 and 27 through HDAC5 suppression, resulting in activation of NF-κB p65. HDAC5 overexpression attenuates 4-PBA-induced histone H3 acetylation, NF-κB/early growth response 1 (Egr-1) activation, and myoblast proliferation, supporting a causal role for HDAC5 suppression. Chromatin immunoprecipitation identifies Egr-1 as a direct transcriptional target of NF-κB p65. Transcriptomic analyses reveal that Egr-1 regulates extracellular matrix- and myogenesis-associated gene programs, including nonfibrillar collagen genes. Together, these findings define an epigenetic mechanism by which 4-PBA modulates myoblast proliferation through HDAC5-dependent histone acetylation and NF-κB p65-Egr-1-driven transcriptional programs, providing insight into how epigenetic therapeutics influence skeletal muscle cell behavior.

## Highlights

• 4-PBA enhances murine myoblast proliferation independently of differentiation induction
• 4-PBA enhances H3K18 and H3K27 acetylation through downregulation of HDAC5
• NF-κB p65 activates Egr-1 by directly binding to the Egr-1 promoter region
• 4-PBA activates the NF-κB p65-Egr-1 axis and induces extracellular matrix-related genes

## Graphical abstract

**Figure:**
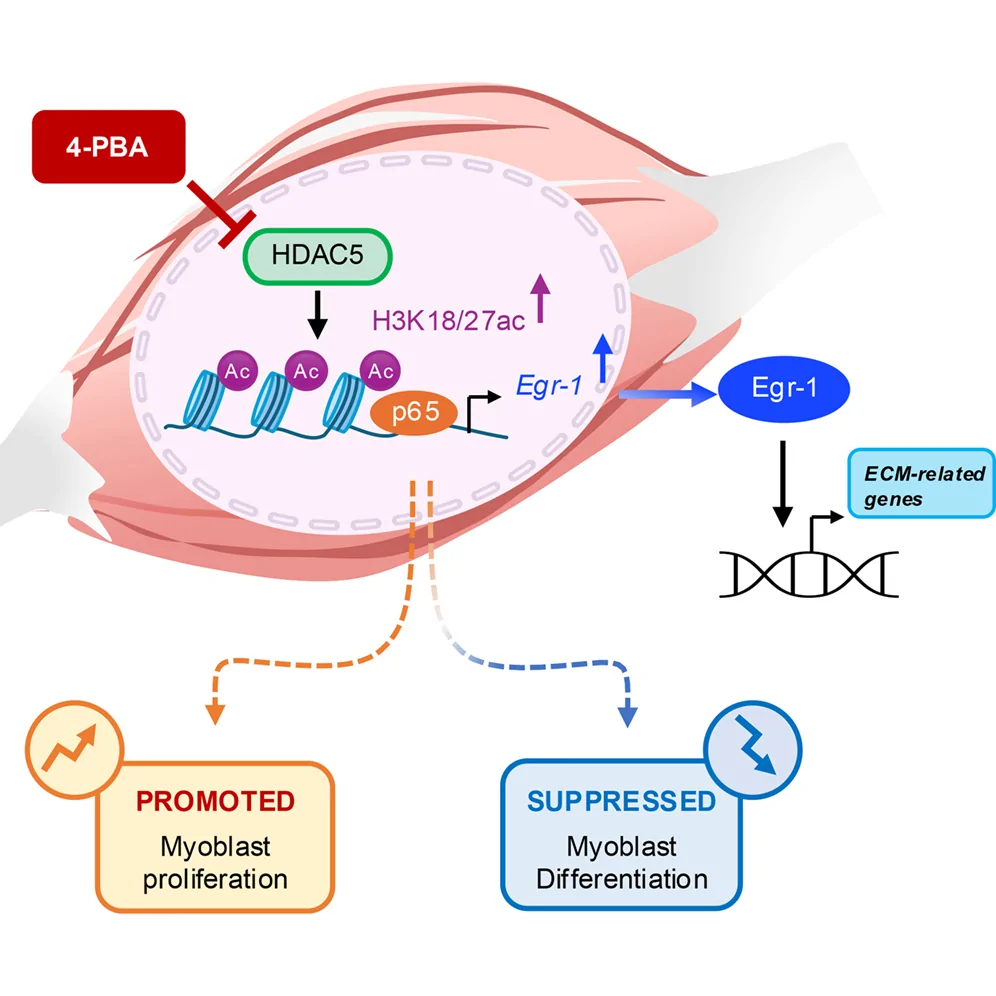

## Teaser

Molecular mechanism of gene regulation; Epigenetics; Integrative aspects of cell biology; Functional aspects of cell biology

## Introduction

Myoblasts are proliferative precursor cells derived from muscle satellite cells^1^^,^^2^ Satellite cells are skeletal muscle stem cells (MuSCs) that reside in a quiescent state beneath the basal lamina of muscle fibers.^3^^,^^4^ In response to muscle injury or growth signals, satellite cells become activated and give rise to myoblasts, which proliferate and subsequently differentiate to form new muscle fibers (myotubes/myofibers).^5^^,^^6^ This process is essential for skeletal muscle growth, regeneration, and repair and represents a promising therapeutic target for muscle-related diseases.^7^^,^^8^

Histone acetylation plays a central role in the proliferation and differentiation of myoblasts.^9^ During differentiation, histone acetyltransferases (HATs) are recruited to the promoters and enhancers of muscle-specific genes, leading to increased acetylation of histone marks such as H3K9ac and H3K27ac.^10^^,^^11^^,^^12^ This epigenetic remodeling activates key myogenic genes, including *Myog*, *myosin heavy chain (Myh)*, and muscle creatine kinase.^10^^,^^13^ The myogenic transcription factor MyoD recruits HATs such as p300/CBP and PCAF to muscle gene loci, enhancing chromatin accessibility and reinforcing muscle-specific transcription.^14^^,^^15^ Concurrently, histone deacetylases (HDACs) are displaced from muscle gene promoters but remain associated with cell cycle-related genes to ensure permanent withdrawal from proliferation.^16^^,^^17^ Pharmacological inhibition of HDAC increases histone acetylation and can accelerate myoblast differentiation, underscoring the importance of balanced acetylation in myogenesis.^18^

4-PBA is currently used clinically to treat patients with urea cycle disorders under the names Buphenyl (sodium phenylbutyrate) and Ravicti (glycerol phenylbutyrate).^19^ Its primary mechanism of action involves binding toxic ammonia to provide an alternative pathway for its elimination.^19^^,^^20^ In addition, 4-PBA functions as a chemical chaperone that reduces endoplasmic reticulum (ER) stress and prevents protein aggregation.^21^^,^^22^^,^^23^ 4-PBA has attracted interest for disorders involving protein misfolding, such as cystic fibrosis with the ΔF508 *CFTR* mutation and vascular Ehlers-Danlos syndrome with *COL3A1* mutations.^24^^,^^25^^,^^26^ Previous studies have shown that 4-PBA treatment restores the membrane localization of pathogenic mutant dysferlin (Dysf) in cultured myotubes and myofibers obtained from dysferlinopathy model mice, suggesting that 4-PBA rescues dysferlin from aggregation or degradation in the ER.^27^ 4-PBA is also recognized as an HDAC inhibitor, adding an epigenetic mechanism to its therapeutic effects. During cardiomyogenesis, 4-PBA promotes early cardiac differentiation via increased histone acetylation but inhibits late-stage differentiation by maintaining the expression of pluripotency genes such as *Oct4*, *Nanog*, and *Sox2*, demonstrating that HDAC regulation exerts context-dependent effects.^28^ In the case of cancer, 4-PBA inhibits tumor cell proliferation by increasing histone acetylation, inducing epithelial-mesenchymal transition, and promoting acetyl-H3-mediated transcription.^29^^,^^30^^,^^31^ Hence, these effects suggest that 4-PBA may alleviate ER stress caused by misfolded proteins and enhance protein homeostasis. In contrast, despite the clinical response to 4-PBA in diseases caused by misfolded proteins, little is known about its role as an HDAC inhibitor of myogenesis.

Our study demonstrated that 4-PBA regulates myoblast differentiation through epigenetic and transcriptional mechanisms. In C2C12 myoblasts, chromatin immunoprecipitation (ChIP) assays revealed that NF-κB p65 directly binds to the *Egr-1* promoter, identifying Egr-1 as a transcriptional target of the NF-κB signaling pathway. During myogenesis, 4-PBA treatment markedly increased histone H3 acetylation, especially at Lys18 and Lys27, and downregulated HDAC5 expression, resulting in increased Egr-1 and p65 expression. Furthermore, 4-PBA increased H3K18ac and H3K27ac enrichment at the *Egr-1* promoter, whereas HDAC5 overexpression attenuated 4-PBA-induced histone H3 acetylation, NF-κB/Egr-1 activation, and myoblast proliferation. Moreover, transcriptome analysis revealed that Egr-1 regulated extracellular matrix (ECM)-related genes, including multiple collagen genes. This study revealed that 4-PBA acts as an HDAC inhibitor that contributes to myogenic homeostasis by regulating the NF-κB-Egr-1 axis.

## Results

### 4-PBA induces cell proliferation in murine myoblasts

To confirm the effects of 4-PBA on myoblast differentiation, we first examined morphological changes in C2C12 myoblasts following 4-PBA treatment. C2C12 myoblasts were cultured under differentiation conditions and treated with 4-PBA for four days (Figure S1A). Myotube formation was then assessed by measuring the myofusion index and the protein levels of myosin heavy chain 1 (Myh1), a major contractile protein, using immunofluorescence analysis (Figure 1A). We observed a significant decrease in the fusion index and reduced Myh1 expression in 4-PBA-treated cells compared with control cells (Figure 1B). We next examined the protein expression of myogenin (Myog), a myogenic transcription factor, and Dysf, a marker of mature muscle fibers, under 4-PBA-treated conditions (Figure 1C). The results showed that 4-PBA significantly suppressed the induction of Myog at all stages (day 1–4) and Dysf at later stages (day 3–4) of myotube differentiation (Figure 1D). To further exclude the possibility that 4-PBA induces delayed differentiation, we extended the observation period in C2C12 cells up to day 7 under differentiation conditions. During this extended time course, 4-PBA did not promote late myogenic differentiation; rather, immunofluorescence (Figure S2A) and immunoblotting analyses (Figures S2B and S2C) showed sustained suppression of differentiation-associated markers and reduced myotube formation in 4-PBA-treated cultures compared with control cultures. In control cultures, the percentage of Myog-positive cells increased during differentiation, whereas this increase was reduced by 4-PBA treatment (Figures S2D and S2E). In contrast, PAX7-positive cells were maintained in 4-PBA-treated cultures, suggesting that 4-PBA suppresses terminal myogenic differentiation while preserving a less differentiated myogenic cell population (Figures S2F and S2G). Consistent with the C2C12 results, similar effects of 4-PBA were observed in non-immortalized primary myogenic cells isolated from mouse soleus muscle. In primary cells prepared from three independent mice, 4-PBA suppressed terminal myogenic differentiation, as shown by reduced differentiation marker expression and impaired myotube formation, while maintaining myogenic lineage identity (Figures S3A–S3D). Moreover, PAX7-positive cells were maintained in 4-PBA-treated cultures, and this phenotype was reproduced in primary myogenic cells (Figures S3E and S3F). These findings indicate that the differentiation-suppressive effect of 4-PBA is not restricted to immortalized C2C12 cells.

**Figure 1:**
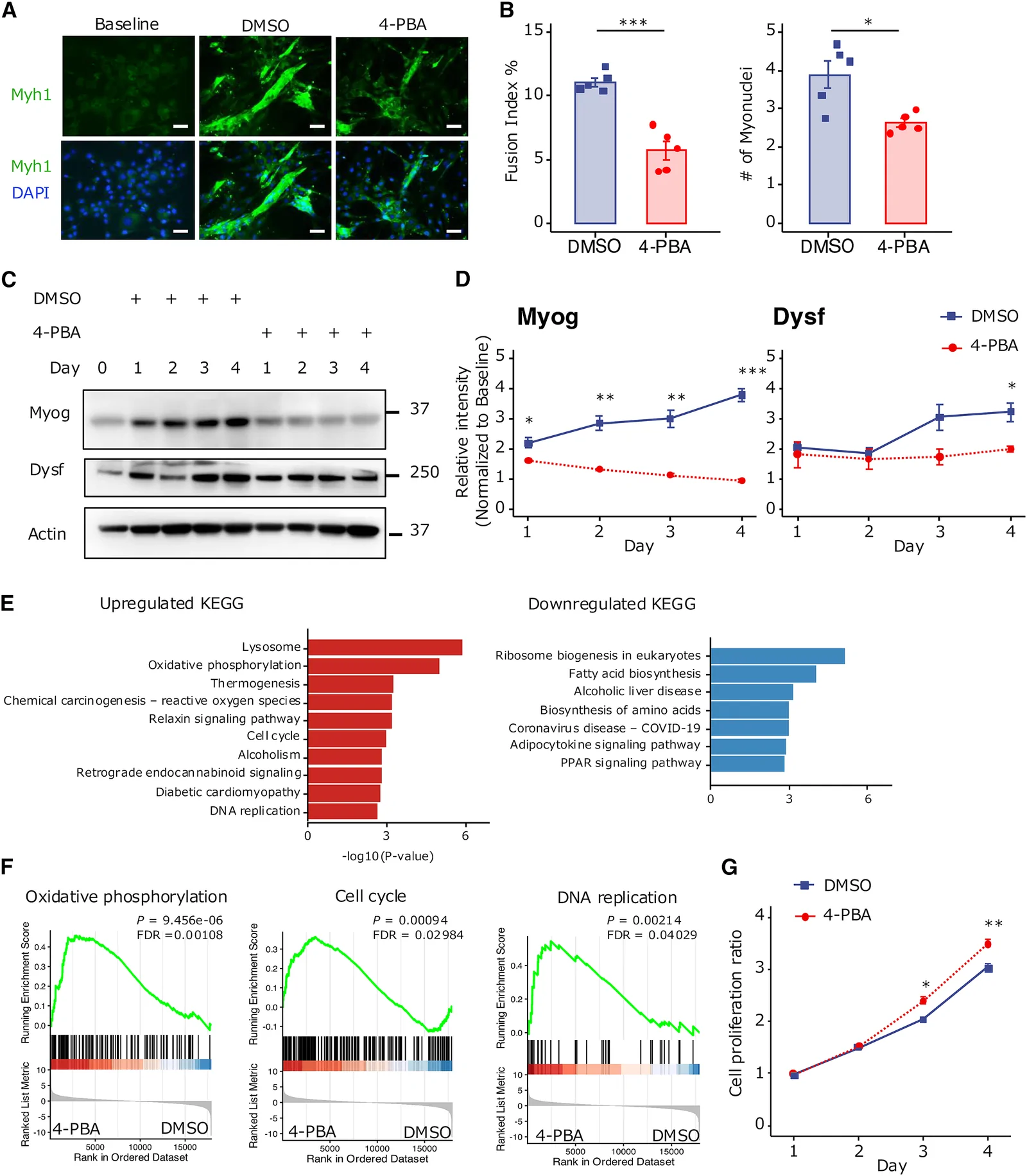
4-PBA promotes myoblast proliferation but impairs myogenic differentiation (A) Representative immunofluorescence images of C2C12 myotubes stained for Myh1 (green) and nuclei (blue). Scale bars, 400 μm. (B) (Left) Quantification of Myh1-positive myotube area per field after 4 days of differentiation (*n* = 5). (Right) the number of nuclei within multinucleated myotubes (*n* = 5). (C) Representative immunoblots showing myogenin (Myog) and dysferlin (Dysf) expression in C2C12 cells treated with 1 mM 4-PBA or DMSO for the indicated time points. (D) Quantification of Myog and Dysf protein levels shown in (C), normalized to β-actin (Actin). Data are representative of three independent experiments. (E) KEGG pathway enrichment analysis of differentially expressed genes in C2C12 myotubes at day 4 after differentiation, comparing treatment with 1 mM 4-PBA and DMSO. (F) Gene set enrichment analysis (GSEA) plots for the indicated KEGG pathways in 4-PBA- or DMSO-treated cells. FDR, false discovery rate. (G) Proliferation of C2C12 myoblasts treated with 1 mM 4-PBA or DMSO. Data in (B), (D), and (G) are presented as mean ± SEM. and analyzed using an unpaired two-tailed Student’s *t* test. ∗*p* < 0.05, ∗∗*p* < 0.01, ∗∗∗*p* < 0.001.

To identify the biological pathways affected by 4-PBA treatment in myoblasts, we analyzed RNA sequencing (RNA-seq) data (GSE315556) from C2C12 myoblasts treated with 4-PBA under differentiation conditions for four days. An RNA-seq analysis of myoblast differentiation from day 0 to day 4 indicated that there was no difference in the expression of myoblast differentiation-related genes between the DMSO- and 4-PBA-treated cells (Figures S1B–S1D). These results suggest that 4-PBA suppresses myogenic differentiation primarily through post-transcriptional, translational, or protein stability-related mechanisms rather than by regulating myogenic gene expression at the mRNA level. Kyoto Encyclopedia of Genes and Genomes (KEGG) pathway analysis revealed that genes upregulated by 4-PBA treatment were primarily associated with cell proliferation-related pathways (Figure 1E; Data S2). Three representative pathways were visualized to facilitate interpretation (Figure 1F). To validate the RNA-seq results indicating enhanced proliferation, we examined cell proliferation in C2C12 myoblasts treated with 4-PBA or DMSO as a control. Compared with DMSO-treated cells, C2C12 myoblasts treated with 4-PBA showed significantly increased proliferation after four days (Figure 1G), suggesting that compared with myoblast differentiation, 4-PBA promotes cell proliferation. Importantly, this proliferation-promoting effect was also confirmed in primary myogenic cells (Figure S3G), supporting the physiological relevance of the 4-PBA response beyond the C2C12 cell line.

### 4-PBA induces Egr-1 expression during myogenesis

Because 4-PBA has multifaceted functions beyond HDAC inhibition, including chemical chaperone activity and ER stress modulation, we first evaluated ER stress-related signaling in myogenic cells. Under 4-PBA-treated conditions, the levels of glucose-regulated protein 78 (GRP78), phosphorylated eukaryotic translation initiation factor 2 alpha (p-eIF2α), phosphorylated inositol-requiring enzyme 1 (p-IRE1), and C/EBP homologous protein (CHOP) were reduced, whereas activating transcription factor 4 (ATF4) and activating transcription factor 6 (ATF6) were not significantly changed (Figures S4A and S4B). Treatment with tauroursodeoxycholic acid (TUDCA), an ER stress-reducing chemical chaperone, did not promote cell proliferation at any concentration tested (Figures S4C and S4D). Tunicamycin, a potent inducer of ER stress, significantly increased ER stress markers and markedly reduced cell proliferation, indicating that ER stress induction negatively affects myoblast proliferation. These results indicate that 4-PBA attenuates selected ER stress-related pathways under our experimental conditions; however, ER stress attenuation alone is not sufficient to reproduce the proliferation-promoting effect of 4-PBA.

We next investigated that 4-PBA acts as an HDAC inhibitor, thereby increasing histone acetylation and consequently promoting gene expression. To identify genes upregulated by 4-PBA treatment during myogenic differentiation, we compared the gene expression profiles of 4-PBA-treated C2C12 myoblasts with those of DMSO-treated or undifferentiated (baseline) cells via RNA-seq (GSE315556, Data S3). Principal component analysis (PCA) revealed that compared with the baseline treatment, the 4-PBA and DMSO treatments caused significant changes in the state, after which the 4-PBA-treated C2C12 myotubes formed a distinct cluster that was clearly separated from the DMSO treatment (Figure 2A). These results indicated a strong and consistent global transcriptional change caused by 4-PBA treatment during myogenesis. Transcriptomic analysis revealed that only two genes, *Egr-1* and *Fos*, were differentially expressed genes (DEGs) based on the criteria of | log2-fold change | > 1.2 and *p* < 0.01 (Figures 2B and 2C). The mRNA expression levels of both genes were greater in 4-PBA-treated C2C12 myotubes than in DMSO-treated cells or baseline controls (Figure 2D). At the protein level, Egr-1 expression markedly increased in 4-PBA-treated C2C12 cells compared with that in DMSO-treated and control cells, particularly during the early stages of differentiation (Figures 2E and 2F). Notably, Egr-1 induction was detected 2–3 days after 4-PBA treatment, suggesting that this response is unlikely to represent only an acute, immediate stress response. Instead, Egr-1 may reflect sustained transcriptional remodeling associated with the 4-PBA-induced proliferative and less differentiated myogenic state. In contrast, Fos protein expression did not significantly differ between 4-PBA- and DMSO-treated C2C12 cells. These findings indicated that Egr-1 is a primary and functionally relevant target of 4-PBA in the transformation of C2C12 myoblasts to myotubes. Furthermore, the induction of Egr-1 by 4-PBA was also observed in primary myogenic cells, indicating that the Egr-1 response is conserved between C2C12 cells and non-immortalized primary myogenic cells (Figures S5A and S5B). These findings indicated that Egr-1 is a primary and functionally relevant target of 4-PBA in the transformation of murine myoblasts to myotubes.

**Figure 2:**
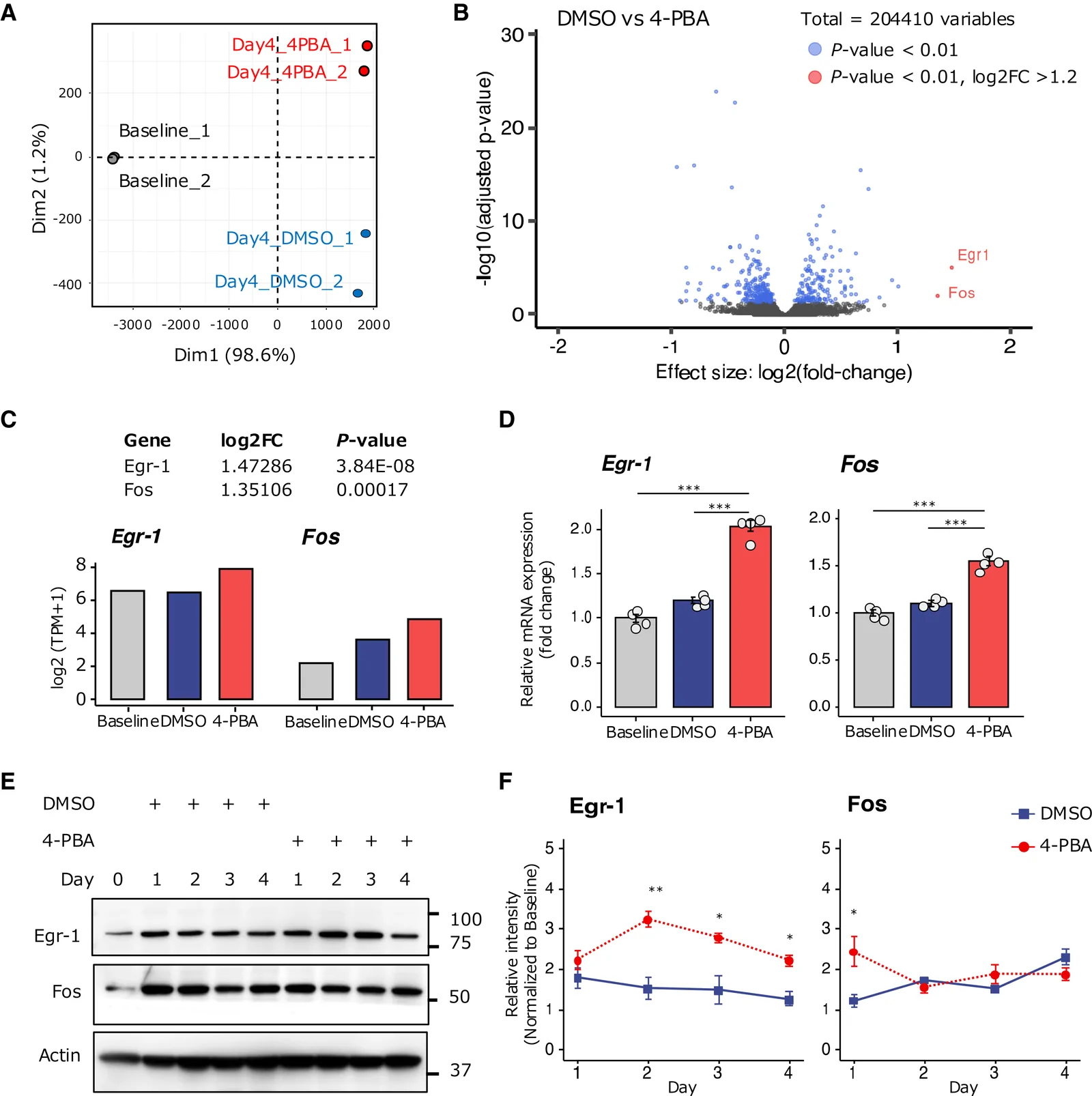
4-PBA induces Egr-1 expression during myoblast differentiation (A) Principal component analysis (PCA) showing sample clustering based on differentially expressed genes in C2C12 cells treated with 1 mM 4-PBA or DMSO. Baseline indicates cells prior to differentiation induction. (B) Volcano plot of differentially expressed genes identified by RNA-seq analysis comparing 4-PBA-treated and DMSO-treated C2C12 cells following myotube differentiation after 4 days. Significantly upregulated genes are shown in red. FC, fold change; *p*-adj, adjusted *p* value. (C) RNA-seq-based expression levels of *Egr-1* and *Fos* mRNA in C2C12 cells treated with 1 mM 4-PBA or DMSO, shown as transcripts per million (TPM). (D) Quantitative PCR (qPCR) validation of *Egr-1* and *Fos* mRNA expression in C2C12 cells treated with 1 mM 4-PBA or DMSO. Data represent four independent experiments. (E) Representative immunoblots showing Egr-1 and Fos protein expression in C2C12 cells treated with 1 mM 4-PBA or DMSO for the indicated time points. (F) Quantification of protein levels shown in (E), normalized to β-actin (Actin). Data represent three independent experiments. Data in (D) and (F) are presented as mean ± SEM. and analyzed using one-way ANOVA with Tukey’s post hoc test (D) and unpaired two-tailed Student’s *t* test (F). Each dot represents one independent experiment. ∗*p* < 0.05, ∗∗*p* < 0.01, ∗∗∗*p* < 0.001.

### 4-PBA modulates the NF-κB p65-dependent expression of murine myoblasts

To identify the signaling pathway that regulates Egr-1 expression, we focused on the Erk pathway and the NF-κB p65 signaling pathway. Some studies have shown that 4-PBA inhibits the activation of both pathways in cells, primarily through its ability to reduce ER stress. We treated C2C12 or primary murine myoblasts with 1 mM 4-PBA or DMSO for four days during myotube differentiation and then evaluated the phosphorylation of Erk or NF-κB p65. Erk1/2 phosphorylation remained unchanged following 4-PBA or DMSO treatment, whereas NF-κB p65 expression and phosphorylation increased in 4-PBA-treated murine myoblasts from the early stages of differentiation (Figures 3A and 3B; Figures S5A and S5B). To confirm whether Egr-1 expression is regulated by the canonical NF-κB signaling pathway, we analyzed the protein levels of Egr-1 in 4-PBA-treated C2C12 myoblasts treated with (−)-dehydroxymethylepoxyquinomicin (DHMEQ), an NF-κB p65 inhibitor, for 24 h. Immunoblotting revealed that Egr-1 protein levels increased in response to 4-PBA treatment but decreased in response to the addition of DHMEQ (Figures 3C and 3D). NF-κB p65 may act as a transcription factor that regulates Egr-1.

**Figure 3:**
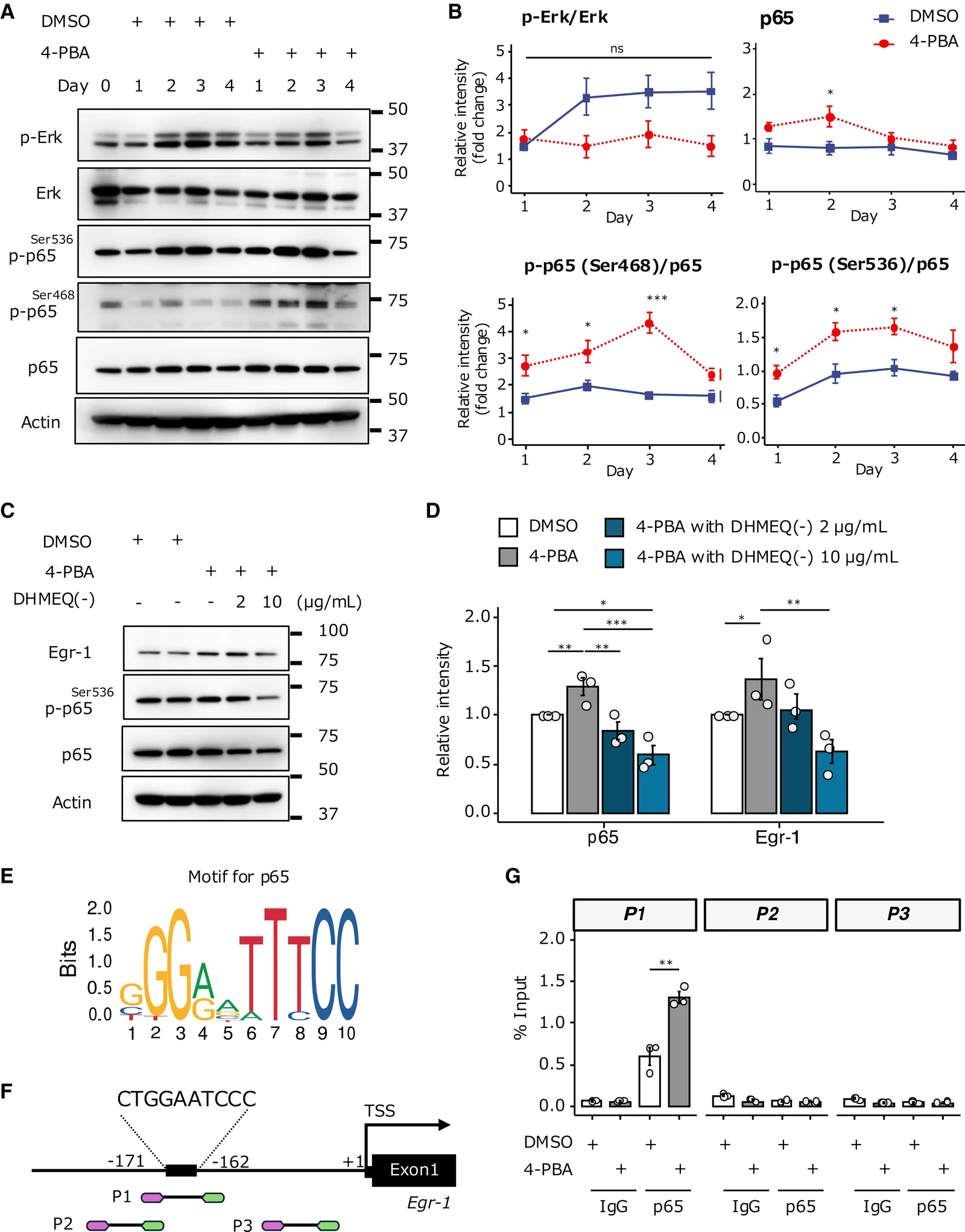
4-PBA regulates NF-κB-dependent Egr-1 expression in C2C12 cells (A) Representative immunoblots showing total Erk1/2, phosphorylated Erk1/2 (Thr202/Tyr204), total p65, and phosphorylated p65 (Ser468 and Ser536) in C2C12 cells treated with 1 mM 4-PBA or DMSO for the indicated time points. β-actin (Actin) was used as a loading control. (B) Quantification of phosphorylated Erk1/2 (Thr202/Tyr204), phosphorylated p65 (Ser468 and Ser536) and total p65 shown in (A). Band intensities were normalized to total Erk1/2, p65, or Actin, respectively. Data represent three independent experiments. (C) Representative immunoblots showing Egr-1 and p65 protein levels in C2C12 cells treated with 1 mM 4-PBA in the presence or absence of the NF-κB inhibitor DHMEQ. Actin was used as a loading control. (D) Quantification of Egr-1 and p65 protein levels shown in (C), normalized to Actin. Data represent three independent experiments. (E) NF-κB p65 consensus binding motif predicted using the JASPAR database. (F) Predicted p65 binding sites within the Egr-1 promoter region identified by JASPAR analysis. (G) Chromatin immunoprecipitation (ChIP) analysis showing enrichment of the *Egr-1* promoter regions (P1 and P2) in p65-or IgG-immunoprecipitated chromatin from 4-PBA treated C2C12 cells. Data in (B), (D), and (G) are presented as mean ± SEM. and analyzed using an unpaired two-tailed Student’s *t* test (B) and one-way ANOVA with Tukey’s post hoc test (D and G). Each dot represents one independent experiment. ∗*p* < 0.05, ∗∗*p* < 0.01, ∗∗∗*p* < 0.001; ns, not significant.

Putative NF-κB (p65) binding sites were identified based on the established κB consensus sequence (5′-GGGRNNYYCC-3′) in the *Homo sapiens* (GRCh38). However, within the EGR promoter region, a consensus NF-κB (p65) binding motif was identified in the human sequence, whereas no corresponding motif was detected in the mouse sequence. To identify NF-κB p65 binding sites that control Egr-1 transcription in the *Mus musculus* genome (GRCm39), we predicted putative NF-κB p65 binding sites within the −2000 bp promoter region of *Egr-1* using the JASPAR database (Figure 3E; Figure S6A). We designed two primer pairs encompassing the predicted motifs (P1) and two primer pairs flanking regions adjacent to the motifs (P2 and P3) to assess DNA enrichment (Figure 3F; Figure S6B). ChIP-qPCR analysis revealed that the P1 fragment was directly bound by NF-κB p65, whereas the other fragments (P2 and P3) were not enriched (Figure 3G). These results demonstrate that NF-κB p65 directly binds to the *Egr-1* promoter, specifically at the P1 region (CTGGAATCCC), indicating that *Egr-1* is a direct transcriptional target of NF-κB p65. Moreover, compared with DMSO-treated C2C12 myoblasts, 4-PBA-treated C2C12 myoblasts exhibited significantly increased binding of NF-κB p65 to the *Egr-1* promoter, suggesting that this treatment enhances NF-κB-mediated transcriptional regulation of Egr-1, potentially contributing to altered *Egr-1* gene expression under these conditions.

Because NF-κB pathway has been implicated in organelle remodeling and mitochondrial regulatory pathways,^32^ we next examined whether 4-PBA affects mitochondrial membrane potential. First, we accessed that the protein expression levels of dynamin-related protein1 (DRP1), optic atrophy 1 (OPA1), and peroxisome proliferator-activated receptor gamma coactivator 1-alpha (PGC1α) which regulated mitochondrial dynamics, biogenesis, and metabolic adaptation, during myogenic differentiation. The results showed that OPA1 and PGC1α protein levels increased compared with those in undifferentiated myoblasts, suggesting an activation of mitochondrial remodeling and biogenic programs during myotube formation (Figures S7A and S7B). However, this differentiation-associated induction of OPA1 and PGC1α proteins was suppressed by 4-PBA treatment in 4-PBA-treated myoblasts under differentiation conditions, suggesting that 4-PBA modulates the expression of proteins associated with mitochondrial dynamics and biogenesis.

We next analyzed whether mitochondrial membrane potential changed according to myogenic differentiation status in both myoblasts and myotubes using the JC-1 mitochondrial assay. JC-1 exists as green-fluorescent monomers under low mitochondrial membrane potential, whereas it forms red-fluorescent aggregates in mitochondria with high membrane potential. Therefore, JC-1 green monomer fluorescence reflects the low-potential or depolarized mitochondrial population, and the JC-1 red/green fluorescence ratio is used as an indicator of mitochondrial membrane potential. JC-1 green monomer fluorescence increased after culture or differentiation, and the JC-1 red/green ratio also changed during myogenic differentiation. However, no significant differences in JC-1 green fluorescence or the red/green ratio were observed between control and 4-PBA-treated cells in either myoblasts or myotubes (Figure S7C). Thus, although 4-PBA suppresses the expression of OPA1 and PGC1α, these changes were not accompanied by overt alterations in mitochondrial membrane potential or mitochondrial mass under our experimental conditions.

### 4-PBA induced the acetylation of histone H3 but not NF-κB p65

To determine whether 4-PBA treatment directly affects histone acetylation, we examined the acetylation status of histone H3 at residues H3K9, H3K14, H3K18, H3K27, and H3K56 during myogenesis by immunoblotting. In both C2C12 and primary murine myoblasts, Histone H3 acetylation markedly increased at the early stage of differentiation following inhibitor treatment, particularly at H3K18 and H3K27 (Figures 4A and 4B; Figures S8A and S8B). These results suggest that HDAC inhibition by 4-PBA alters the acetylation status of specific histone lysine residues.

**Figure 4:**
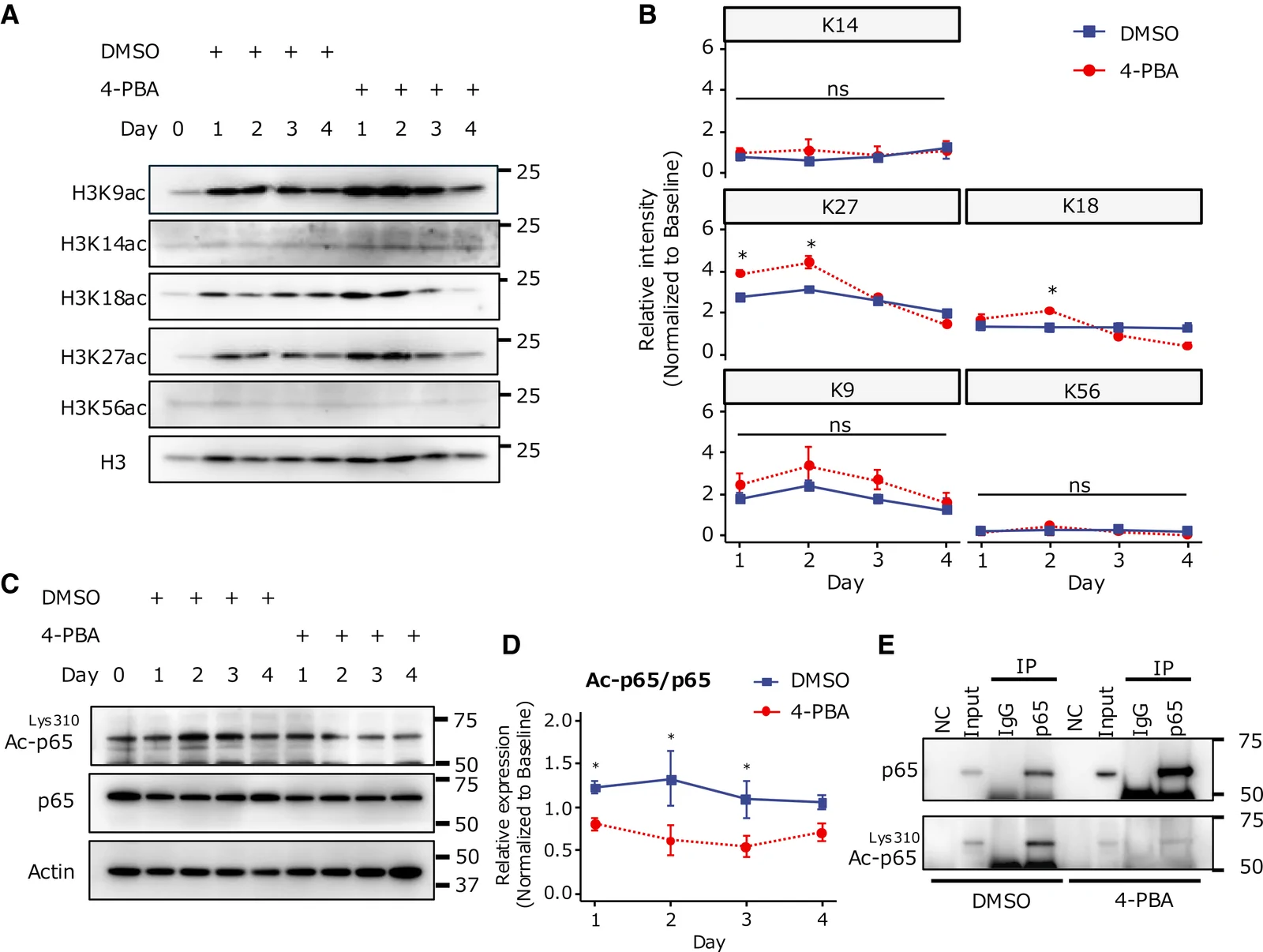
4-PBA induces histone H3K27/18 acetylation but does not promote p65 acetylation (A) Representative immunoblots showing acetylated histone H3 (H3K9/14/18/27/56ac) in C2C12 cells treated with 1 mM 4-PBA or DMSO for the indicated time points. Total histone H3 was used as a loading control. Same membrane was stripped and reprobed for different proteins. (B) Quantification of histone H3K27/18 acetylation shown in (A), normalized to total histone H3. Data represent three independent experiments. (C) Representative immunoblots showing total p65 and acetylated p65 (Lys310) in C2C12 cells treated with 1 mM 4-PBA or DMSO for the indicated time points. β-actin (Actin) was used as a loading control. (D) Quantification of acetylated p65 (Lys310) levels shown in (C), normalized to Actin. Data represent three independent experiments. (E) Immunoprecipitation of p65 from C2C12 cell lysates followed by immunoblot analysis with antibodies against total p65 or acetylated p65 (Lys310). Input lysates are shown as controls. Data in (B) and (D) are presented as mean ± SEM. and analyzed using an unpaired two-tailed Student’s *t* test (B and D). ∗*p* < 0.05, ∗∗*p* < 0.01, ∗∗∗*p* < 0.001; ns, not significant.

Other groups have reported that 4-PBA modulates NF-κB p65 and may regulate its acetylation.^33^ We therefore evaluated the acetylation of NF-κB p65 at Lys310 during C2C12 myoblast differentiation by immunoblotting. The acetylation of NF-κB p65 at Lys310 increased during myoblast differentiation; however, inhibitor treatment reduced p65 acetylation (Figures 4C and 4D). Consistently, coimmunoprecipitation using an antibody against p65 in C2C12 myotubes revealed that inhibitor treatment decreased NF-κB p65 acetylation (Figure 4E). These findings indicate that despite its strong effects on histone acetylation, 4-PBA does not increase, and instead reduces, NF-κB p65 acetylation in C2C12 cells.

### HDAC5 suppression contributes to 4-PBA-induced NF-κB/Egr-1 activation and myoblast proliferation

4-PBA suppresses Hdac5 protein expression, leading to the hyperacetylation of histone H3 in C2C12 myotubes.^34^ However, it is still unclear which class of HDACs is the primary target of 4-PBA. To investigate which classes of HDACs are predominantly downregulated by 4-PBA treatment during myogenesis, we first evaluated the gene expression of all the HDACs in C2C12 myotubes treated with 1 mM 4-PBA compared with those treated with DMSO. We observed significantly lower *Hdac5* gene expression in C2C12 myotubes than in DMSO control myotubes (Figure 5A; Data S4). Hdac5 expression increases during the early stages of differentiation, confirming that its expression is affected by the formation of immature myotubes (Figures 5B and 5C). Notably, primary myogenic cells showed a similar increase in Hdac5 expression during early differentiation (Figures S8A and S8C). In contrast, 4-PBA treatment reduced Hdac5 expression during myogenesis, which is consistent with the RNA-seq data. Together, these results suggest that Hdac5 is a key epigenetic regulator during early myogenesis and that 4-PBA treatment suppresses its expression, potentially leading to altered histone acetylation and dysregulated transcriptional programs during muscle differentiation.

**Figure 5:**
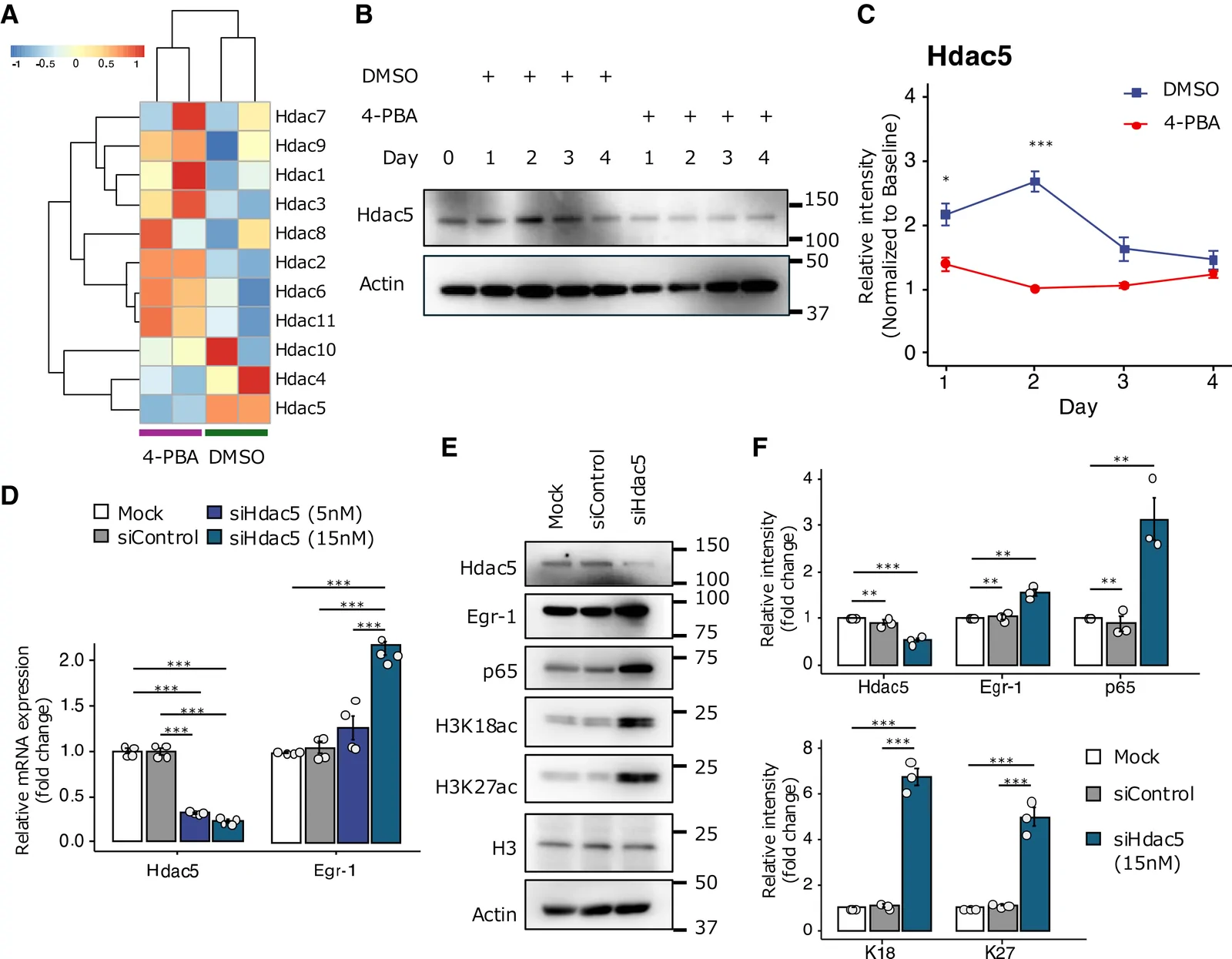
4-PBA regulates Egr-1 expression through suppression of HDAC5 in C2C12 cells (A) Heatmap showing RNA-seq-based expression profiles of histone deacetylase (HDAC) genes in C2C12 cells treated with 1 mM 4-PBA or DMSO. (B) Representative immunoblots showing HDAC5 protein levels in C2C12 cells treated with 1 mM 4-PBA or DMSO for the indicated time points. β-actin (Actin) was used as a loading control. (C) Quantification of HDAC5 protein levels shown in (B), normalized to Actin. Data represent three independent experiments. (D) Relative mRNA expression of *Hdac5* and *Egr-1* in C2C12 cells transfected with siHdac5 (5 or 15 nM), siControl, or mock control, as measured by qPCR. Expression levels were normalized to β-actin (Actin). (E) Representative immunoblots showing HDAC5, Egr-1, p65, H3K18ac, and H3K27ac protein levels in C2C12 cells treated with 1 mM 4-PBA or DMSO for the indicated time points. Actin or histone H3 was used as the loading control, as indicated. (F) Quantification of protein levels shown in (E). Band intensities were normalized to Actin for HDAC5, Egr-1, and p65, and to histone H3 for H3K18ac and H3K27ac. Data represent three independent experiments. Data in (C), (D), and (F) are presented as mean ± SEM. and analyzed using an unpaired two-tailed Student’s *t* test (C) and one-way ANOVA with Tukey’s post hoc test (D and F). Each dot represents one independent experiment. ∗*p* < 0.05, ∗∗*p* < 0.01, ∗∗∗*p* < 0.001.

We next confirmed that Hdac5 is directly related to Egr-1 expression. Depletion of *Hdac5* mRNA expression significantly increased the *Egr-1* mRNA level in 4-PBA-treated C2C12 cells (Figure 5D), suggesting that Hdac5 negatively regulates *Egr-1* expression. We next performed an immunoblotting analysis to examine the protein levels of Egr-1 and NF-κB p65, as well as histone H3 acetylation at Lys18 and Lys27, which are known to be upregulated by 4-PBA treatment. The results revealed that the protein expression of Egr-1 and NF-κB p65, along with the acetylation of histone H3 at Lys18 and Lys27 (Figures 5E and 5F), was significantly greater in *Hdac5*-knockdown cells than in control cells. These results suggest that *HDAC5* knockdown in C2C12 cells enhances histone H3 acetylation and activates NF-κB signaling and Egr-1 expression, indicating that the mechanism is similar to that of 4-PBA.

To further test whether HDAC5 suppression is causally involved in the molecular effects of 4-PBA, we next performed HDAC5 overexpression experiments. C2C12 myoblasts were transiently transfected with an HDAC5 expression vector or an empty control vector and then treated with 4-PBA during myogenesis (Figure S9A). HDAC5 overexpression attenuated the 4-PBA-induced increase in Egr-1 and NF-κB p65 expression (Figures S9B and S9C), as well as the increase in histone H3 acetylation at Lys18 and Lys27 (Figures S9D and S9E). To further examine whether 4-PBA regulates local chromatin acetylation at a relevant Egr-1 regulatory region containing the P1 region, we performed ChIP-qPCR analysis of H3K18ac and H3K27ac enrichment at the *Egr-1* promoter. 4-PBA increased H3K18ac and H3K27ac enrichment at the *Egr-1* promoter, whereas HDAC5 overexpression attenuated this increase (Figure S9F). These results indicate that restoring HDAC5 expression counteracts, at least in part, the local chromatin acetylation and transcriptional responses induced by 4-PBA, supporting the causal involvement of HDAC5 suppression in 4-PBA-mediated NF-κB/Egr-1 activation.

To determine whether HDAC5 suppression is functionally linked to myoblast proliferation, we next performed a cell proliferation assay in *Hdac5*-knockdown C2C12 cells. *Hdac5* knockdown significantly increased myoblast proliferation, phenocopying the effect of 4-PBA treatment (Figure S9G). Importantly, 4-PBA treatment did not further enhance proliferation in *Hdac5*-knockdown cells, suggesting that 4-PBA and HDAC5 suppression promote myoblast proliferation through overlapping pathways. Conversely, transient HDAC5 overexpression attenuated the 4-PBA-induced increase in myoblast proliferation, supporting the functional involvement of HDAC5 suppression in the proliferative response to 4-PBA (Figure S9H). Together with the HDAC5 overexpression results, these findings indicate that HDAC5 suppression contributes substantially to 4-PBA-induced histone H3 acetylation, NF-κB/Egr-1 activation, and the proliferation-promoting effect of 4-PBA.

### 4-PBA regulated ECM organization-related genes via Egr-1

To investigate the mechanism through which Egr-1 affects myogenic differentiation, the gene expression profiles of C2C12 cells with or without 4-PBA were determined via RNA-seq. Gene Ontology (GO) enrichment analysis revealed that the significantly enriched functional categories among the upregulated genes included positive regulation of external encapsulating structure organization, ECM organization, extracellular structure organization, and collagen aggregation (Figure 6A; Data S5). Furthermore, network analysis using GO enrichment revealed genes significantly involved in ECM structural constituent, integrin binding, and cell adhesion molecule binding (Figure 6B). In particular, the expression of collagen-related genes, which encode structural components of the ECM, increased during myoblast differentiation (Figure S10A; Data S6), suggesting that ECM-associated genes play important roles in muscle differentiation.

**Figure 6:**
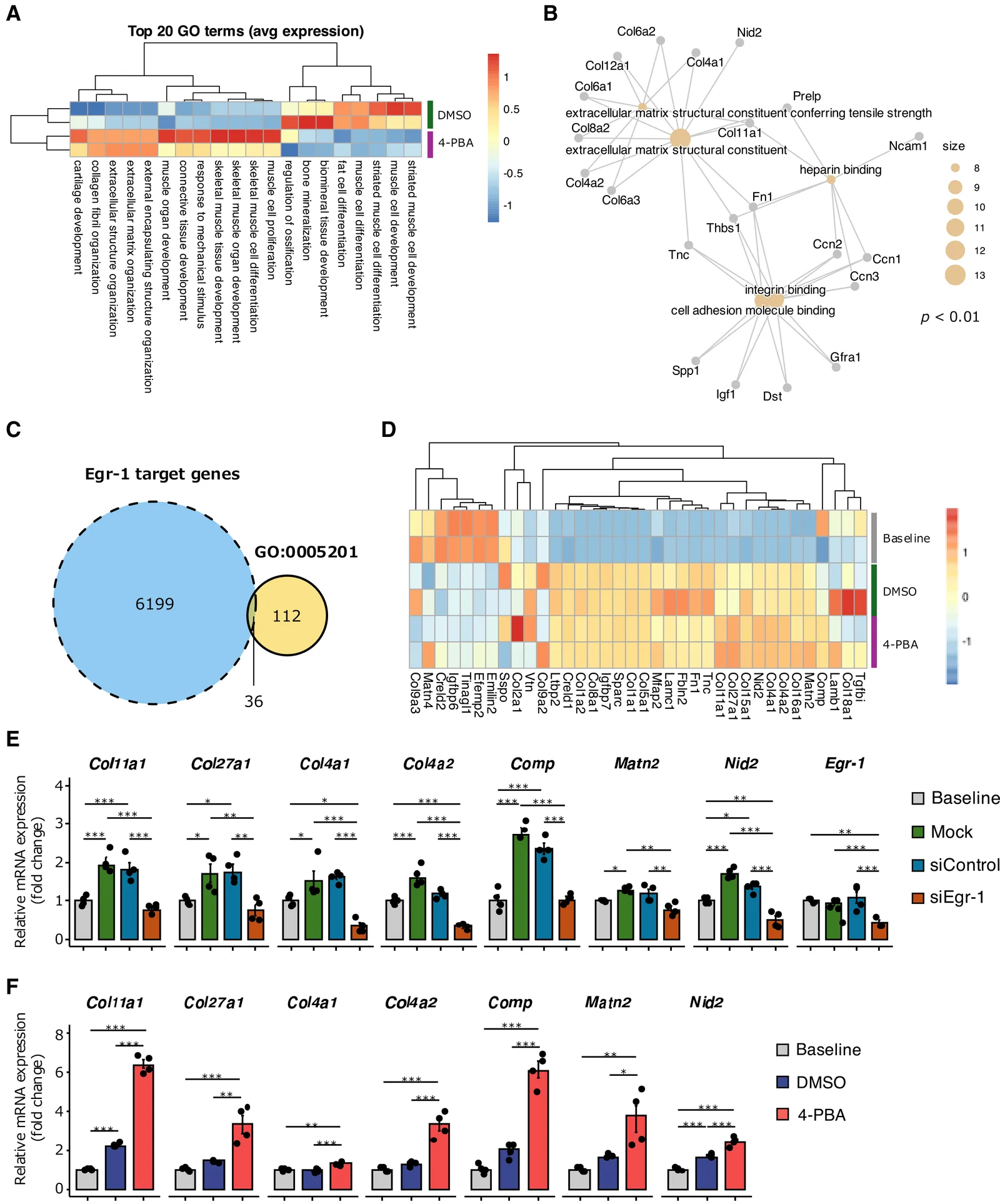
Egr-1 mediates 4-PBA-dependent transcriptional regulation of extracellular matrix organization genes (A) Heatmap of Gene Ontology (GO) hallmark gene sets with the top 20 lowest *p* values identified by parametric gene set enrichment analysis (GSEA) from RNA-seq data of C2C12 cells treated with 1 mM 4-PBA or DMSO. (B) Network visualization illustrating the functional connectivity between differentially expressed genes (DEGs) and enriched biological processes, highlighting extracellular matrix (ECM) organization-related pathways regulated by 4-PBA. Node size corresponds to the number of genes within each enriched pathway. (C) Venn diagram showing overlap between genes associated with the ECM structural constituent GO term (GO:0005201) and predicted Egr-1 target genes obtained from the ENCODE Transcription Factor Targets Dataset. (D) Heatmap showing expression patterns of Egr-1 target genes within the ECM structural constituent gene set, demonstrating coordinated regulation downstream of 4-PBA treatment. (E) Relative mRNA expression of selected ECM-related genes in C2C12 cells transfected with siEgr-1 (15 nM), siControl, or mock control, showing Egr-1-dependent transcriptional regulation. Expression levels were normalized to β-actin (Actin). Data represent four independent experiments. (F) Relative mRNA expression of selected ECM-related genes in C2C12 cells treated with 1 mM 4-PBA or DMSO under differentiation conditions. Baseline indicates expression levels prior to differentiation induction. Data represent four independent experiments. Data in (E) and (F) are presented as mean ± SEM. and analyzed using a one-way ANOVA with Tukey’s post hoc test. Each dot represents one independent experiment. ∗*p* < 0.05, ∗∗*p* < 0.01, ∗∗∗*p* < 0.001.

We next identified the ECM-related genes regulated by Egr-1 expression that were upregulated by 4-PBA treatment in C2C12 cells. A Venn diagram was constructed to examine the intersection between Egr-1 target genes based on the ENCODE Transcription Factor Targets Dataset^35^ and ECM structural components (GO:0005201), indicating that a total of 36 common genes were identified (Figure 6C). On the other hand, we found that there were no genes common between Egr-1 target genes and categories for the adhesion molecule binding (GO:0050839), integrin binding (GO:0005178), and heparin binding (GO:0008201) that the network analysis using GO enrichment revealed (Figure S10B). To identify the genes linking 4-PBA to the upregulation of ECM via Egr-1 expression, we displayed the main representative up-regulated genes on a heatmap, and the expression of Egr-1 target genes by 4-PBA increased gradually from left to right (Figure 6D; Data S7). Changes in gene expression following *Egr-1* knockdown were confirmed using quantitative reverse-transcription PCR (RT-qPCR). Several ECM-related genes, including *collagen type XI alpha 1 (Col11a1)*, *collagen type* *XXVII* *alpha 1 (Col27a1)*, *collagen type IV alpha 1 (Col4a1)*, and *collagen type IV alpha 2 (Col4a2)*, as well as genes involved in the production of collagen, such as *cartilage oligomeric matrix protein (Comp)* and *myogenesis-related genes, including* *matrilin-2* *(Matn2)* and *nidogen-2* *(Nid2)*, a crucial basement membrane (BM) protein in muscle, were among the most significantly downregulated in *Egr-1*-knockdown C2C12 myoblasts compared with those in siControl-transfected cells or at baseline levels (Figure 6E). Moreover, the expression of these genes was significantly greater in C2C12 myoblasts treated with 4-PBA than in those treated with DMSO (Figure 6F). Similar upregulation of these genes was also observed in *Hdac5*-knockdown cells (Figure S10C). Importantly, HDAC5 overexpression attenuated the 4-PBA-induced upregulation of these collagen-related and ECM-associated genes (Figure S10D). These results further support the idea that suppression of HDAC5 contributes to 4-PBA-induced ECM gene expression through the activation of the Egr-1-dependent transcriptional program. Overall, these results suggest that 4-PBA promotes ECM remodeling and the expression of muscle-related genes and that Egr-1 is involved in regulating the expression of these genes.

## Discussion

Although significant progress has been made in understanding muscle differentiation, many of the underlying mechanisms remain unresolved. Here, we report that 4-PBA regulates myoblast differentiation, cell proliferation, and ECM remodeling via epigenetic and transcriptional mechanisms, advancing our understanding of muscle pathology and facilitating the development of novel therapeutic strategies for muscle-related diseases such as sarcopenia and muscular dystrophy.

We found that 4-PBA acts as an epigenetic regulator during myoblast differentiation. Transcriptomic screening and immunoblotting analysis revealed that histone H3 acetylation (H3K18ac and H3K27ac) increased during early differentiation, supporting the role of 4-PBA as an effective inhibitor of Hdac5 in C2C12 myoblasts. We demonstrate that compared with many HDAC inhibitors that reinforce muscle-specific transcription, 4-PBA inhibits myoblast differentiation and the expression of the myogenic-related genes *Myod* and *Dysf* to regulate muscle differentiation. Importantly, 4-PBA selectively induced the expression of NF-κB p65 via inhibition of Hdac5 expression. Consistent with this model, HDAC5 overexpression attenuated the 4-PBA-induced increase in Egr-1 and NF-κB p65 expression, as well as histone H3 acetylation at Lys18 and Lys27. In addition, ChIP-qPCR analysis showed that 4-PBA increased H3K18ac and H3K27ac enrichment at the *Egr-1* promoter, whereas HDAC5 overexpression attenuated this increase. These results further support the idea that suppression of HDAC5 contributes to 4-PBA-induced local chromatin acetylation and NF-κB/Egr-1 activation during early myogenesis. NF-κB plays complex roles in skeletal muscle differentiation through opposing signaling pathways.^36^ The canonical NF-κB signaling pathway, via RelA/p65, IKKβ, and IKKγ, is a key transcription factor that generally promotes muscle cell proliferation, especially in skeletal MuSCs after chronic injury.^37^^,^^38^^,^^39^ We propose that 4-PBA promotes cell proliferation by activating the NF-κB signaling pathway, thereby contributing to myoblast expansion during early myogenesis following injury.

We identified HDAC5 as a key epigenetic regulator during early myogenesis and suggest that its suppression by 4-PBA contributes to altered histone acetylation and transcriptional dysregulation. We also suggest that 4-PBA targets the acetylation of histone H3 but not NF-κB p65 directly through HDAC5 inhibition. In addition to the *Hdac5* knockdown experiments, HDAC5 overexpression attenuated the 4-PBA-induced increase in myoblast proliferation, supporting the functional involvement of HDAC5 suppression in the proliferative response to 4-PBA. These gain- and loss-of-function data suggest that HDAC5-dependent chromatin regulation is a major contributor to 4-PBA-induced myoblast proliferation, although they do not exclude the involvement of additional 4-PBA-sensitive pathways. In fact, fasting or several types of muscular dystrophy increase p65 acetylation at Lys310, activating NF-κB and contributing to muscle atrophy.^40^ Acetylated NF-κB p65 also promotes the expression of inflammatory genes, a hallmark of muscle wasting.^40^^,^^41^ In acute muscle injury, inflammation is necessary but must be precisely resolved to avoid chronic damage.^42^ Notably, long-term 4-PBA treatment during myogenesis reduces the phosphorylation of NF-κB p65 gradually as myotubes mature, which is activated in the early stage (Figures 3A and 3B). These findings suggest that myogenesis-related factors may contribute to a negative feedback loop that controls the accumulation of NF-κB p65 signaling. This process contributes to the suppression of excessive activation of the NF-κB signaling pathway. 4-PBA can act downstream of many signaling cascades, allowing regeneration to proceed without broadly activating NF-κB-driven inflammatory pathways that can exacerbate tissue damage.

We identified Egr-1 expression as a key factor that alternates between cell proliferation and differentiation in myoblasts, and we functionally characterized the regulation of Egr-1 expression by NF-κB p65. Egr-1 is a broadly responsive immediate-early transcription factor that can be induced by multiple upstream stimuli, including growth factor signaling, hypoxia, oxidative stress, mechanical stretch, and exercise-related cues. For example, Pardo et al. demonstrated that mechanical stretch induces an Egr-1-dependent transcriptional program in skeletal muscle cells, leading to Sirt1, an NAD-dependent protein deacetylase, induction and an antioxidative response.^43^ Therefore, Egr-1 should not be interpreted as a 4-PBA-specific downstream factor, but rather as a convergence point for multiple regulatory pathways that may be engaged during myogenic cell-state remodeling. In our study, Egr-1 induction was detected 2–3 days after 4-PBA treatment, rather than at an early acute time point, suggesting that this response is unlikely to reflect only a transient acute stress response. Instead, Egr-1 may represent a secondary transcriptional response associated with HDAC5-dependent chromatin remodeling, NF-κB activation, and maintenance of a proliferative, less differentiated myogenic state. Future studies using detailed time-course analyses and pathway-specific perturbation experiments will be required to determine whether Egr-1 functions as a sustained transcriptional mediator or as a downstream marker of the altered myogenic cell state induced by 4-PBA treatment.

We found that 4-PBA induces Egr-1-regulated ECM- and collagen-related gene expression. Collagen provides structural stability to muscle fibers and the surrounding connective tissues, thereby contributing to myoblast differentiation, fusion, and proper structural organization.^44^ In most muscular dystrophies, the accumulation of collagen, particularly type I, type III, and type IV collagens, is markedly increased.^45^^,^^46^^,^^47^^,^^48^ Excessive collagen deposition within the ECM, which is produced mainly by fibroblasts, progressively leads to fibrosis and impaired muscle function. In contrast, specific collagen types—primarily nonfibrillar collagens—serve as essential components of the MuSC microenvironment and regulate their behavior. In our study, we detected increased expression of *Col27a1*, *Col4a1*, and *Col4a2*, which are known ECM components that support normal muscle development, regeneration, and resistance to mechanical stress. Notably, Col27a1 is expressed in the MuSC niche, suggesting a potential role in regulating the local microenvironment.^2^ Type IV collagen is a key component of the BM, which surrounds satellite cells together with laminin and interacts with surface α7/β1 integrins.^49^^,^^50^ 4-PBA induces the expression of genes involved in collagen production, namely, *Matn2* and *Nid2*. Matn2 functions as a key regulator of myogenic program initiation by modulating BMP7 and integrin α5 signaling, inducing the expression of the Cdk inhibitor p21, and activating myogenic genes, including *Nfix*, *MyoD*, and *Myog*.^51^ Nid2 has been detected in conditioned media from differentiating human myoblasts by mass spectrometry, which suggests that it is secreted as a soluble protein during myogenesis.^52^^,^^53^ Nid2 is also a component of the muscle basal lamina and plays an important role in neuromuscular junction development.^51^^,^^54^ Importantly, HDAC5 overexpression attenuated the 4-PBA-induced upregulation of collagen-related and ECM-associated genes. These findings further support the idea that HDAC5 suppression contributes to the 4-PBA-induced ECM transcriptional program through Egr-1 activation. Collectively, these results indicate that 4-PBA promotes ECM gene expression via the HDAC5-NF-κB p65-Egr-1 signaling axis, thereby facilitating the expansion of undifferentiated muscle cells and generating replacement cells capable of responding to injury-associated cues. Moreover, in 4-PBA-treated skeletal muscle cells, these specialized ECMs may regulate signaling activities and maintain satellite cells in an undifferentiated state.

In addition to ECM regulation, Egr-1 may also be relevant to organelle remodeling and mitochondrial regulatory pathways. This point is important because 4-PBA reduced the expression of mitochondrial regulatory factors, including OPA1 and PGC1α, in our experimental system. OPA1 regulates mitochondrial inner membrane fusion and cristae remodeling,^55^ whereas PGC1α is a key regulator of mitochondrial biogenesis and oxidative metabolic programs.^56^ Parra et al. demonstrated that insulin promotes mitochondrial fusion, membrane potential, ATP production, and oxygen consumption through an Akt-mTOR-NF-κB-OPA1 signaling axis in rat cardiomyocytes and skeletal muscle cells, indicating that NF-κB signaling can influence mitochondrial dynamics through OPA1-dependent remodeling.^32^ Based on this framework, the 4-PBA-induced NF-κB-Egr-1 response may intersect with mitochondrial regulatory pathways. However, our JC-1 mitochondrial membrane potential assay did not reveal significant differences between DMSO-treated control cells and 4-PBA-treated cells in either myoblasts or myotubes. Thus, although 4-PBA altered OPA1 and PGC1α expression, these transcriptional or protein-expression changes were not accompanied by overt mitochondrial depolarization or hyperpolarization detectable by JC-1 under our experimental conditions. Therefore, the Egr-1-NF-κB-related pathway should be considered a potential regulatory link between chromatin remodeling, stress-response signaling, and mitochondrial adaptation, rather than direct evidence of functional mitochondrial remodeling. Future studies using mitochondrial morphology analysis, mtDNA copy number, and oxygen consumption measurements will be required to determine whether these molecular changes lead to altered mitochondrial mass, dynamics, or respiratory function.

HDAC inhibitors such as trichostatin A (TSA), sodium butyrate (NaB), and valproic acid (VPA) have been shown to influence myoblast differentiation by increasing histone acetylation and activating muscle-specific gene expression in muscular atrophy and muscular dystrophy models.^57^^,^^58^^,^^59^ Recently, givinostat, known as DUVYZAT, which is an HDAC inhibitor for HDAC1 and HDAC3, was approved for patients with Duchenne muscular dystrophy and has the potential to restore dystrophin expression, reduce inflammation, and preserve muscle integrity.^18^ Previous studies have shown that 4-PBA helps to restore the membrane localization of the DYSF protein and repair the sarcolemma in a mouse model of dysferlinopathy.^27^ Our data also indicate that 4-PBA controls cell proliferation and differentiation by targeting histone H3 without hyperactivation of inflammatory pathways, including the NF-κB signaling pathway. Collectively, these findings, together with our present results, suggest that 4-PBA plays an important role in myogenic differentiation after injury through the regulation of gene expression related to ECM remodeling and the alleviation of ER stress, potentially contributing to the prevention of muscle diseases. Because 4-PBA also reduced selected ER stress markers in our study, including GRP78, phosphorylated eIF2α, phosphorylated IRE1, and CHOP, its effects likely reflect overlapping mechanisms involving HDAC5-dependent epigenetic regulation and stress-response modulation rather than a single linear pathway. However, TUDCA did not reproduce the proliferation-promoting effect of 4-PBA, suggesting that ER stress attenuation alone is insufficient to explain the observed myoblast expansion.

In summary, this work establishes a role for the HDAC inhibitor 4-PBA in maintaining muscle homeostasis and identifies HDAC5 suppression, Egr-1 expression, and NF-κB p65 as key mechanisms for promoting myoblast proliferation and ECM gene expression. Our additional gain-of-function experiments further show that HDAC5 overexpression attenuates 4-PBA-induced chromatin acetylation, NF-κB/Egr-1 activation, collagen-related gene expression, and myoblast proliferation. These findings support the conclusion that HDAC5-dependent chromatin regulation is a major mechanism underlying the biological effects of 4-PBA, while additional 4-PBA-sensitive pathways, including ER stress modulation, may also contribute. Future studies are warranted to determine such potential clinical applications.

### Limitations of the study

Despite the mechanistic insights gained in this study, several limitations should be acknowledged, along with potential strategies to address them in future work. First, although we identified HDAC5 inhibition as a primary mechanism underlying 4-PBA-mediated effects on histone acetylation, NF-κB signaling, and ECM gene expression, 4-PBA is a multifunctional compound with pleiotropic biological activities. Our findings indicate that chemical chaperone activity and ER stress alleviation may act in parallel with epigenetic regulation. Dissecting these mechanisms will require complementary genetic approaches, including ER stress-specific modulators. Second, our analysis of NF-κB signaling focused primarily on early myogenesis, where transient activation appears to promote myoblast proliferation. However, prolonged or excessive NF-κB activation is associated with chronic inflammation and muscle wasting. Long-term *in vivo* studies are therefore needed to determine whether 4-PBA maintains a beneficial balance between regenerative signaling and inflammatory resolution during aging or chronic muscle disease. Third, although we validated key findings in non-immortalized primary myogenic cells, these experiments were performed using cells isolated from soleus muscle only. Because soleus is predominantly an oxidative muscle, our primary cell data do not fully address whether the effects of 4-PBA are conserved across muscles with different fiber-type compositions, such as mixed or glycolytic muscles. Moreover, only male mice were used in this study; therefore, potential sex-dependent differences could not be evaluated. Finally, although we observed increased expression of ECM- and collagen-related genes associated with a supportive satellite cell niche, we did not directly assess ECM architecture, biomechanical properties, or interactions with fibroblasts and immune cells. Future studies combining histological, biophysical, and single-cell approaches will help clarify whether 4-PBA-induced ECM remodeling favors regeneration without promoting fibrosis. Together, addressing these limitations will be critical for translating 4-PBA-based strategies toward safe and effective therapies for muscle injury and degenerative diseases.

## Resource availability

### Lead contact

Requests for further information and resources and reagents should be directed to and will be fulfilled by the lead contact, Kana Tominaga (kanat@yamaguchi-u.ac.jp).

### Materials availability

This study does not report newly generated materials.

### Data and code availability

• RNA-sequencing data generated in this study have been deposited at GEO under accession number GSE315556 and are publicly available as of the date of publication.
• This paper does not report original code.
• Any additional information required to reanalyze the data reported in this paper is available from the lead contact upon request.

## Acknowledgments

We thank Dr. S. Nakagawa for providing a supportive research environment. We thank T. Ogata for helping in RNA-seq data analysis and A. Kobayashi for supporting general lab work. We thank Science Research Center, Institute of Life Science and Medicine for animal research, and the Yamaguchi University Center for Gene Research for using Imaging system, Microplate reader, and Real-time PCR system. This work was supported by 10.13039/501100007263Astellas Foundation for Research on Metabolic Disorders (to K.T.), 10.13039/100008732Uehara Memorial Foundation (to K.T.), New Frontier Research Grant by 10.13039/100016983Yamaguchi University (to K.T. and N.T.), Finding-Out & Crystallization of Subliminals (FOCS) project by Yamaguchi University of Medicine (to K.T. and N.T.). This work was supported in part by a Grant-in-Aid for Research Activity Start-up (21K21219), Early-Career Scientists (22K17826) from Japan Society of the Promotion of Science (10.13039/501100001691JSPS) (to N.T.), 10.13039/501100020962ACT-X (JPMJAX222B) from 10.13039/501100002241Japan Science and Technology Agency (JST) (to N.T.), the Ube City Next-Generation Researchers Project (to N.T.). This work was the result of using research equipment shared in 10.13039/501100001700MEXT Project for promoting public utilization of advanced research infrastructure (program for supporting construction of core facilities), grant no. JPMXS0440400023.

## Author contributions

K.T. conceived and designed the study. K.T. and N.T. performed experiments and did the data analysis and interpretation. K.T. wrote the manuscript and prepared the figures. K.T. and N.T. reviewed and edited the paper.

## Declaration of interests

The author N.T. is a founder and director of ADDVEMO Inc. ADDVEMO Inc. was not involved in the study design, data collection, analysis, interpretation of data, the writing of the report, or the decision to submit the article for publication.

## STAR★Methods

### Key resources table

**Table:** 

REAGENT or RESOURCE	SOURCE	IDENTIFIER
**Antibodies**		

Rabbit anti-Egr-1	Proteintech	Cat #: 55117-1-AP; RRID: AB_2881272
Mouse anti-Fos	Proteintech	Cat #: 66590-1-IG; RRID: AB_2881950
Rabbit anti-MYH1, clone 1E15	Sigma-Aldrich	Cat #: ZRB1214; RRID: AB_3083662
Rabbit anti-MYH7, clone 1B19	Sigma-Aldrich	Cat #: ZRB1308
Mouse anti-NCL-Hamlet DYSF	Leica Biosystems	Cat #: NCL-Hamlet; RRID: AB_442097
Mouse anti-Myogenin	BD Biosciences	Cat #: 556358; RRID: AB_396383
Rabbit anti-Desmin	Proteintech	Cat #: 16520-1-AP; RRID: AB_2292918
Rabbit anti-β-Actin	GeneTex	Cat #: GTX110564; RRID: AB_10618080
Rabbit anti-Phospho- ERK1/2 (Thr202/Tyr204)	Proteintech	Cat #: 28733-1-AP; RRID: AB_2881202
Rabbit anti-Erk1/2	Proteintech	Cat #: 11257-1-AP; RRID: AB_2139822
Rabbit anti-Phospho-NF-κB p65 (Ser468)	Proteintech	Cat #: 82335-1-RR; RRID: AB_3083091
Rabbit anti-Phospho-NF-kB p65 (Ser536)	Cell Signaling Technology	Cat #: 3033; RRID: AB_331284
Rabbit anti-Acetyl-NFkB p65 (Lys310)	Invitrogen	Cat #: PA5-17264; RRID: AB_10983502
Rabbit anti-NF-κB p65	BioLegend	Cat #: 622602; RRID: AB_315956
Rabbit anti-Histone H3 (D1H2)	Cell Signaling Technology	Cat #: 4499; RRID: AB_10544537
Rabbit anti-Acetyl-Histone H3 (Lys9) (C5B11)	Cell Signaling Technology	Cat #: 9649; RRID: AB_823528
Rabbit anti-Acetyl-Histone H3 (Lys14) (D4B9)	Cell Signaling Technology	Cat #: 7627; RRID: AB_10839410
Rabbit anti-Acetyl-Histone H3 (Lys18) (D8Z5H)	Cell Signaling Technology	Cat #: 13998; RRID: AB_2783723
Rabbit anti-Acetyl-Histone H3 (Lys27) (D5E4)	Cell Signaling Technology	Cat #: 8173; RRID: AB_10949503
Rabbit anti-Acetyl-Histone H3 (Lys56)	Cell Signaling Technology	Cat #: 4243; RRID: AB_10548193
Rabbit anti-HDAC5 (D1J7V)	Cell Signaling Technology	Cat #: 20458; RRID: AB_2713973
Rabbit anti-Phospho-eIF2α (Ser51) (D9G8)	Cell Signaling Technology	Cat #: 3398T; RRID: AB_2096481
Rabbit anti-eIF2α (D7D3)	Cell Signaling Technology	Cat #: 5324T; RRID: AB_10692650
Rabbit anti-Grp78	GeneTex	Cat #: GTX113340; RRID: AB_11173017
Rabbit anti-ATF4	GeneTex	Cat #: GTX101943; RRID: AB_1240487
Rabbit anti-GADD153 (CHOP)	GeneTex	Cat #: GTX112827
Rabbit anti-IRE1 (pSer724)	GeneTex	Cat #: GTX132808; RRID: AB_2886751
Rabbit anti-IREα (14C10)	Cell Signaling Technology	Cat #: 3294T; RRID: AB_823545
Mouse anti-ATF6 Antibody (70B1413.1)	Novus Biologicals	Cat #: NBP1-40256; RRID: AB_2058774
Rabbit anti-Pax7 [EPR30466-531]	abcam	Cat #: ab324017
Rabbit anti-OPA1 (D6U6N)	Cell Signaling Technology	Cat #: 80471T; RRID: AB_2734117
Rabbit anti-DRP1 (C-terminal)	Proteintech	Cat #: 12957-1-AP; RRID: AB_2093525
Mouse anti-PGC1α	Proteintech	Cat #: 66369-1-IG; RRID: AB_2828002
Goat anti-rabbit IgG, HRP-linked Antibody	Cell Signaling Technology	Cat #: 7074S; RRID: AB_2099233
Horse anti-mouse IgG, HRP-linked Antibody	Cell Signaling Technology	Cat #: 7076S; RRID: AB_330924
Goat anti-Rabbit IgG H&L (Alexa Fluor 488)	abcam	Cat #: ab150077; RRID: AB_2630356
Goat anti-Mouse IgG H&L (Alexa Fluor 488)	abcam	Cat #: ab150113; RRID: AB_2576208
Purified anti-Rabbit IgG	BioLegend	Cat #: 410404; RRID: AB_2632606

**Chemicals, peptides, and recombinant proteins**		

4-Phenylbutyric acid (4-PBA)	Sigma-Aldrich	Cat #: P21005-25G
Block Ace blocking reagent	DS Pharma Biomedical	Cat #: UK-B80
Bovine serum albumin (BSA)	Nacalai tesque	Cat #: 01281-26
Collagenase type 1	Worthington Biochemical Corporation	Cat #: CLS1
cOmplete ULTRA Tablets, Mini, EDTA-free, EASYpack	Roche	Cat #: 5892791001
DAPI (4′,6-diamidino-2-phenylindole) Solution	Dojindo	Cat #: D523
Dimethyl sulfoxide (DMSO)	Nacalai tesque	Cat #: 13408-64
(−)-DHMEQ	MedChemExpress	Cat #: HY-14645
Dulbecco’s Modified Eagle Medium (DMEM), high glucose	Nacalai tesque	Cat #: 08459-64
Ethanol	FUJIFILM Wako Pure Chemical Corporation	Cat #: 054-07225
Fetal bovine serum (FBS) (Lot. 0001641439)	Nichirei Biosciences	Cat #: 173012-500ML
Glycine	Nacalai tesque	Cat #: 17109-35
Hanks’ Balanced Salt Solution/HBSS	Nacalai Tesque	Cat #: 17461-05
HCl	FUJIFILM Wako Pure Chemical Corporation	Cat #: 084-05425
Horse serum (HS)	GIBCO	Cat #:26050088
Immobilon Western Chemiluminescent HRP Substrate	Cytiva	Cat #: RPN2235
Lipofectamine RNAiMAX transfection reagent	Invitrogen	Cat #: 13778075
Hilymax	Dojindo	Cat #: H357
M-PER Mammalian Protein Extraction Reagent	Thermo Fisher	Cat #: 78501
Matrigel	Corning	Cat #: 354234
NaCl	FUJIFILM Wako Pure Chemical Corporation	Cat #: 191-01665
Paraformaldehyde (37% PFA, for crosslinking)	FUJIFILM Wako Pure Chemical Corporation	Cat #: 063-04815
Paraformaldehyde (4% PFA)	FUJIFILM Wako Pure Chemical Corporation	Cat #:163-20145
Penicillin-Streptomycin Mixed Solution	Nacalai tesque	Cat #: 26253-84
Phosphatase Inhibitor Cocktail Solution Ⅱ(×100)	FUJIFILM Wako Pure Chemical Corporation	Cat #: 160-24371
Phosphate-buffered saline (PBS, pH 7.4)	Nacalai tesque	Cat #: 14249-24
ProLong Glass Antifade Mountant	Thermo Fisher	Cat #: P36965
Protein A Mag Sepharose	Cytiva	Cat #: 28944006
PicoLab Rodent Diet 20	LabDiet	Cat #: 5053
Red Blood Cell (RBC) Lysis Buffer/RCK buffer	BioLegend	Cat #: 420301
Sample Buffer Solution with Reducing Reagent(6×) for SDS-PAGE pH6.8	Nacalai tesque	Cat #: 09499-14
Sodium Dodecyl Sulfate	FUJIFILM Wako Pure Chemical Corporation	Cat #: 199-07141
Tauroursodeoxycholic Acid (TUDCA)	Selleck Biotech	Cat #: S3654
Triton X-100	Sigma-Aldrich	Cat #: 9002-93-1
Trypsin-EDTA (0.25%)	Nacalai tesque	Cat #: 35554-64
Tunicamycin	Cayman Chemical	Cat #: 11445
Tween 20	Sigma-Aldrich	Cat #: P9416-100ML
Silencer Select Mouse Hdac5 siRNA (Assay ID s67423)	Invitrogen	Cat #: 4390771
Silencer Select Mouse Egr-1 siRNA (Assay ID s65378)	Invitrogen	Cat #: 4390771
Silencer Select Negative Control No. 1 siRNA	Invitrogen	Cat #: 4390843

**Critical commercial assays**		

Agilent 4200 TapeStation D1000 assay	Agilent	Cat #: D1000
CellTiter 96 AQ_ueous_ One Solution Cell Proliferation Assay (MTS)	Promega	Cat #: G3582
ISOSPIN Cell & Tissue RNA kit	Nippon Gene	Cat #: 314-08211
JC-1 MitoMP Detection Kit	Dojindo	Cat #: MT09
Maxwell RSC Simply RNA Cell Kit	Promega	Cat #: AS1390
Micro BCA Protein Assay Kit	Thermo Scientific	Cat #: 23235
NEBNext Poly(A) mRNA Magnetic Isolation Module	New England BioLabs	Cat #: E7490
NEBNext Ultra II Directional RNA Library Prep Kit	New England BioLabs	Cat #: E7490
NovaSeq 6000 S1 Reagent Kit v1.5	Illumina	Cat #: 20028319
PowerTrack SYBR Green Master Mix for qPCR	Thermo Fisher Scientific	Cat #: A46109
ReverTra Ace qPCR RT Master Mix with gDNA remover	Toyobo	Cat #: FSQ-301
SimpleChIP Enzymatic Chromatin IP Kit Magnetic Beads	Cell Signaling Technology	Cat #: 9003
TapeStation 4200 High Sensitivity RNA assay	Agilent Technologies	Cat #: 5067-5579

**Deposited data**		

Raw and analyzed data	This paper	GSE315556

**Experimental models: Cell lines**		

Mouse: C2C12 cell	ATCC	Cat #: CRL-1772

**Experimental models: Organisms/strains**		

Mouse: C57BL/6NCrSlc male mice, 6 weeks old	Japan SLC	N/A

**Oligonucleotides**		

Primers used in this study	This paper	See Tables S2 and S3

**Recombinant DNA**		

pRP[Exp]-EGFP-CMV>mHdac5[NM_001405232.1]	VectorBuilder	pDNA(VB260611-1700fpe)
pRP[Exp]-CMV>EGFP/*Neo*	VectorBuilder	pDNA(VB010000-9487ngd)-P

**Software and algorithms**		

R software (version 4.5.1)	R Foundation	https://www.r-project.org
R studio (version 2025.05.0 + 496)	R Core Team	Posit Software, PBC, Boston, MA. https://www.posit.co/
seqinr R package	Charif et al. 2007 ^60^	https://CRAN.R-project.org/package=seqinr
clusterProfiler R package (version 4.16.0)	Yu et al. 2012 ^61^	https://bioconductor.org/packages/clusterProfiler/
org.Mm.e.g.,.db R package (version 3.21.0)	Carlson 2025 ^62^	https://bioconductor.org/packages/org.Mm.eg.db/
KEGGREST R package (version 1.48.1)	Tenenbaum 2025 ^63^	https://bioconductor.org/packages/KEGGREST
GO.db R package (version 3.21.0)	Carlson 2025 ^64^	https://bioconductor.org/packages/GO.db/
ggplot2 R package (version 4.0.0)	Wickham 2016 ^65^	https://ggplot2.tidyverse.org
ggpubr R package (version 0.6.1)	Kassambara 2023 ^66^	https://rpkgs.datanovia.com/ggpubr/
pheatmap R package (version 1.0.13)	Kolde 2019 ^67^	https://CRAN.R-project.org/package=pheatmap
VennDiagram R package (version 1.7.3)	Chen et al. 2011 ^68^	https://github.com/uclahs-cds/package-VennDiagram
QuantStudio Design & Analysis Software	Thermo Scientific	N/A
SoftMax Pro Software	Molecular Devices	N/A
Amersham Imager 680 Analysis Software version 2.0	Cytiva	N/A
BZ-H1A	Keyence	N/A
CLC Genomics Workbench	Qiagen	N/A
Fiji (ImageJ2, Version 2.16.0/1.54p)	Cell Imaging Core at the Turku Center for Biotechnology	https://imagej.net/software/fiji/

### Experimental model and study participant details

#### Mice

Mouse experiments were performed under protocols approved by the Yamaguchi University Animal Care and Use Committee (No.80–002). Six-week-old male C57BL/6NCrSlc mice were used in this study. Mice were group-housed in ventilated cages under controlled conditions at 25°C and 30–70% humidity with a 12-h light/dark cycle. Water and PicoLab Rodent Diet 20 (LabDiet) were provided *ad libitum*.

#### Primary murine myoblasts

Primary murine myoblasts were isolated from the soleus muscles of 6-week-old male C57BL/6NCrSlc mice as described below. Cells isolated from individual mice were maintained and analyzed independently as biological replicates. Cells were maintained in myoblast growth medium consisting of Dulbecco’s Modified Eagle Medium (DMEM, Nacalai tesque) supplemented with 10% fetal bovine serum (FBS, Nichirei Biosciences), 100 U/mL penicillin, and 100 μg/mL streptomycin (Nacalai tesque) at 37°C in a humidified atmosphere containing 5% CO_2_. Primary myoblasts were used before reaching overconfluence and were passaged as required for subsequent experiments.

#### C2C12 myoblasts

C2C12 murine myoblasts were purchased from the American Type Culture Collection (ATCC). C2C12 cells and primary myoblasts were maintained in myoblast growth medium consisting of DMEM supplemented with 10% FBS, 100 U/mL penicillin, and 100 μg/mL streptomycin. C2C12 cells were cultured in a humidified atmosphere at 37°C in 5% CO_2_, and the culture medium was changed every 2 days. C2C12 cells were tested for mycoplasma contamination and confirmed to be negative. The cells were not independently authenticated after purchase from ATCC.

### Method details

#### Isolation of murine primary myoblasts

Primary myoblasts were isolated from the soleus muscles of mice under sterile conditions. Briefly, soleus muscles were excised and immediately transferred into cold DMEM. Visible fat, connective tissue, tendons, and blood vessels were carefully removed using sterile forceps and scissors. The cleaned muscle tissues were minced into small fragments using sterile scissors. The minced tissues were then digested in 0.2% collagenase type 1 (Worthington Biochemical Corporation) solution prepared in DMEM at 37°C for 1 h with gentle agitation. After enzymatic digestion, the cell suspension was passed through a 100-μm cell strainer to remove undigested tissue debris. To remove residual whole blood, the cell suspension was incubated with Red Blood Cell (RBC) Lysis Buffer (BioLegend) at room temperature for 10 min, followed by washing with DMEM. The resulting cell pellet was resuspended in myoblast growth medium.

#### Cell culture and passaging

When cells reached subconfluence, they were rinsed in phosphate-buffered saline (PBS, pH 7.4, Nacalai tesque), trypsinized with 0.25% trypsin-EDTA solution (Nacalai tesque) for 5 min at 37°C, and passaged at ratios of 1:4-1:8.

For murine primary myoblasts culture, culture plates were coated with 0.8% Matrigel (Corning) diluted in DMEM before cell seeding. The isolated cells were plated onto Matrigel-coated dishes and cultured at 37°C in a humidified incubator with 5% CO_2_. After cell attachment, the medium was replaced to remove non-adherent cells and residual debris. Primary myoblasts were maintained in growth medium, and passaged or used for subsequent experiments before reaching overconfluence.

#### Myogenic differentiation

To induce the differentiation and cell fusion, C2C12 cells and primary myoblasts were washed with PBS, then changed to differentiation medium in DMEM containing 5% horse serum (HS, GIBCO), and 100 U/mL penicillin, and 100 μg/mL streptomycin. The differentiation medium was changed every day.

#### Reagent treatment

For 4-Phenylbutyric acid (4-PBA, Sigma-Aldrich) treatment, C2C12 myoblasts or murine primary myoblasts were cultured in differentiation medium containing 1 mM 4-PBA or vehicle control (DMSO, Nacalai tesque) for the indicated time periods. The treatment medium was replaced daily.

For NF-κB inhibition experiments, cells were treated with (−)-dehydroxymethylepoxyquinomicin (DHMEQ, MedChemExpress) at 2 or 10 μg/mL for 24 h under the diffetentiation condition. For ER stress modulation experiments, cells were treated with tauroursodeoxycholic acid (TUDCA, Selleck Biotech) at 1, 10, or 100 μM, or with Tunicamycin (Cayman Chemical) at 0.1, 0.5, or 1.0 μM, for the indicated time periods. Control cells were treated with the corresponding vehicle under the same culture conditions.

After treatment, cells were subjected to immunoblotting, immunofluorescence, quantitative PCR, cell proliferation assays, or JC-1 mitochondrial membrane potential assays, as indicated.

#### Small interfering RNA (siRNA)

For knockdown of *Hdac5* or *Egr-1*, we used Silencer Select Mouse Hdac5 (Assay ID s67423) or Mouse Egr-1 (Assay ID s65378) purchased from Invitrogen. C2C12 myoblasts were seeded at a density of 1 × 10^5^ cells/well into a 6-well plate and incubated with siRNAs (5 or 15nM) and Lipofectamine RNAiMAX transfection reagent (Invitrogen) according to the manufacturer’s protocol. The control was Silencer Select Negative Control No. 1 siRNA. siRNA-transfected cells were incubated at 37°C in 5% CO_2_ until the assay. Representative data from three independent experiments.

#### HDAC5 overexpression

The mouse Hdac5 expression vector pRP[Exp]-EGFP-CMV>mHdac5[NM_001405232.1] and the EGFP control vector pRP[Exp]-CMV>EGFP/*Neo* were generated by VectorBuilder. C2C12 myoblasts (2.5×10^5^ cells) were transiently transfected with the Hdac5 expression vector or control vector using Hilymax (Dojindo) according to the manufacturer’s instructions. For optimization, 1, 2, or 5 μg/well of plasmid DNA was tested, and HDAC5 protein expression was confirmed by immunoblotting. For subsequent experiments, cells were transfected with 5 μg/well of plasmid DNA. Twenty-four hours after transfection, cells were switched to differentiation medium containing vehicle control or 1 mM 4-PBA and collected at differentiation Day 2 for immunoblotting, ChIP-qPCR analyses and qPCR.

#### RNA sequence

C2C12 myoblasts were plated at 7.0 × 10^5^ cells in 60 mm dishes in growth medium. Once confluent, media was changed to differentiation medium with 1mM 4-PBA or DMSO as control and left to differentiate for 4 days. Differentiation medium was replenished daily.

We purified total RNA using an Maxwell RSC Simply RNA Cell Kit (Promega), followed by DNase treatment and assess the RNA purity using a Tapestation 4200 High Sensitivity RNA (Agilent Technologies).

To generate cDNA libraries for Illumina sequencing, we used The NEBNext Poly(A) mRNA Magnetic Isolation Module (New England BioLabs) and NEBNext Ultra II Directional RNA Library Prep Kit for Illumina (New England BioLabs). We also used an Agilent 4200 TapeStation (Agilent) to evaluate the quality and concentration of the libraries. Confirmed libraries were mixed in equal molar amounts for clustering and sequencing on an Illumina NovaSeq 6000 DNA sequencer with a 50-bp, using NovaSeq 6000 S1 Reagent Kit V1.5 (Illumina). We used CLC Genomics Workbench 23.0.2 for data analysis. Trimmed reads were mapped to the Mus musculus (GRCm39) release-110 database and counted according to default settings using a CLC Genomics Workbench (Qiagen). mRNA abundance was expressed as the log2-transformed transcripts per million plus one. We used the R package seqinr to read the FASTA file.

#### Immunoblotting

The cell lysates were extracted from the treated C2C12 myotubes by use of Lysis buffer containg M-PER Mammalian Protein Extraction Reagent (Thermo Fisher), EDTA-free Protease Inhibitor Cocktail (Roche) and Phosphatase Inhibitor Cocktail Solution (FUJIFILM Wako Pure Chemical Corporation) with sonication. Cultured cells were denatured with SDS sample buffer (Nacalai Tesque) at 99°C for 5 min. Lysates were loaded 10 μg protein/lane to run SDS-PAGE. The proteins were separated by SDS-PAGE and transferred to polyvinylidene fluoride (PVDF) membranes. The membranes were blocked with Block Ace (Bio-rad) and incubated at room temperature for 1 h with primary antibodies (Table S1). After washing PBS containing 0.1% Tween 20 (Sigma-Aldrich), the membranes were incubated at room temperature for 1 h with horseradish peroxidase (HRP)-conjugated anti-rabbit or anti-mouse IgG (Cell Signaling Technology) as secondary antibodies. Signals were developed using an Immobilon Western Chemiluminescent HRP Substrate (Cytiva) and examined using an Amasham imaging system (GE Healthcare). To analyze the intensity of bands, we used Fiji (ImageJ 1.53t).

#### Co-immunoprecipitation (Co-IP)

The cell lysates were extracted from the treated C2C12 myotubes by use of M-PER Mammalian Protein Extraction Reagent with sonication, and then cultured overnight with specific antibodies against p65 and IgG (negative control) in constant speed at 4°C. Following mixing with magnetic-beads (Cytiva), the antigen-antibody mixture was acquired. After washing thrice in PBS containing 0.1% Triton X-100 (Sigma-Aldrich), immunoblotting was conducted for the eluted proteins. See Table S1 for primer antibodies details.

#### Immunofluorescence

The cultured cells were fixed in pre-child 4% parahormardehid (PFA, FUJIFILM Wako Pure Chemical Corporation) at room tempreture for 10 min. After washing in PBS containing 0.1% Triton X-100 for 5 min, cells were incubated with 1% bovine serum albumin (BSA, Nacalai tesque) at room temperature for 60 min to block non-specific binding and then incubated with primary antibodies at room temperature for 1 h. Myogenic differentiation was evaluated by morphological assessment and by analysis of myogenic markers, including Myogenin (BD Pharmingen, 556358), MYH1 (Sigma-Aldrich, ZRB1214), MYH7 (Sigma-Aldrich, ZRB1308), Desmin (Proteintech, 16520-1-AP), and PAX7 (abcam, ab324017). After washing with PBS, the cells and sections were incubated with an Alexa Fluor 488 Goat Anti-Rabbit IgG H&L (abcam) as a secondary antibody at room temperature for 1 h the cells and sections were enclosed using ProLong Glass Antifade Mountant (Thermo Fisher Scientific) after staining the nuclei with 4’,6-diamidino-2-phenylindole (DAPI, Dojindo) at room temperature for 5 min. The fluorescent images were obtained under a fluorescence microscope (Keyence). See Table S1 for primer antibodies details.

The fusion index, number of myonuclei, and percentage of PAX7-positive cells were quantified using Fiji. The fusion index was calculated as the percentage of nuclei located within MyHC-positive multinucleated myotubes relative to the total number of DAPI-positive nuclei. Myotubes were defined as MyHC-positive cells containing at least two nuclei. The number of myonuclei per myotube was determined by counting DAPI-positive nuclei within individual MyHC-positive multinucleated myotubes. The percentage of PAX7-positive cells was calculated as the number of PAX7-positive nuclei relative to the total number of DAPI-positive nuclei.

#### Cell proliferation assay

C2C12 or murin primary myoblasts were seeded in 96-well plates at 5,000 cells/well with 1mM 4-PBA or DMSO. After incubated for 24, 48, and 72 h, cell proliferation was measured using CellTiter 96 AQ_ueous_ One Solution Cell Proliferation Assay (Promega) according to the manufacturer’s instructions. We measured the absorbance at 490 nm using a microplate reader (FlexStation3, Molecular Devices).

#### Quantitative real time Polymerase Chain Reaction (qPCR)

Total RNA was prepared using the ISOSPIN Cell & Tissue RNA (Nippon Gene) and was transcribed into cDNA using the ReverTra Ace qPCR RT Master Mix with gRNA remover (Toyobo). The qPCR was performed using PowerTrack SYBR Green Master Mix for qPCR (Thermo Fisher Scientific) with specific forward and reverse primers in a QuantStudio3 (Thermo Fisher Scientific). The relative gene expression was calculated with the ΔΔCt method,^69^ using GAPDH or Actin for normalization. See Table S2 for qPCR primer details.

#### Chromatin immunoprecipitation (ChIP) - qPCR

ChIP DNA was obtained using The Simple ChIP Enzymatic Chromatin IP Kit (Cell Signaling Technology) according to the manufacturer’s instructions. Briefly, 4 × 10^6^ C2C12 cells were treated with 37% PFA (FUJIFILM Wako Pure Chemical Corporation) for 15 min for cross-link, and then with sonication for shearing DNA into 500-bp of fragments. The immunoprecipitation was implemented with 30 μL magnetic beads for 2 h, following overnight incubation of anti-p65 or control IgG antibodies. The collected precipitated chromatin was subjected to qPCR. See Table S3 for ChIP-qPCR for primer details.

#### JC-1 mitochondrial membrane potential assay

Mitochondrial membrane potential was assessed using a JC-1 MitoMP Detection Kit (Dojindo). Cells were seeded in culture plates and treated under the indicated experimental conditions. After treatment, cells were incubated with JC-1 staining solution according to the manufacturer’s instructions. After staining, cells were washed to remove excess dye with Hanks’ Balanced Salt Solution (Nacalai Tesque), and fluorescence intensity was measured using a microplate reader. JC-1 monomer fluorescence was detected at an excitation wavelength of 485 nm and an emission wavelength of 545 nm, whereas JC-1 aggregate fluorescence was detected at an excitation wavelength of 535 nm and an emission wavelength of 605 nm. Mitochondrial membrane potential was evaluated by calculating the ratio of JC-1 aggregate fluorescence to JC-1 monomer fluorescence.

### Quantification and statistical analysis

Statistical analysis were performed using R software (v4.5.1). We used Student’s *t* test to compare differences between two samples. We also used one-way ANOVA and Tukey-Kramer test for differences between the variances of the three groups. For C2C12 experiments, n represents independent experiments, whereas for primary murine myoblast experiments, n represents independent mice. Values are presented as means ± SEM.

Visualized and analyses were performed using the following packages: clusterProfiler (version 4.16.0), KEGGREST (version 1.48.1), and GO.db (version 3.21.0), ggplot2 (version 4.0.0), ggpubr (version 0.6.1), pheatmap (version 1.0.13), VennDiagram (version 1.7.3), org.Mm.e.g.,.db (version 3.21.0) from Bioconductor.^70^

Gene Ontology (GO) enrichment analysis was performed using GO annotations.^71^ Biological process terms were tested for enrichment using a hypergeometric test, and *p*-values were adjusted for multiple testing using the Benjamini-Hochberg method. GO terms with an adjusted *p*-value <0.05 were considered significant. KEGG pathway enrichment analysis was evaluated using a hypergeometric test with all expressed genes as the background gene set.^72^ *p*-values were adjusted using the Benjamini-Hochberg method, and adjusted *p*-values <0.05 were considered significant.

## Supplemental information

Document S1. Figures S1–S10 and Tables S1–S3

Data S1. Uncropped western blot images corresponding to the immunoblotting experiments presented in this study
