# Supplementary material for: 4-Phenylbutyric acid activates an NF-κB-Egr-1 axis to control myoblast proliferation and ECM gene expression
Source: iScience. 2026 Sep 24;29(10):117620. doi: 10.1016/j.isci.2026.117620 (PMC13634890; doi:10.1016/j.isci.2026.117620)
Supplement: Supplementary file 1 [file mmc1.pdf]

iScience, Volume *29*

Supplemental information

4-Phenylbutyric acid activates an NF-κB-Egr-1 axis to control myoblast proliferation and ECM gene expression

Kana Tominaga and Naoomi Tominaga

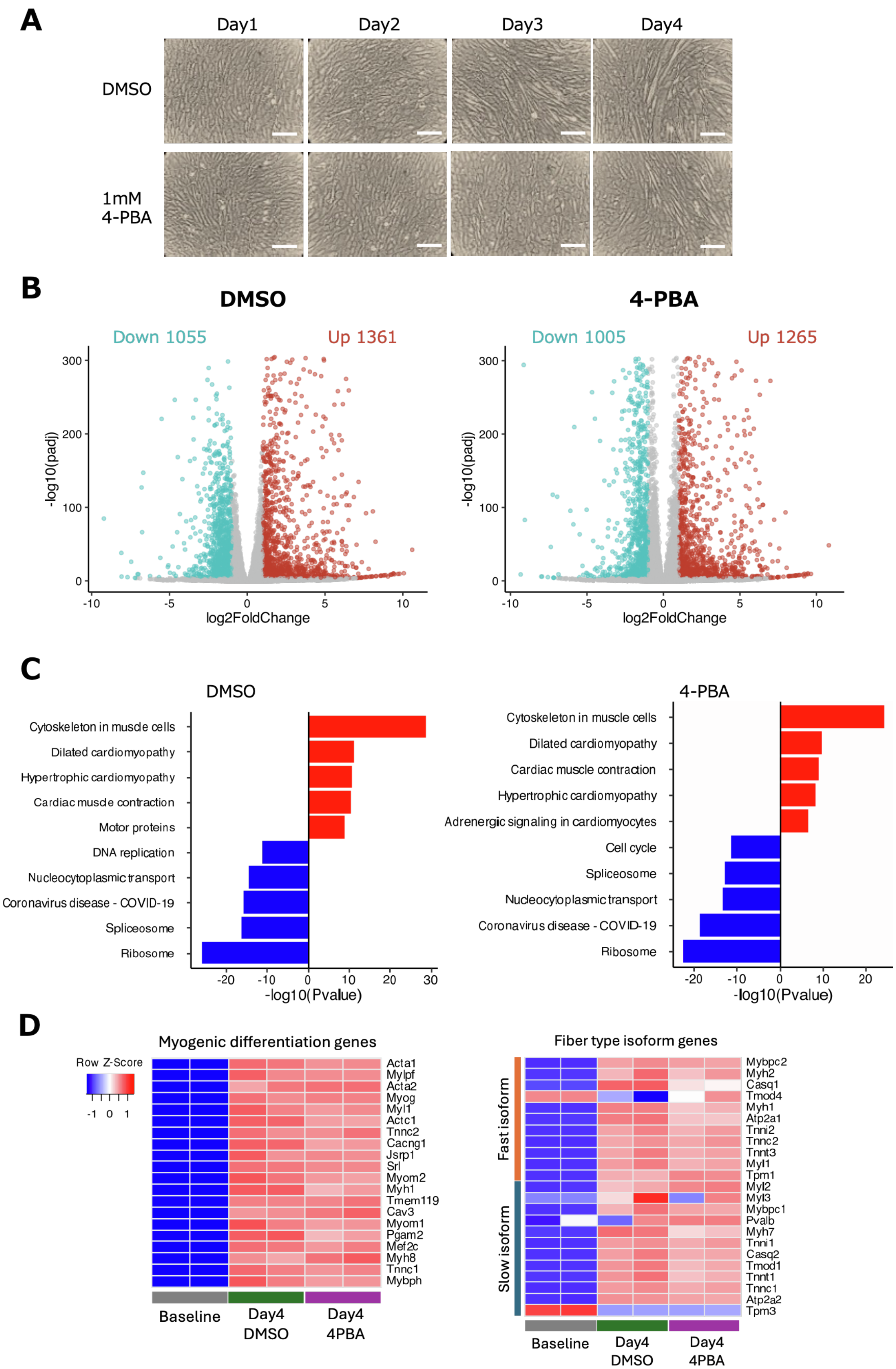
A
Day1
Day2
Day3
Day4
DMSO
1mM 4-PBA
B
DMSO
4-PBA
Down 1055
Up 1361
Down 1005
Up 1265
-log10(padj)
log2FoldChange
C
DMSO
4-PBA
Cytoskeleton in muscle cells
Dilated cardiomyopathy
Hypertrophic cardiomyopathy
Cardiac muscle contraction
Motor proteins
DNA replication
Nucleocytoplasmic transport
Coronavirus disease - COVID-19
Spliceosome
Ribosome
Adrenergic signaling in cardiomyocytes
Cell cycle
-log10(Pvalue)
D
Myogenic differentiation genes
Row Z-Score
Acta1
Mylpf
Acta2
Myog
Myl1
Actc1
Tnnc2
Cacng1
Jsrp1
Srl
Myom2
Myh1
Tmem119
Cav3
Myom1
Pgam2
Mef2c
Myh8
Tnnc1
Mybph
Baseline
Day4 DMSO
Day4 4PBA
Fiber type isoform genes
Fast isoform
Slow isoform
Mybpc2
Myh2
Casq1
Tmod4
Myh1
Atp2a1
Tnni2
Tnnc2
Tnnt3
Myl1
Tpm1
Myl2
Myl3
Mybpc1
Pvalb
Myh7
Tnni1
Casq2
Tmod1
Tnnt1
Tnnc1
Atp2a2
Tpm3

Figure S1. Effects of 4-Phenylbutyric Acid on Myogenic Differentiation and Gene Expression in C2C12 Cells, Related to Figure 1.

(A) Representative phase-contrast images of C2C12 myoblasts treated with 1 mM 4-phenylbutyric acid (4-PBA) or DMSO under differentiation conditions. Scale bar, 400 μm.

(B) Volcano plot of differentially expressed genes identified by RNA-seq analysis, comparing 4-PBA- and DMSO-treated C2C12 cells before treatment (baseline) and at day 4 after myotube differentiation. Significantly upregulated genes are shown in red and downregulated are shown in blue. FC, fold change; P-adj, adjusted p value.

(C) KEGG pathway enrichment analysis of differentially expressed genes in 4-PBA- and DMSO-treated C2C12 myotubes, comparing with baseline and day 4 after myotube differentiation.

(D) Heatmap showing RNA-seq-based expression profiles of myogenic differentiation genes in C2C12 cells treated with 1 mM 4-PBA or DMSO at day 4 after myotube differentiation and at baseline (Day 0).

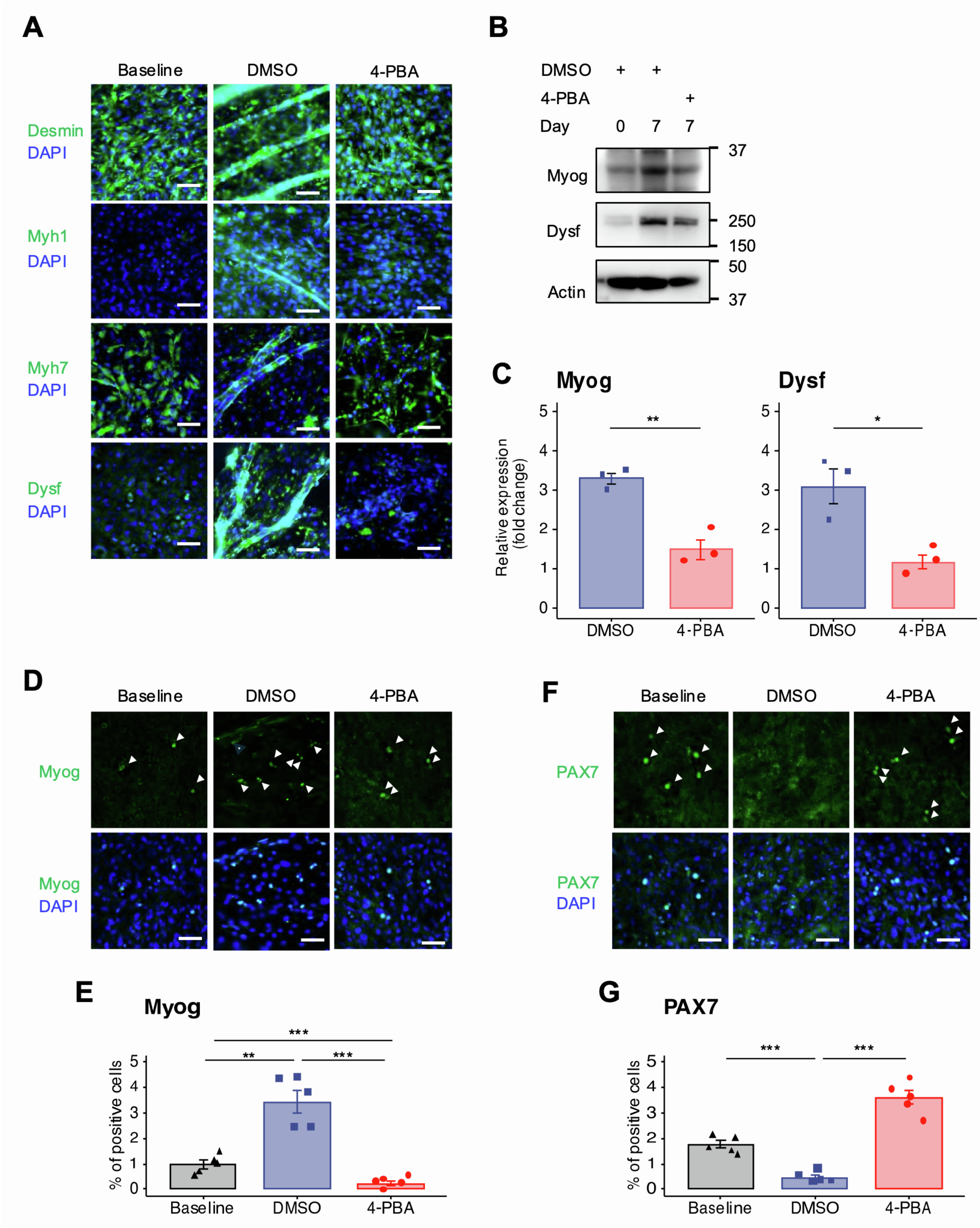

Figure S2. Extended Differentiation Analysis of 4-PBA-treated cells, Related to Figure 1.

(A) Representative immunofluorescence images of C2C12 myotubes stained for myogenic markers, including Desmin, Myosin Heavy Chain 1 (Myh1), Myosin Heavy Chain 7 (Myh7), and Dysferlin (Dysf). Myogenic markers are shown in green, and nuclei are counterstained in blue. Scale bar, 50 µm.

(B) Representative immunoblots showing Myogenin (Myog) and Dysf expression in C2C12 cells treated with 1 mM 4-PBA or DMSO for 7 days.

(C) Quantification of Myog and Dysf protein levels shown in (B), normalized to β-actin (Actin). Day 7 values are presented as fold change relative to Day 0 (Baseline). Data are representative of three independent experiments.

(D) Representative immunofluorescence images of C2C12 myotubes stained for Myog. Myog is shown in green, and nuclei are counterstained in blue. White arrowheads indicate Myog-positive cells. Scale bar, 50 µm.

(E) The percentage of Myog-positive cells was calculated by normalizing the number of marker-positive cells to the total number of nuclei. Data are represented five independent experiments.

(F) Representative immunofluorescence images of C2C12 myotubes stained for PAX7. PAX7 is shown in green, and nuclei are counterstained in blue. White arrowheads indicate PAX7-positive cells. Scale bar, 50 µm.

(G) The percentage of PAX7-positive cells was calculated by normalizing the number of marker-positive cells to the total number of nuclei. Data are represented five independent experiments.

Data in (C), (E) and (G) are presented as mean ± SEM. and analyzed using an unpaired two-tailed Student's t test (C) and one-way ANOVA with Tukey's post hoc test (E, G). Each dot represents one independent experiment. *$p < 0.05$, **$p < 0.01$, ***$p < 0.001$.

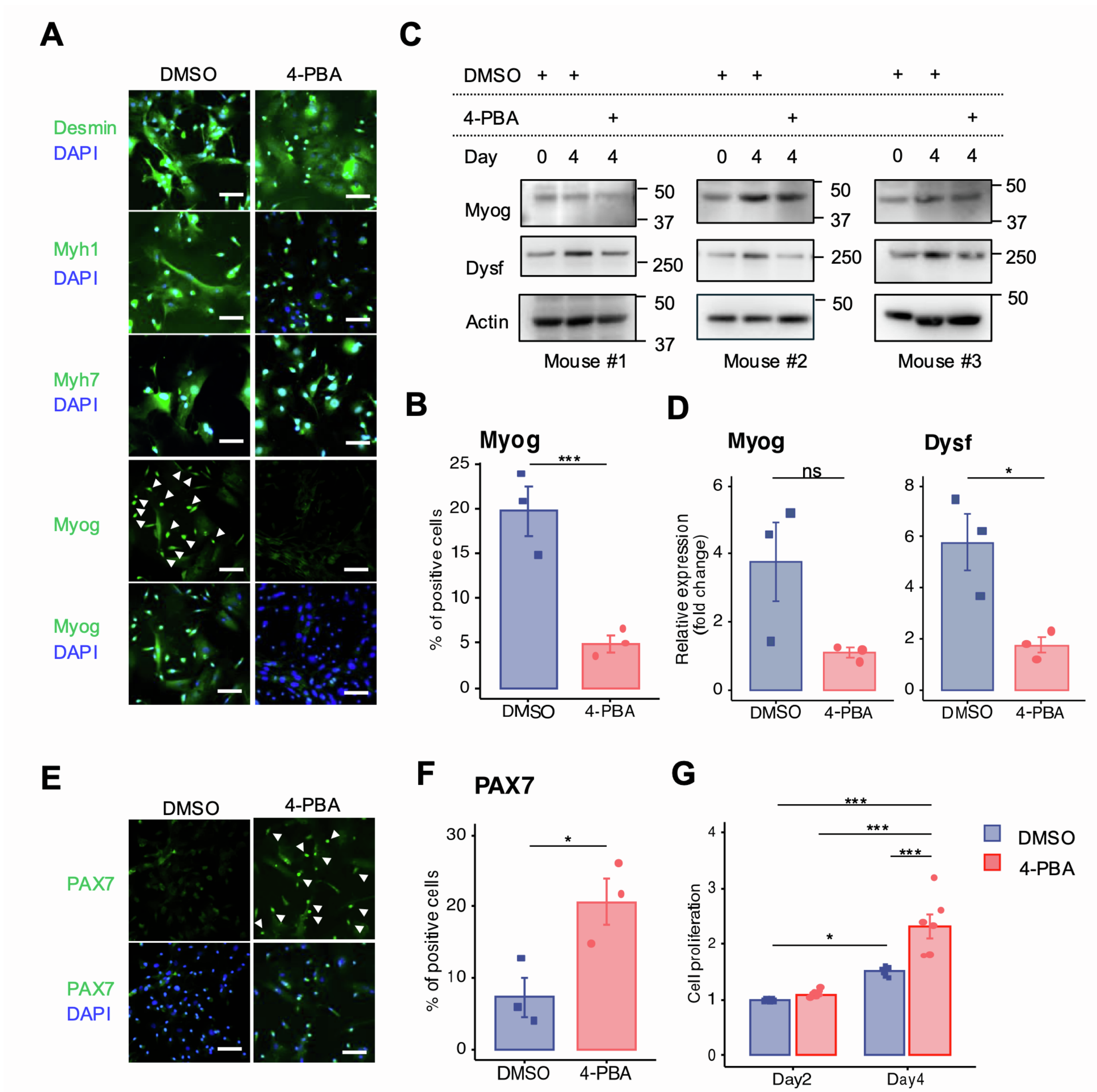

Figure S3. Primary Myogenic Cell Validation of 4-PBA Effects, Related to Figure 1.

(A) Representative immunofluorescence images of primary murine myotubes stained for myogenic markers, including Desmin, Myosin Heavy Chain 1 (Myh1), Myosin Heavy Chain 7 (Myh7) and Myogenin (Myog). Myogenic markers are shown in green, and nuclei are counterstained in blue. White arrowheads indicate Myog-positive cells. Scale bar, 50 µm.

(B) The percentage of Myog-positive cells was calculated by normalizing the total number of nuclei. Data are representative of three independent primary murine myotubes (5 fields per mouse).

(C) Immunoblots showing Myog and Dysferlin (Dysf) expression in three independent primary murine myotubes treated with 1 mM 4-PBA or DMSO for 7 days. β-actin (Actin) was used as a loading control.

(D) Quantification of Myog and Dysf protein levels shown in (C), normalized to Actin. Values are presented as fold change relative to Day 0 (Baseline). Data are representative of three independent primary murine myotubes.

(E) Representative immunofluorescence images of primary murine myotubes stained for PAX7. PAX7 is shown in green, and nuclei are counterstained in blue. White arrowheads indicate PAX7-positive cells. Scale bar, 50 μm.

(F) The percentage of PAX7-positive cells was calculated by normalizing the number of marker-positive cells to the total number of nuclei. Data are representative of three independent primary murine (5 fields per mouse).

(G) Cell proliferation of three independent primary murine myoblasts treated with 1 mM 4-PBA or DMSO for the indicated time points. Values are presented as fold change relative to Day 0 (Baseline). Data are representative of two independent primary murine (3 wells per condition).

Data in (B), (D), (F) and (G) are presented as mean ± SEM. and analyzed using an unpaired two-tailed Student's t test (B, D, F) and one-way ANOVA with Tukey's post hoc test (G). Each dot represents one independent experiment. *$p < 0.05$, **$p < 0.01$, ***$p < 0.001$; ns, not significant.

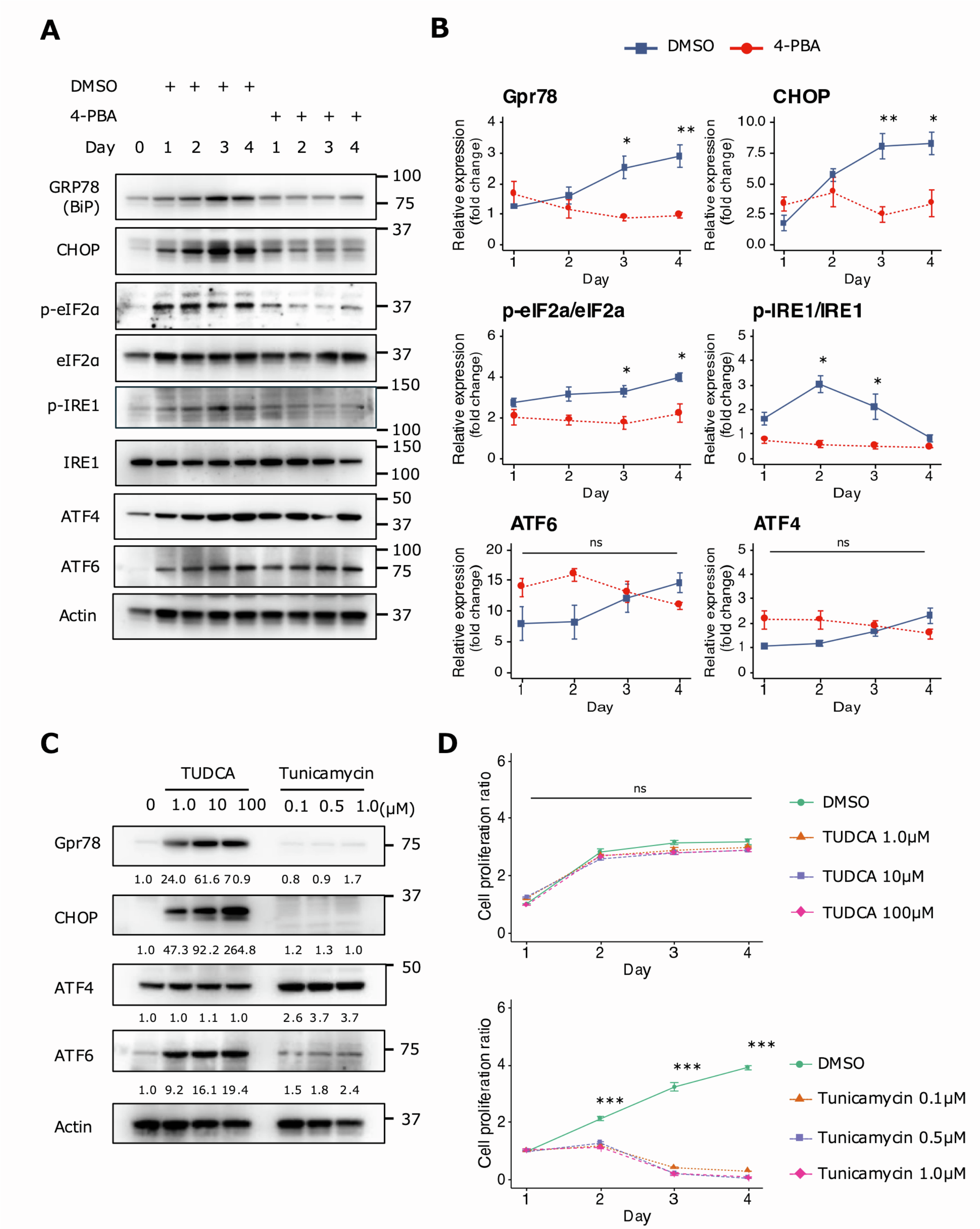

Figure S4. ER Stress Modulation by 4-PBA and Effects of ER Stress Modulators on Myoblast Proliferation, Related to Figures 2.

(A) C2C12 myoblasts were treated with 4-PBA (1mM) or DMSO as a control for indicated time point. Representative immunoblots showing ER stress markers, GPR78 (BiP), CHOP, phosphorylation of eIF2α (Ser51), total eIF2α, phosphorylation of IRE1 (Ser724), and total IRE1, ATF4, ATF6 and to β-actin (Actin, loading control).

(B) Quantification of ER stress markers shown in (A). Band intensities were normalized to Actin, total eIF2α, or total IRE1, respectively. Data represent three independent experiments.

(C) C2C12 myoblasts were treated with indicated concentration of TUDCA or Tunicamycin for 24 hours, after which total cell lysates were prepared and subjected to immunoblot analysis with antibodies to ER stress markers, GPR78 (BiP), CHOP, ATF4 and ATF6 and to Actin as a loading control. ER stress marker levels were normalized to Actin band intensity. Data are from a representative experiment.

(D) Proliferation of C2C12 myoblasts treated with indicated concentration of TUDCA or Tunicamycin for the indicated time points. Values are presented as fold change relative to Day 0 (Baseline). Data are represented four independent experiments.

Data in (B) and (D) are presented as mean ± SEM. and analyzed using an unpaired two-tailed Student's t test (B) or one-way ANOVA with Tukey's post hoc test (D). *$p < 0.05$, **$p < 0.01$, ***$p < 0.001$ vs DMSO-treated cells (Control); ns, not significant.

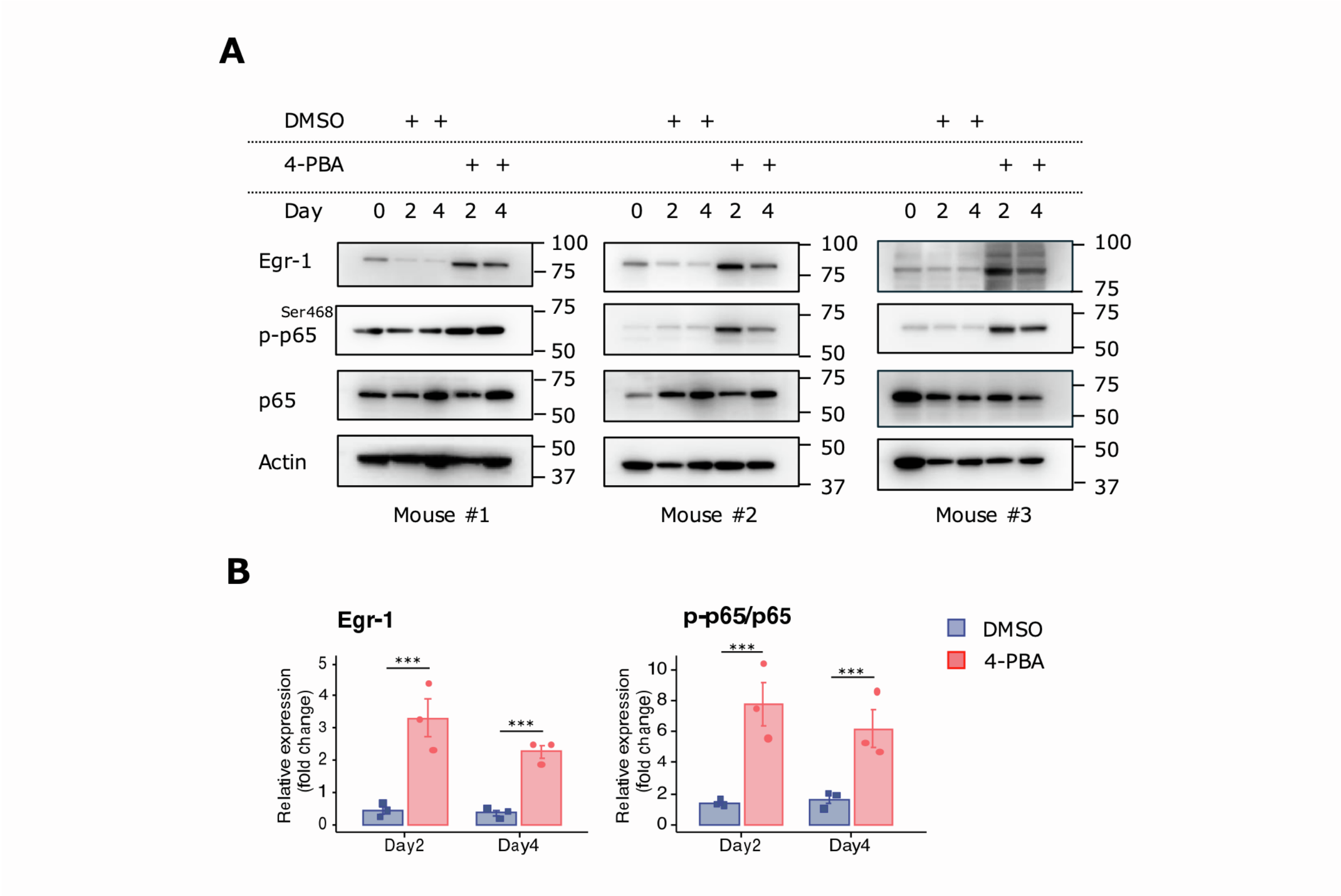

Figure S5. Primary Myogenic Cell Validation of 4-PBA Effects, Related to Figure 2 and 3.

(A) Representative immunoblots showing Egr-1, phosphorylated p65 (Ser468) and total p65 protein expression in three independent primary murine myoblasts treated with 1 mM 4-PBA or DMSO for the indicated time points. β-actin (Actin) was used as a loading control.

(B) Quantification of Egr-1 shown in (A). Band intensities were normalized to Actin. Data represent three independent primary murine myotubes. Values are presented as fold change relative to Day 0 (Baseline).

(C) Quantification of phosphorylated p65 (Ser468) shown in (A). Band intensities were normalized to total p65. Data represent three independent primary murine myotubes. Values are presented as fold change relative to Day 0 (Baseline).

Data in (B) are presented as mean ± SEM. from three independent experiments and analyzed using an unpaired two-tailed Student's t test. Each dot represents one independent mouse. *$p < 0.05$, **$p < 0.01$, ***$p < 0.001$.

A

sequence	start ~ end	strand
CTGGAATCCC	-162 ~ -171	+
GGGAACTCCA	-1418 ~ -1427	-

B

Mouse Gene Egr-1 (ENSMUST00000064795.6)

TGGCCTGTTTGGGTCATGTGCCCATCCAAAAGTCATCACTGCAGGAAACAGAATGCTCATGGCTTACAAGGTCC

TATATGAATGGCCAGGCCTGGGTCACAGACCTACTCTTAGATTTTGGTGTGGAGTTCCCACAATCTCAGGAGTG

ACAGTAGCACCTCAGAAGAAATTGCTTACTGGGGAATATTCCAAAACTAACAGGTGCTGGGTACACAGAAGATA

CACACATGCAAATACCTCAGGCAGACTTGCACATCAGAGTCATGGGGGGAAATGAATATGATATTTAGAACTCT

ACTAGTTCTTAATACGACTTAGCCAGGGAGACAGAGACCTATGGGTTTATTTGAACCACTTTAGCACAATTACCA

GGCCATTACCCCTAAACTATCCCACACTCAAGTCACCAGCCAATCAGTTGTCCTGTCTGTGCTGGATCCACTCC

AGCCATGCCCTGCTTCTCCCCCCTCTTTCTCTTCCATGTGCCCCCTACCTACAGACCCTTCCCACCTCCCTACCT

CTGAAGCCCAGCCTACTTTTATTCTGCTCAGATCAGCCTCAGCCATCAGCAACTTTTTTTTTTTTAAGATTTATTTA

TTTTATGTATATGAGTACACTGTAGCTGTACAGATGGTTGTGAGCCATCATGTGGATTCTGGGAATTGAATTCAG

GACCTCTGCTCACTCCAGTTGGTCCTGCTCGCTCTGGCCCAAAGATTTATTTATTATTATTATCTAAGTACACTGT

AGCTGTCTTCAGACTCACCAGAAGAATGTGTCAGATCTCATTACAGATGGCTGTGAGCCACCATGTGGTTGTGG

GATTTGAACTCAGGATCTTTGGAAGAACAGTCAGTGCTCTTAACCGCTGAGCTATCTCTCCAGCTCCAATACTGT

CTTTTCAGTCCCCCACCGCGTGTCTCCAGTGCTTAGTAACGGGCAGTACGCGCTCTACATTTGGTAGTCACAGG

TCTGAACAAACAAACAATCCTTCCTCATGAAAACAGAGTTTACAACAAGAGCAGGTGAGACACAGGTTTGGTCA

GAGAATCCCACCAGAGTATCAAAATGGGCATGCTCTCACAGGAAGCACCTGGCCCTGCCTGTCCTGGCCAGGA

GGCTATGGAAAGGCACATTAATGGCTCCTAAATTAGTAACAACCAAAGAACTCAAACAGAATGGAACCCTGGTT

CATGTCTGGATTCCTTAAGCTGTTTTGGTTTTTTTAAATAAATAAAACGAAGGTGGAGGCGGATGGAGAGAGAGA

GAGAGAGAGAGAGAGAGAGAGAGAGAGAGAGAGAGAGAGAGCCGAAGAGTAACCTCCCTGTTAACCTTGACGTC

P2_Forward

CCCGATCTTCCTTCTTCTGTCTCTCAATCTCCTTCCACACAGGCACTCTCTGCTTTCTTTTTAAATAAAAAAATAAA

P1_Forward *P2_Reverse*

ATTAAAATAACAGCACCTTCCTCGTATTCAAAGTTGGAAACAAGAGCCTCCCATTCCTGGAATCCCTTCTCCCTT

P1_Reverse *P3_Forward*

TGGGTTGCTTCGGAGATAGGGCTTCACTGCTTGCGTCAGGGTCCCGGGAGACCAGCGGGATCTCTCTGCCATC

P3_Reverse

ACACCCCCGCCCCCTCCCCCCCCCCCCTGTTCCCTGCCCTTGGCCTGGCTCTGTGAAGGAAGTGTTACCCTG

AATTCTGGGCGCTTTGGCAGTGGCGGTTCCCTCGGGACTGCGGGGAAGGCCCAGGCCGCCG

Figure S6. Identification of a Putative NF-κB p65 Binding Site in the Murine *Egr-1* Promoter, Related to Figure 3.

(A) Predicted NF-κB p65 binding site in the −2000 bp upstream region of the murine *Egr-1* promoter.

(B) Structure of the murine *Egr-1* promoter and primers (P1, P2 and P3). Predicted NF-κB p65 binding site is shown in blue. The T in red represents +1 site.

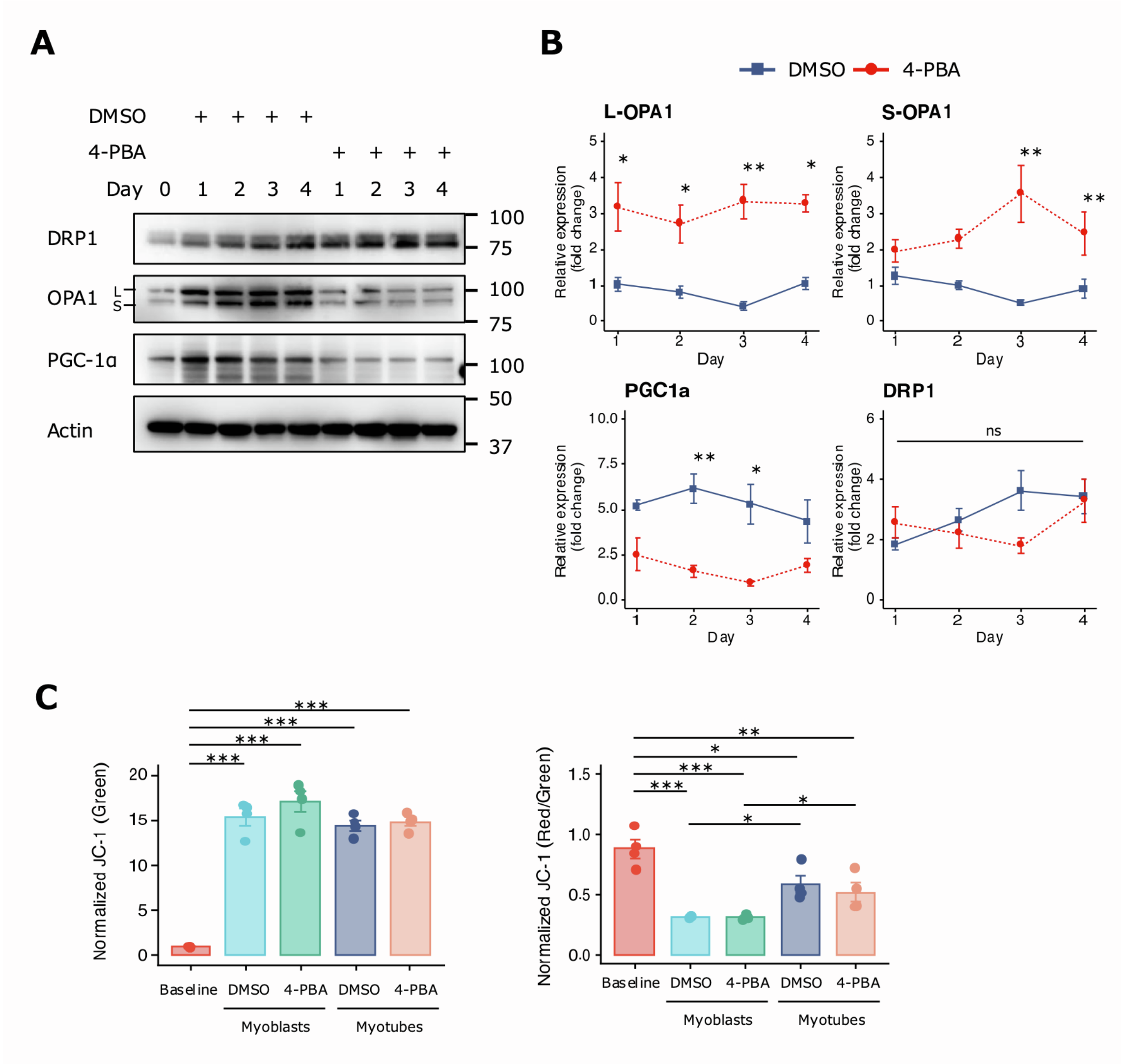

Figure S7. Mitochondrial Membrane Potential Analysis after 4-PBA Treatment in Myoblasts and Myotubes, Related to Figure 3.

(A) C2C12 myoblasts were treated with 4-PBA (1mM) or DMSO as a control for indicated time point. Representative immunoblots showing mitochondrial membrane markers, DRP1, OPA1, PGC1α, and β-actin (Actin) as a loading control. OPA1 expression showing total OPA1 (L-OPA1, 90 kDa) and cleavage products, S-OPA1 (75-86 kDa).

(B) Quantification of mitochondrial membrane markers shown in (A). Band intensities were normalized to Actin. Data represent three independent experiments.

(C) JC-1 mitochondrial membrane potential assay. Analysis of red (excitation wavelength of 535 nm, emission wavelength of 605 nm) to green (excitation wavelength of 485 nm, emission wavelength of 545 nm) fluorescence intensity of C2C12 myoblasts or myotubes were treated with 4-PBA (1mM) or DMSO as a control for 2 days. Data represent three independent experiments. Data represent four independent experiments.

Data in (B and C) are presented as mean ± SEM. and analyzed using an unpaired two-tailed Student's t test (B) or one-way ANOVA with Tukey's post hoc test (C). *p < 0.05, **p < 0.01, ***p < 0.001; ns, not significant.

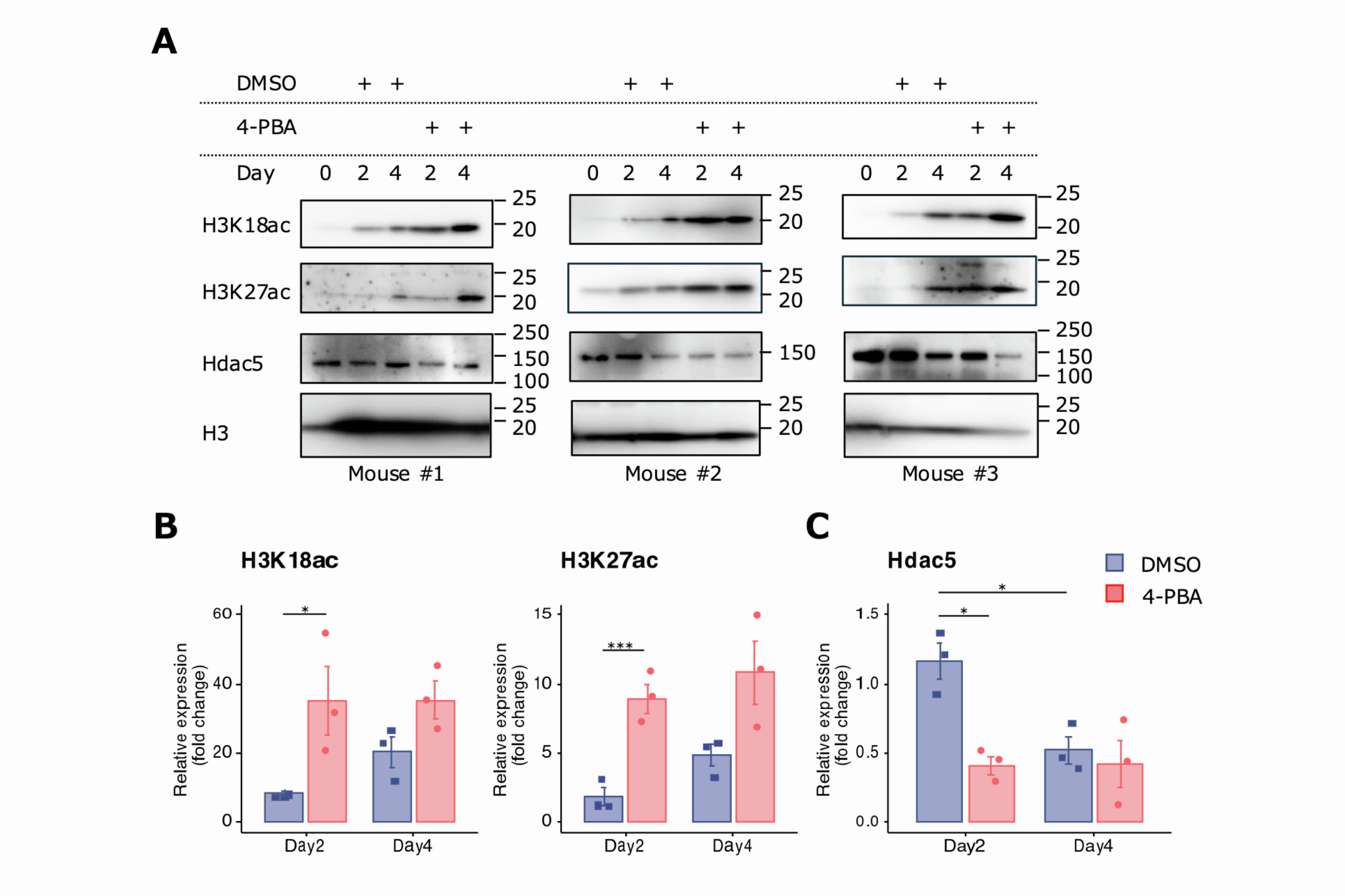

Figure S8. Primary Myogenic Cell Validation of 4-PBA Effects, Related to Figure 4 and 5.

(A) Immunoblots showing HdacH3K18ac, H3K27ac and Hdac5 expression in three independent primary murine myoblasts treated with 1 mM 4-PBA or DMSO for the indicated time points. Histon H3 (H3) was used as a loading control.

(B) Quantification H3K18ac and H3K27ac protein levels shown in (A). Band intensities were normalized to total Histon H3. Values are presented as fold change relative to Day 0 (Baseline).

(C) Quantification Hdac5 protein levels shown in (A), normalized to Histon H3. Band intensities were normalized to total Histon H3. Values are presented as fold change relative to Day 0 (Baseline).

Data in (B and C) are presented as mean ± SEM. and analyzed using a one-way ANOVA with Tukey's post hoc test. Each dot represents one independent mouse. *$p < 0.05$, **$p < 0.01$, ***$p < 0.001$.

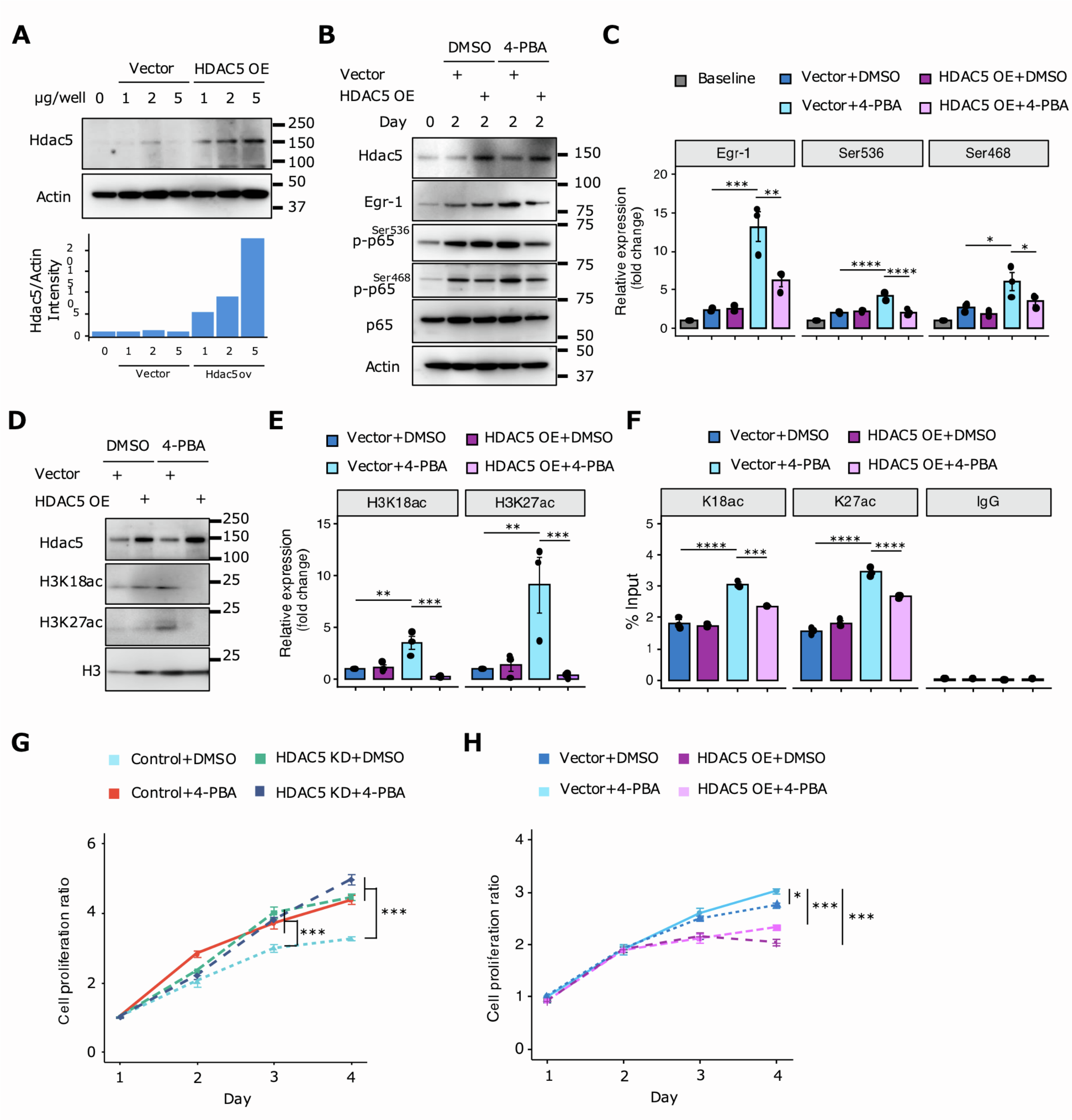

Figure S9. HDAC5 regulates 4-PBA-induced NF-κB/Egr-1 activation, histone H3 acetylation, and myoblast proliferation., Related to Figure 5.

(A) Optimization of transient Hdac5 overexpression in C2C12 cells. Cells were transfected with empty vector (Vector) or Hdac5 expression vector (HDAC5 OE) at the indicated amounts (1, 2, or 5 μg/well), and HDAC5 protein expression was examined by immunoblotting. Bottom: Quantification of HDAC5 protein levels normalized to Actin. Data are from a representative experiment.

(B) Representative immunoblots showing the effects of transient HDAC5 overexpression on 4-PBA-induced NF-κB/Egr-1 signaling. C2C12 cells were transfected with empty vector (Vector) or Hdac5 expression vector (HDAC5 OE, 5 μg) and then treated with vehicle control or 1 mM 4-PBA during

myogenic differentiation. Cells were collected at differentiation Day 2. Egr-1, total p65, phosphorylated p65 at Ser536, and phosphorylated p65 at Ser468 were examined by immunoblotting.

(C) Quantification of the immunoblot data shown in (B). Egr-1 was normalized to Actin, and phosphorylated p65 levels were normalized to total p65. Data represent three independent experiments.

(D) Representative immunoblots showing the effects of transient HDAC5 overexpression on histone H3 acetylation. C2C12 cells were transfected with empty vector or Hdac5 expression vector (5 μg) and then treated with vehicle control or 1 mM 4-PBA during myogenic differentiation. Cells were collected at differentiation Day 2. H3K18ac, H3K27ac, and total histone H3 were examined by immunoblotting.

(E) Quantification of the immunoblot data shown in (D). H3K18ac and H3K27ac levels were normalized to total histone H3. Data represent three independent experiments.

(F) ChIP-qPCR analysis of H3K18ac and H3K27ac enrichment at the Egr-1 promoter (P1). C2C12 cells were transfected with empty vector or Hdac5 expression vector (5 μg) and then treated with vehicle control or 1 mM 4-PBA during myogenic differentiation. Cells were collected at differentiation Day 2. Enrichment at the Egr-1 promoter region was analyzed and is presented relative to input. Data represent three independent experiments.

(G) Cell proliferation assay of C2C12 myoblasts transfected with control siRNA (Control) or Hdac5 siRNA (HDAC5 KD) and treated with vehicle control or 1 mM 4-PBA. Data represent four independent experiments.

(H) Cell proliferation assay of C2C12 cells transfected with empty vector or Hdac5 expression vector (5 μg) and treated with vehicle control or 1 mM 4-PBA. Values are presented as fold change relative to Day 0. Data represent four independent experiments.

Data (C, E, F, G and H) are presented as the mean ± SEM. and analyzed using one-way ANOVA with Tukey's post hoc test. Each dot represents one independent experiment. *$p < 0.05$, **$p < 0.01$, ***$p < 0.001$ vs DMSO-treated cells (Control).

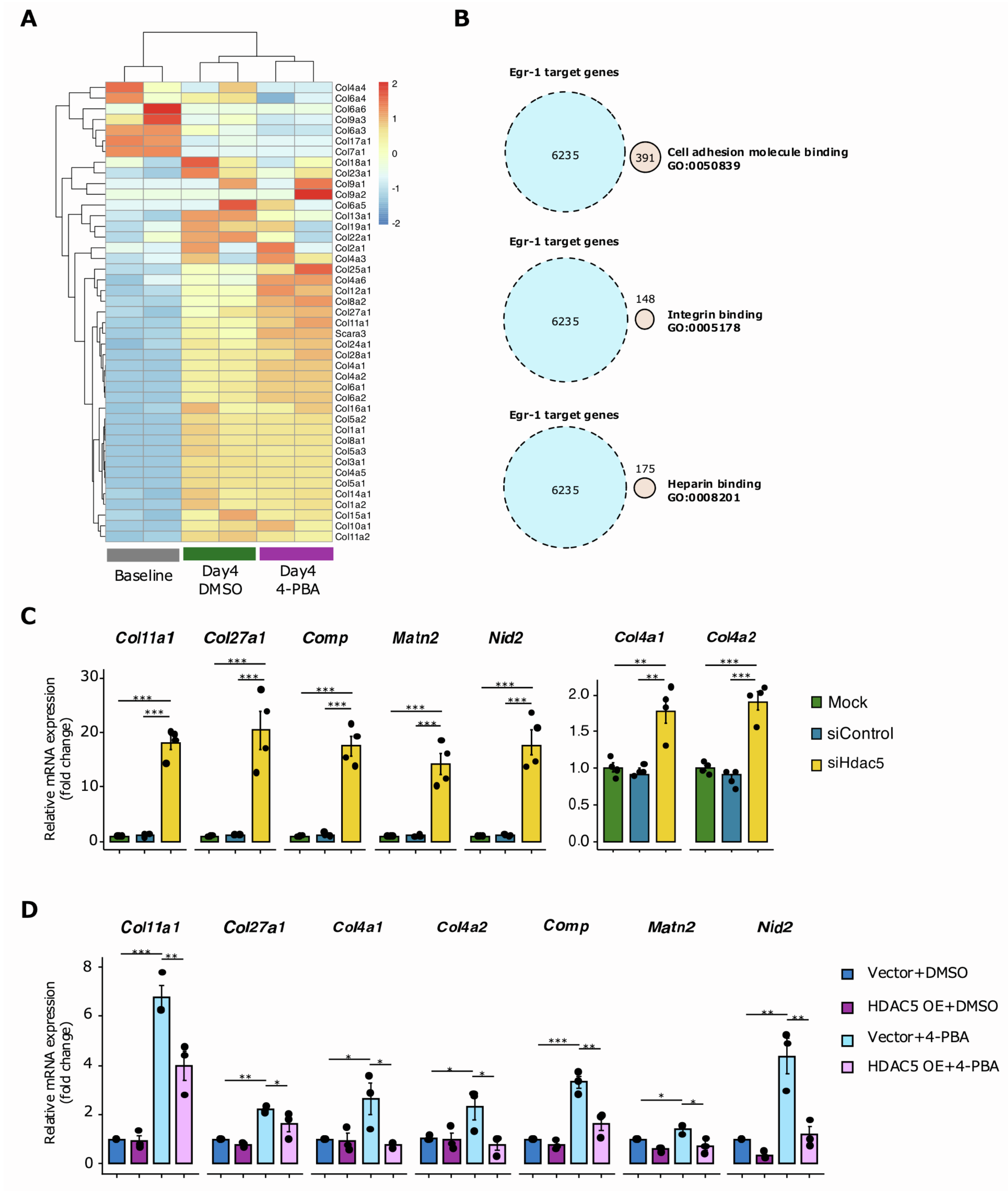

Figure S10. HDAC5–Egr-1 Axis Controls Expression of Collagen and Adhesion-Related Genes in C2C12 Cells, Related to Figure 6.

(A) Heatmap showing RNA-seq-based expression profiles of collagen genes in C2C12 cells treated with 1 mM 4-PBA or DMSO at day 4 after myotube differentiation and at baseline (Day 0).

(B) Venn diagram showing overlap between genes associated with the the adhesion molecule binding (GO:0050839), integrin binding (GO:0005178), and heparin binding (GO:0008201) and predicted Egr-1 target genes obtained from the ENCODE Transcription Factor Targets Dataset.

(C) Relative mRNA expression of selected ECM-related genes in C2C12 cells transfected with siHdac5 (15 nM), siControl, or mock control, showing Egr-1–dependent transcriptional regulation. Expression levels were normalized to β-actin (Actin). Data represent four independent experiments.

(D) Relative mRNA expression of selected ECM-related genes in C2C12 cells transfected with empty vector (Vector) or Hdac5 expression vector (HDAC5 OE, 5 µg), showing Egr-1–dependent transcriptional regulation. Expression levels were normalized to β-actin (Actin). Data represent three independent experiments.

Data in (C and D) are presented as mean ± SEM. and analyzed using a one-way ANOVA with Tukey's post hoc test. Each dot represents one independent experiment. *$p < 0.05$, **$p < 0.01$, ***$p < 0.001$.

Table S1. List of antibodies used in this study.

Antibody	Reference	Use and Dilution
Egr-1	Proteintech,55117-1-AP	Western blotting (1:1000)
Fos	Proteintech, 66590-1-IG	Western blotting (1:500)
Myh1	Sigma-Aldrich, ZRB1214	Immunofluorescence (1:100)
Myh7	Sigma-Aldrich, ZRB1308	Immunofluorescence (1:100)
Dysferlin	Leica Biosystems, NCL-Hamlet	Western blotting (1:1000)<br>Immunofluorescence (1:100)
Myogenin	BD Biosciences, 556358	Western blotting (1:1000)<br>Immunofluorescence (1:100)
β-Actin	GeneTex, GTX110564	Western blotting (1:10000)
Phospho-NF-κB p65 (Ser468)	Proteintech, 82335-1-RR	Western blotting (1:500)
Phospho-NF-kB p65 (Ser536)	Cell Signaling Technology, #3033	Western blotting (1:1000)
Acetyl-NFkB p65 (Lys310)	Invitrogen, PA5-17264	Western blotting (1:1000)
NF-κB p65	Biolegend, 622602	Western blotting (1:1000)<br>Immunoprecipitation (1:50)
Histone H3 (D1H2)	Cell Signaling Technology, #4499	Western blotting (1:1000)
Acetyl-Histone H3 (Lys9)	Cell Signaling Technology, #9649	Western blotting (1:1000)
Acetyl-Histone H3 (Lys14)	Cell Signaling Technology, #7627	Western blotting (1:1000)
Acetyl-Histone H3 (Lys18)	Cell Signaling Technology, #13998	Western blotting (1:1000)
Acetyl-Histone H3 (Lys27)	Cell Signaling Technology, #8173	Western blotting (1:1000)
Acetyl-Histone H3 (Lys56)	Cell Signaling Technology, #4243	Western blotting (1:1000)
HDAC5	Cell Signaling Technology, #20458	Western blotting (1:1000)
Desmin	Proteintech, 16520-1-AP	Immunofluorescence (1:400)
Pax7	abcam, ab324017	Immunofluorescence (1:500)
OPA1	Cell Signaling Technology, #80471	Western blotting (1:1000)
DRP1	Proteintech, 12957-1-AP	Western blotting (1:5000)

PGC1a	Proteintech, 66369-1-IG	Western blotting (1:10000)
Phospho-eIF2α (Ser51)	Cell Signaling Technology, #3398	Western blotting (1:1000)
eIF2α (D7D3)	Cell Signaling Technology, # 5324	Western blotting (1:1000)
Grp78	GeneTex, GTX113340	Western blotting (1:10000)
ATF4	GeneTex, GTX101943	Western blotting (1:1000)
Phospho-IREa (Ser724)	GeneTex, GTX132808	Western blotting (1:1000)
IRE1	Cell Signaling Technology, #3294	Western blotting (1:1000)
ATF6	Novus Biologicals, NBP1-40256	Western blotting (1:250)

Table S2. List of primers used for quantitative RT-PCR (qPCR) in this study.

**Gene**<br>**NCBI**	**Forward (5' -> 3')**	**Reverse (5' -> 3')**
Egr-1<br>NM_007913.5	GTCCTTTTCTGACATCGCTCTGA	CGAGTCGTTTGGCTGGGATA
Fos<br>NM_010234.3	CGAAGGGAACGGAATAAGATG	GCTGCCAAAATAAACTCCAG
Col4a1<br>NM_009931.2	CTGGCACAAAAGGGACGAG	ACGTGGCCGAGAATTTCACC
Nid2<br>NM_008695.2	CACCGAGGACAGTTTCCATT	CCAGTTACCAGGTGCTGGAT
Col11a1<br>NM_007729.3	AGTTGGTCTGCAGTGGCAATTTCG	AGATCCCAGATCCACCGTTTCGTT
Col27a1<br>NM_025685.3	CTCAAGGAGCCGTCAGATCG	AAGATCTCCCCTCCCTGGTC
Col4a2<br>NM_009932.5	GACCGAGTGCGGTTCAAAG	CGCAGGGCACATCCAACTT
Matn2<br>NM_001358780.1	GACGGACGGGCTCAGGAT	GATACCATTGGCCTTGGCTTTA
Comp<br>NM_016685.2	GCGGTTCTATGAGGGTCCTG	GGGAGAAGCAGAAGACACCC
Hdac5<br>NM_001077696.2	CCGGGAACCATCCTTGGAAA	CTTCACCTCCACTGCCACAG

Table S3. List of primers used for ChIP-qPCR in this study.

**Name**	**Forward (5' -> 3')**	**Reverse (5' -> 3')**
P1	CAGCACCTTCCTCGTATTCA	CCTATCTCCGAAGCAACCCA
P2	CAATCTCCTTCCACACAGGCA	TGGGAGGCTCTTGTTTCCAAC
P3	CAGCGGGATCTCTCTGCCAT	AACACTTCCTTCACAGAGCCA
