# Supplementary material for: 4-Phenylbutyric acid activates an NF-κB-Egr-1 axis to control myoblast proliferation and ECM gene expression
Source: iScience. 2026 Sep 24;29(10):117620. doi: 10.1016/j.isci.2026.117620 (PMC13634890; doi:10.1016/j.isci.2026.117620)

Data S1. Uncropped western blot images corresponding to the immunoblotting experiments presented in this study.

Figure 1C

Same membrane reprobed for Myog and Actin

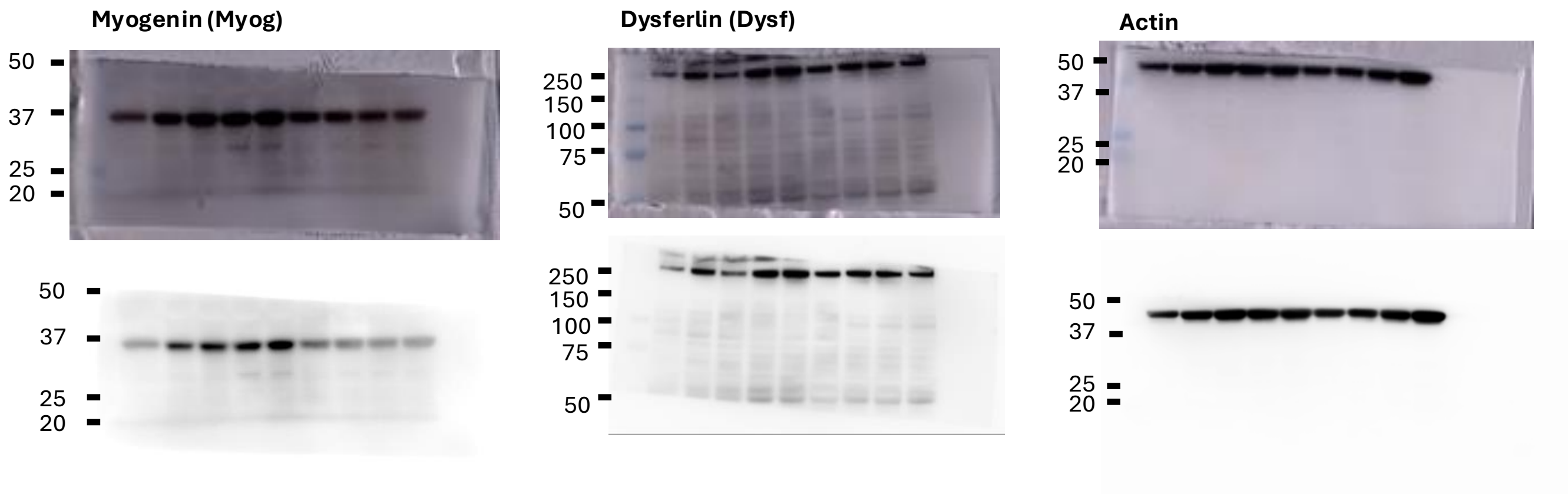

Figure 2E

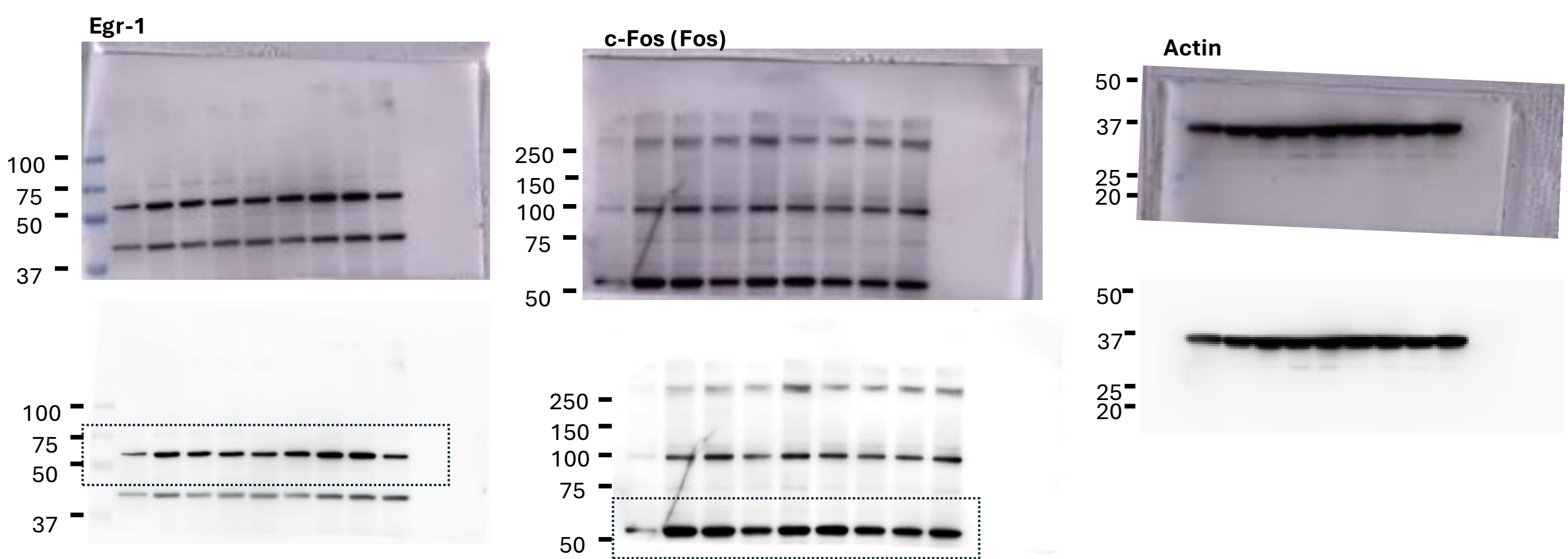

Figure 3A

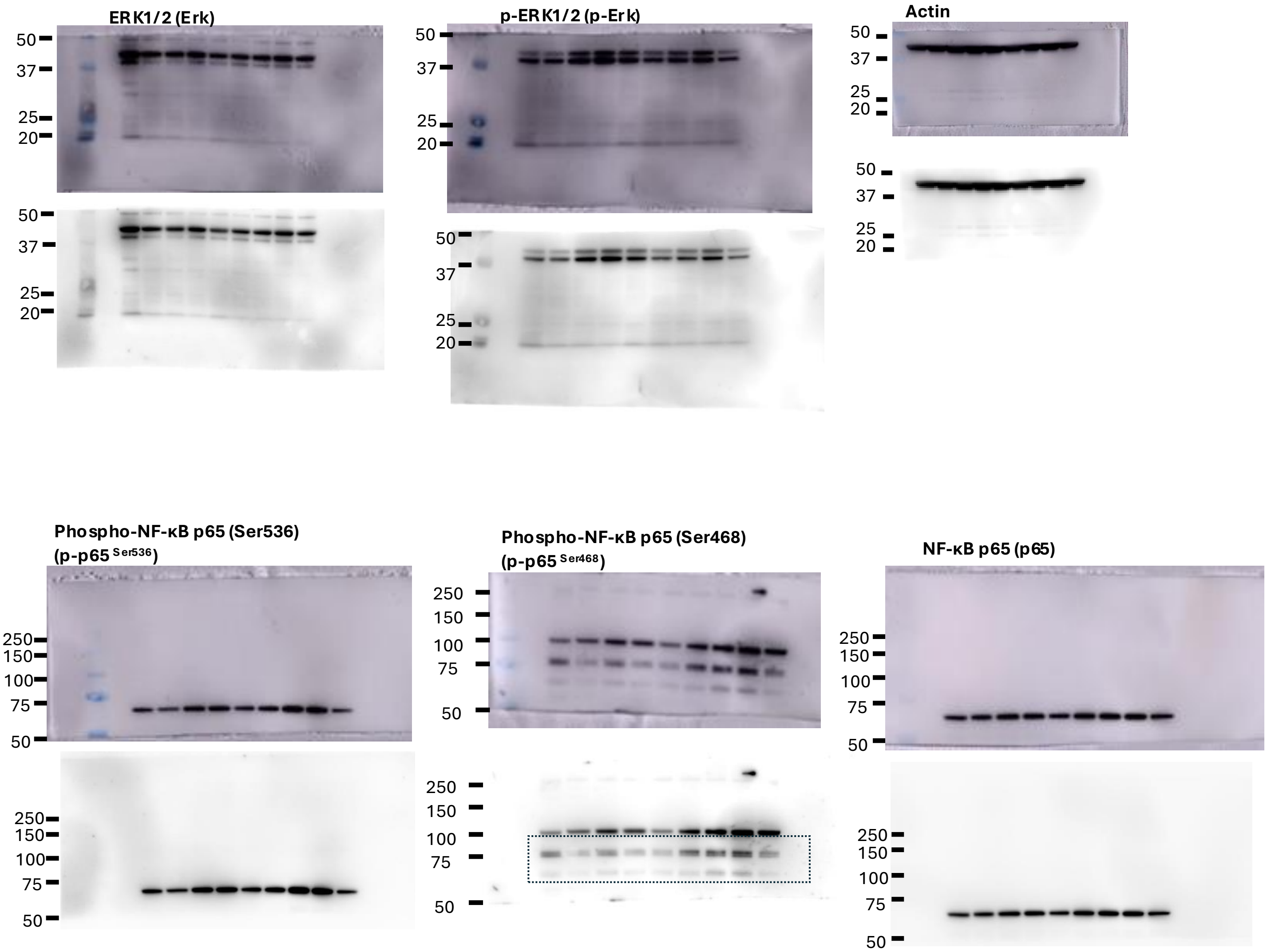
ERK1/2 (Erk)
p-ERK1/2 (p-Erk)
Actin
Phospho-NF-κB p65 (Ser536)
(p-p65 Ser536)
Phospho-NF-κB p65 (Ser468)
(p-p65 Ser468)
NF-κB p65 (p65)
250
150
100
75
50
37
25
20

Figure 3C

Same membrane reprobed for Egr-1 and p-p56Ser536)

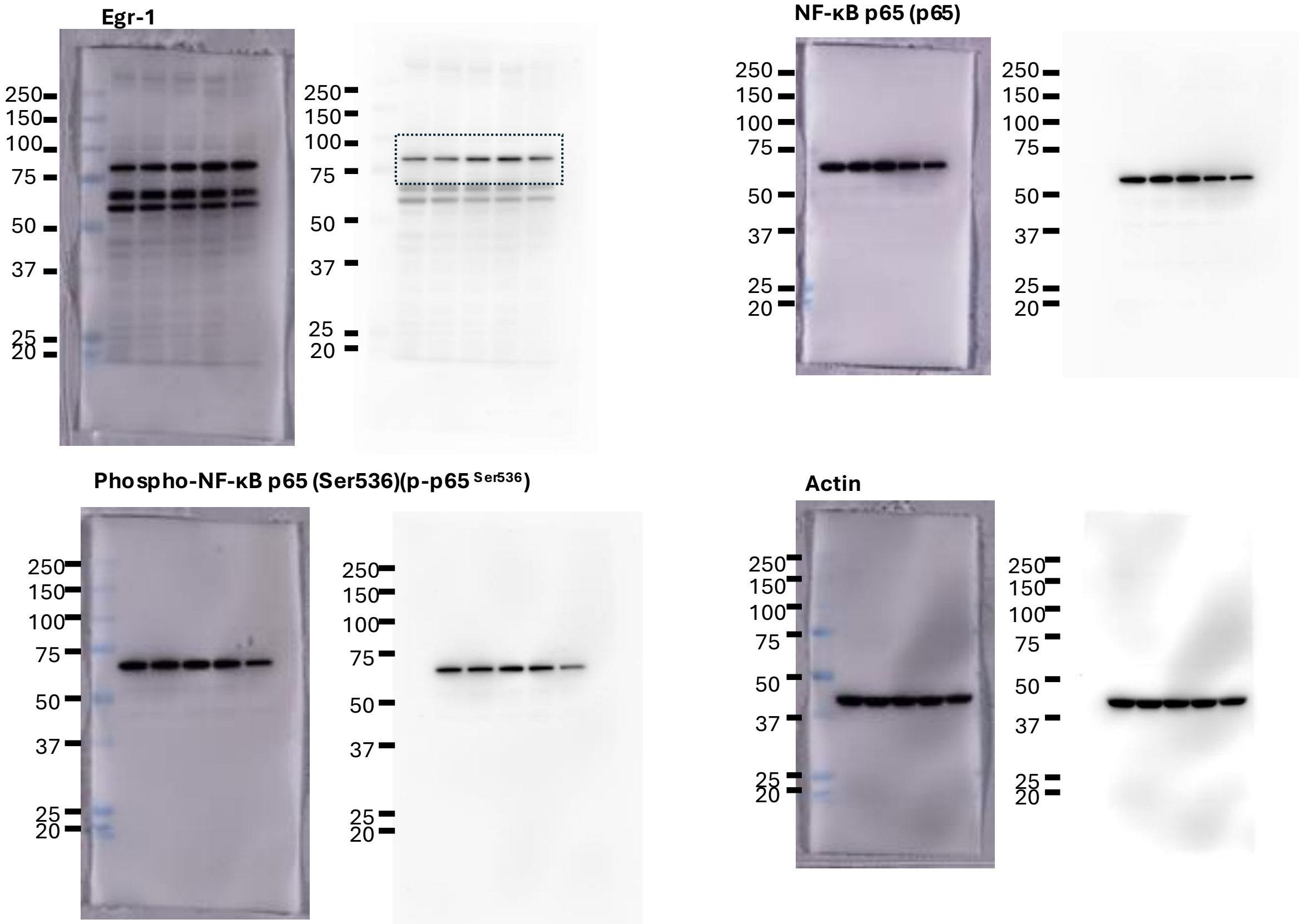

Figure 4A

Same membrane reprobed for H3 and H3K18ac
Same membrane reprobed for H3K9ac and H3K27ac

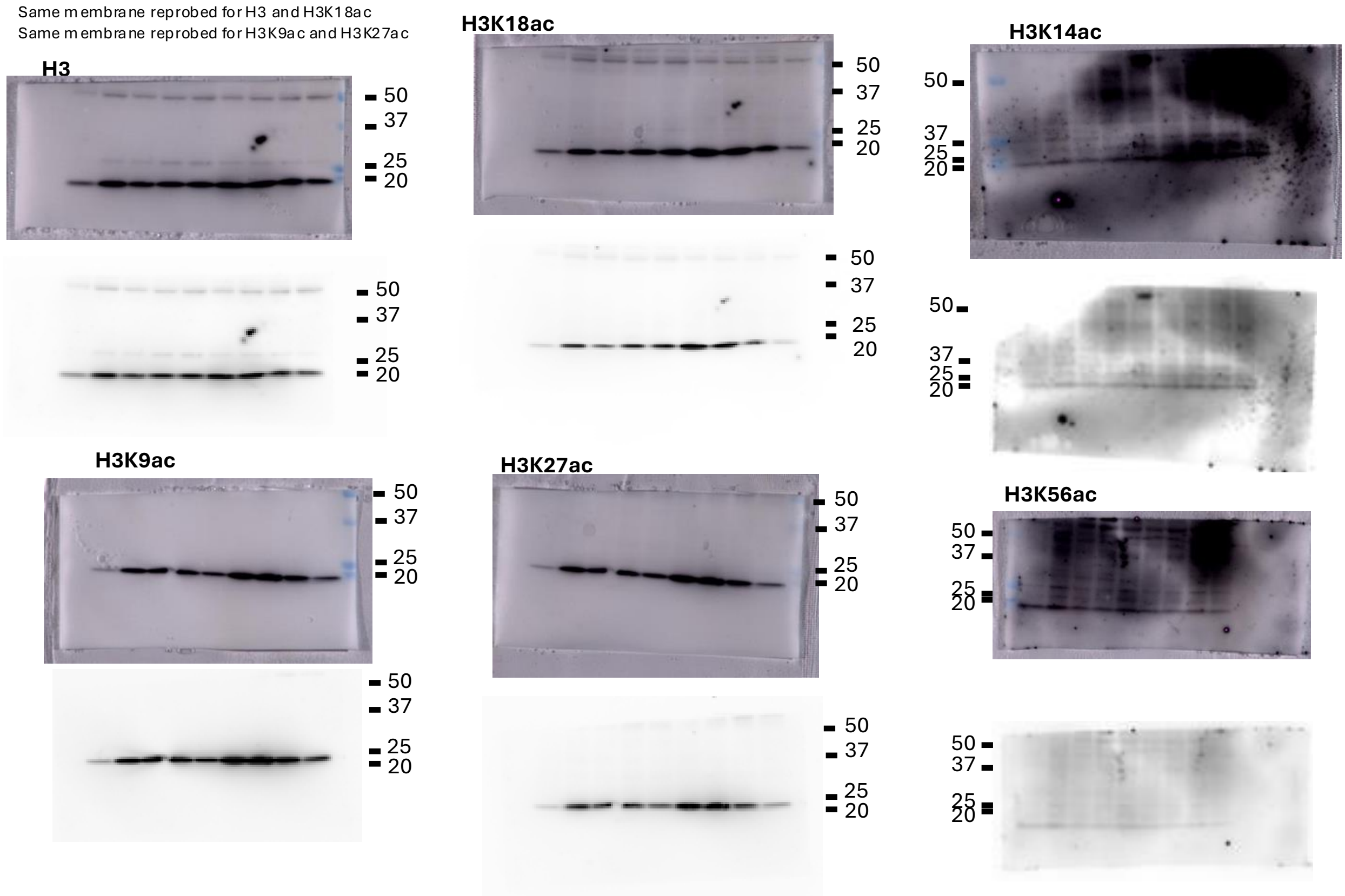

Figure 4C

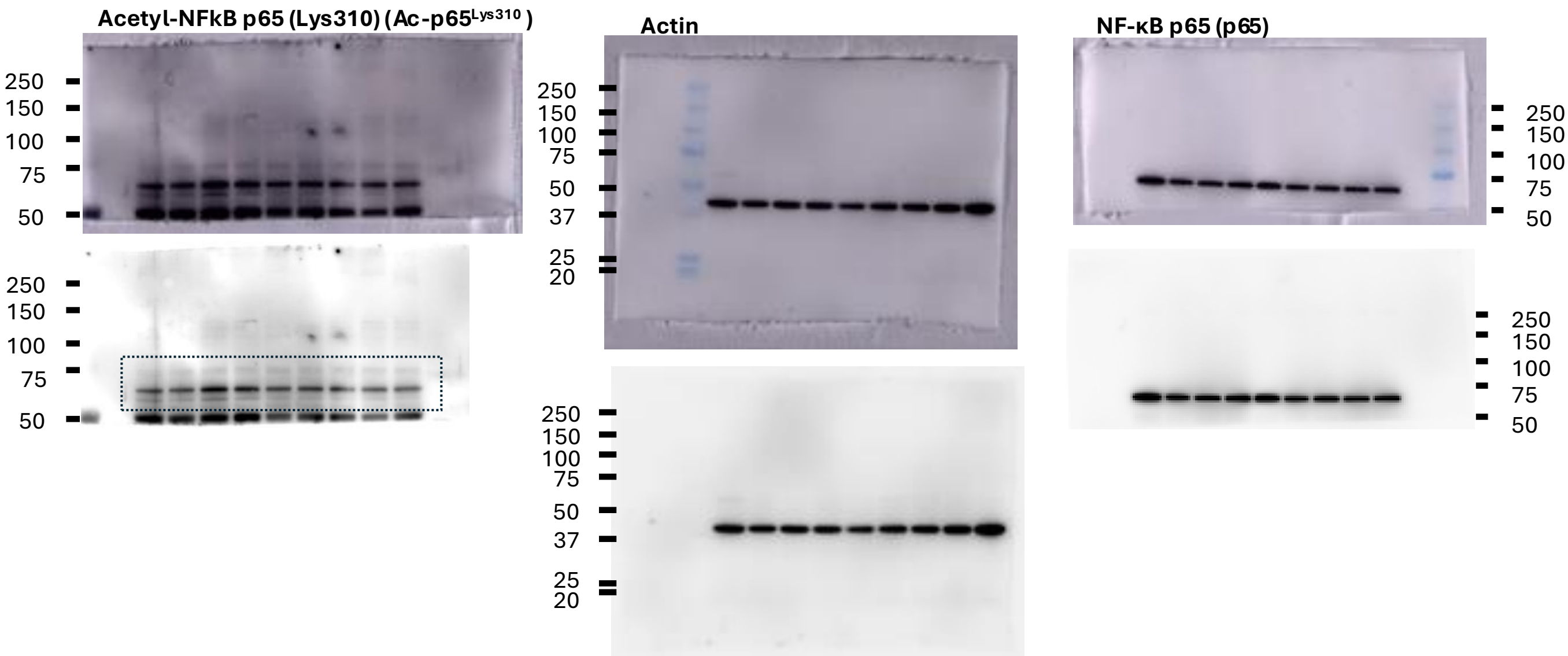

Figure 4E

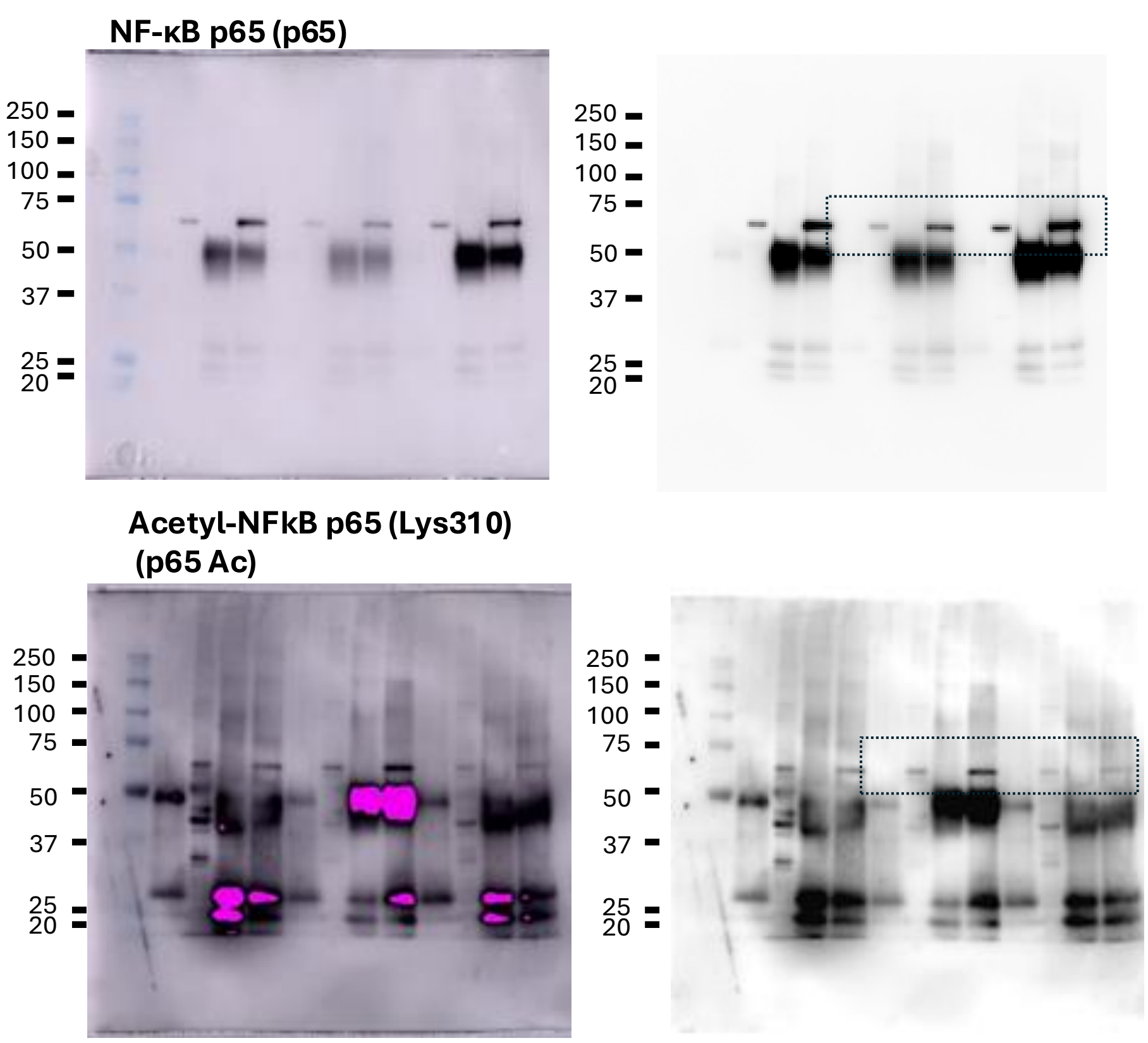

Figure 5B

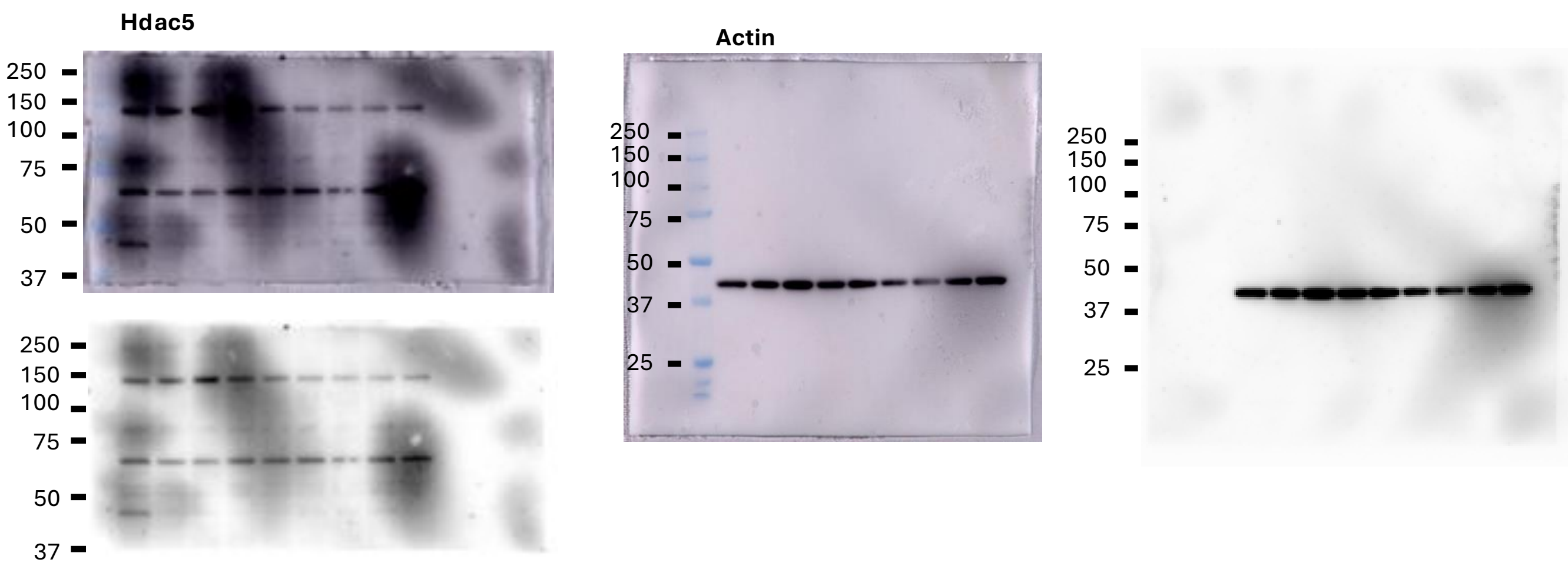

Figure 5E

Same membrane reprobed for Egr-1, p65 and Actin

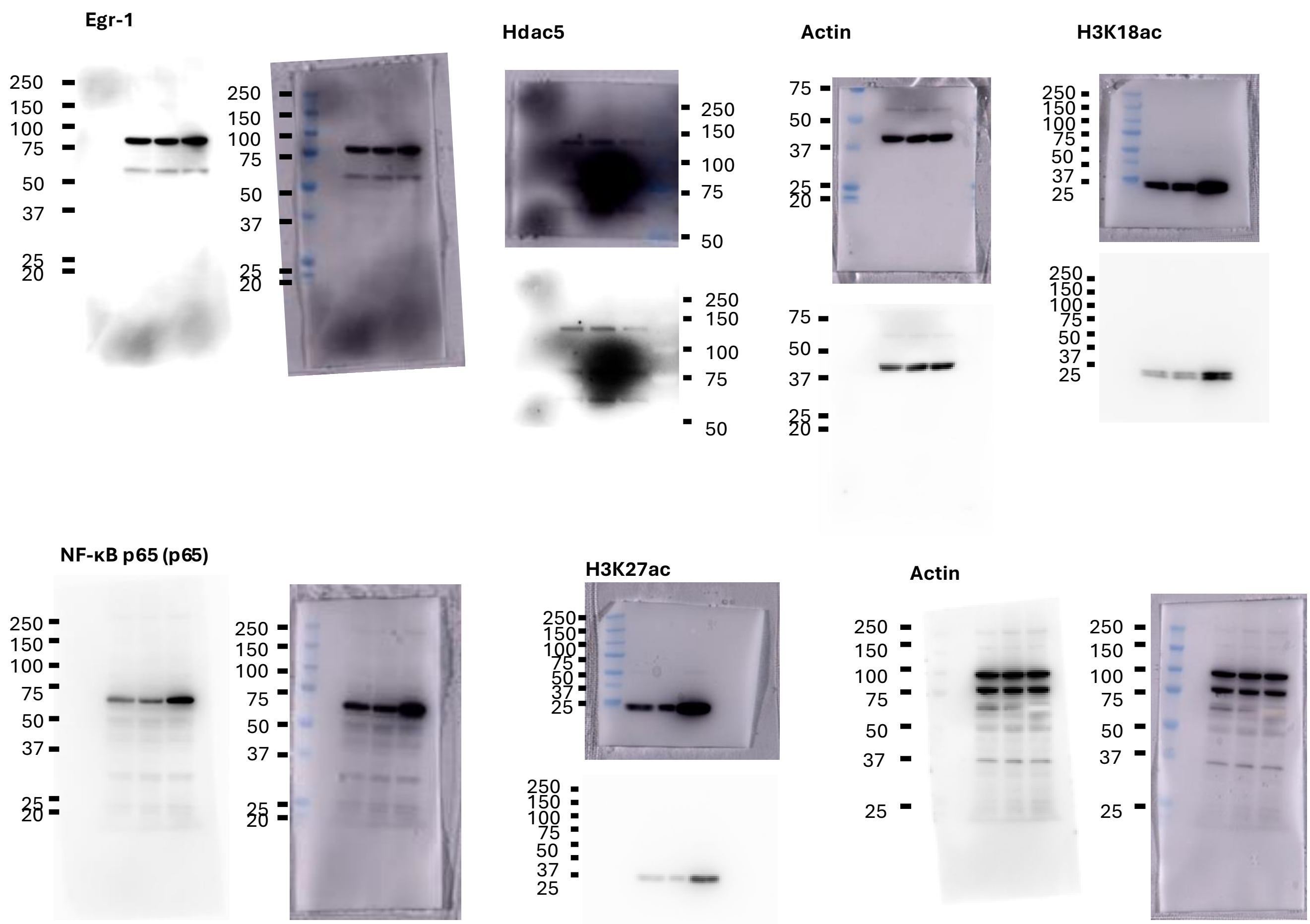

Figure S2B

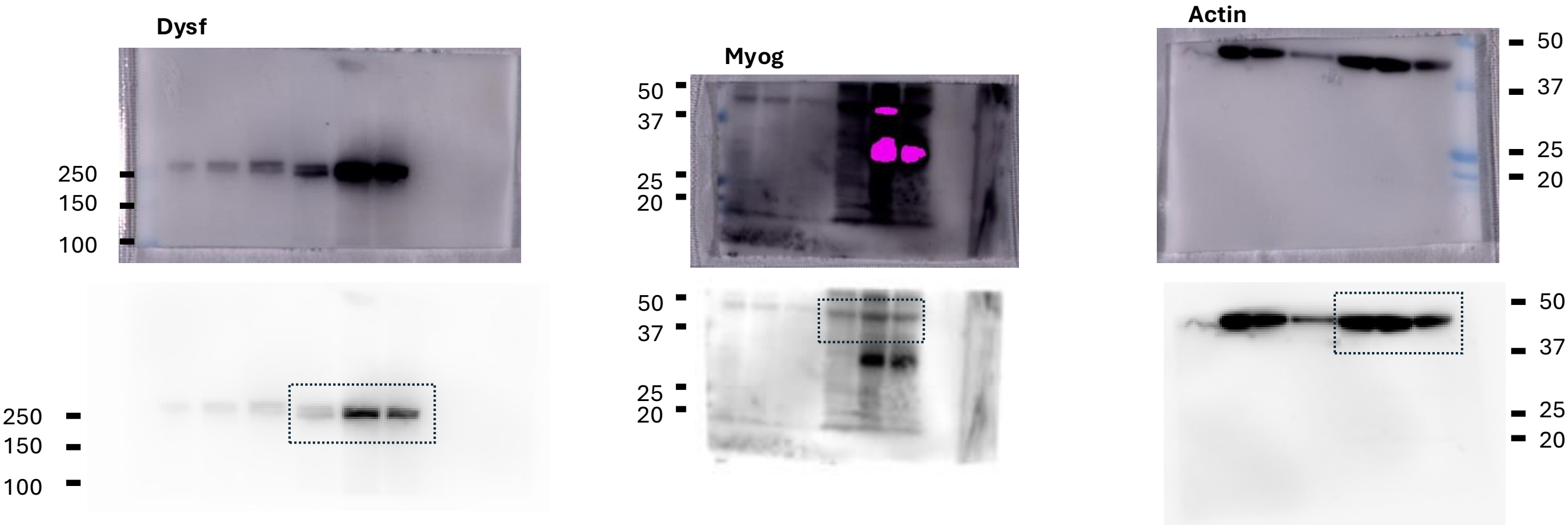

Figure S3C

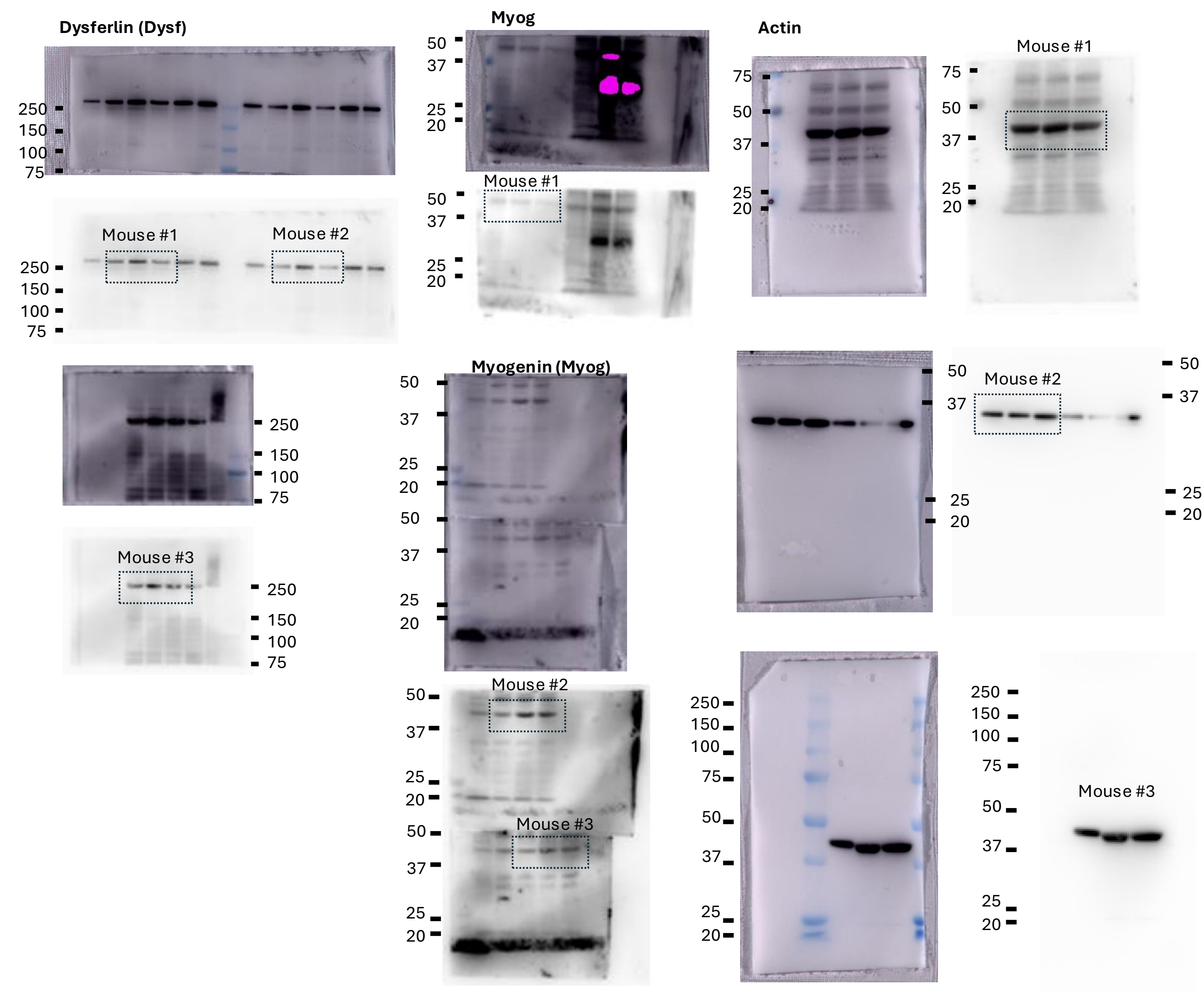

Figure S4A

Same membrane reprobed for Grp78 and IREα
Same membrane reprobed for eIF2α and ATF4

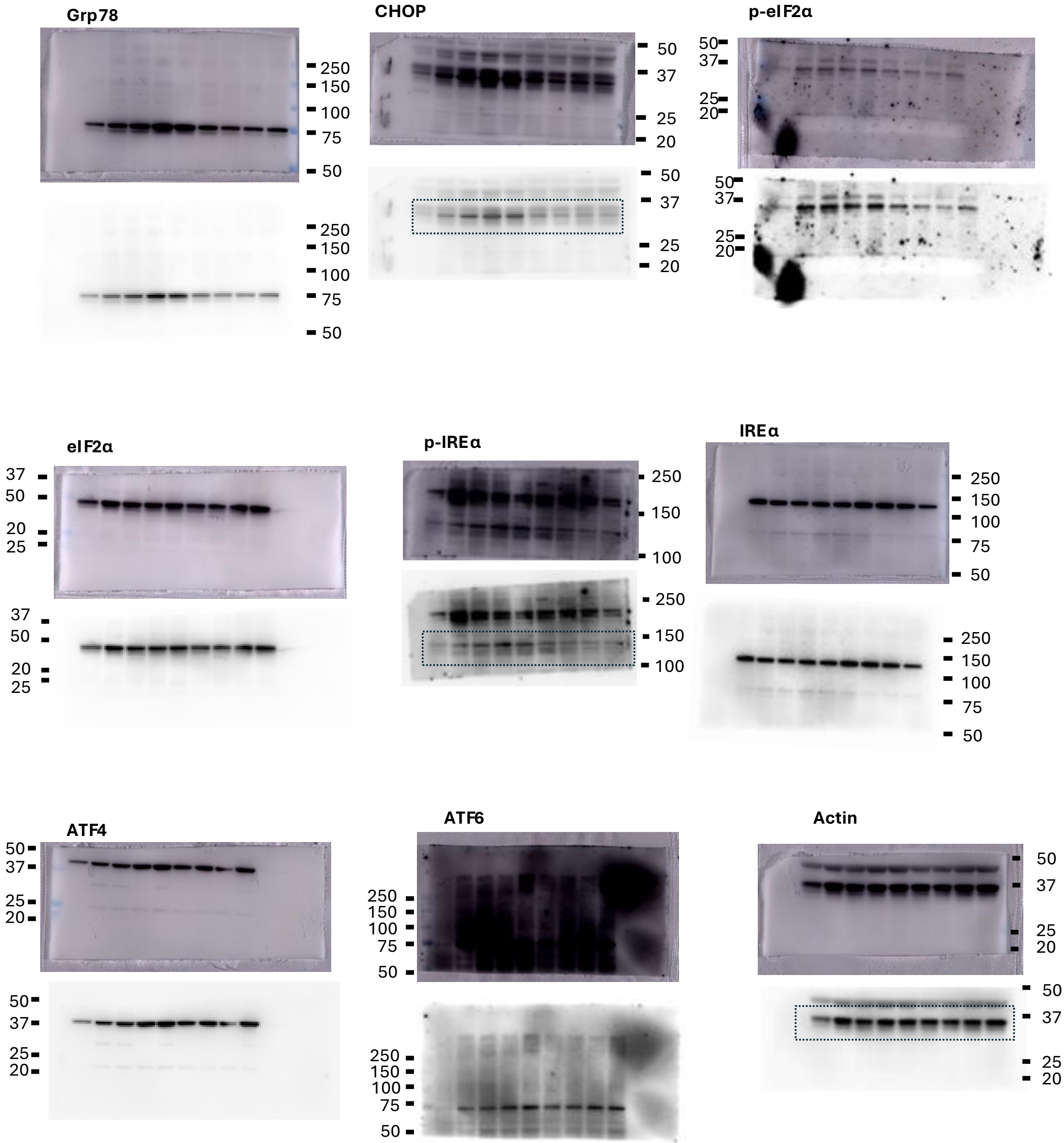

Figure S4C

Same membrane reprobed for CHOP, ATF4 and Actin

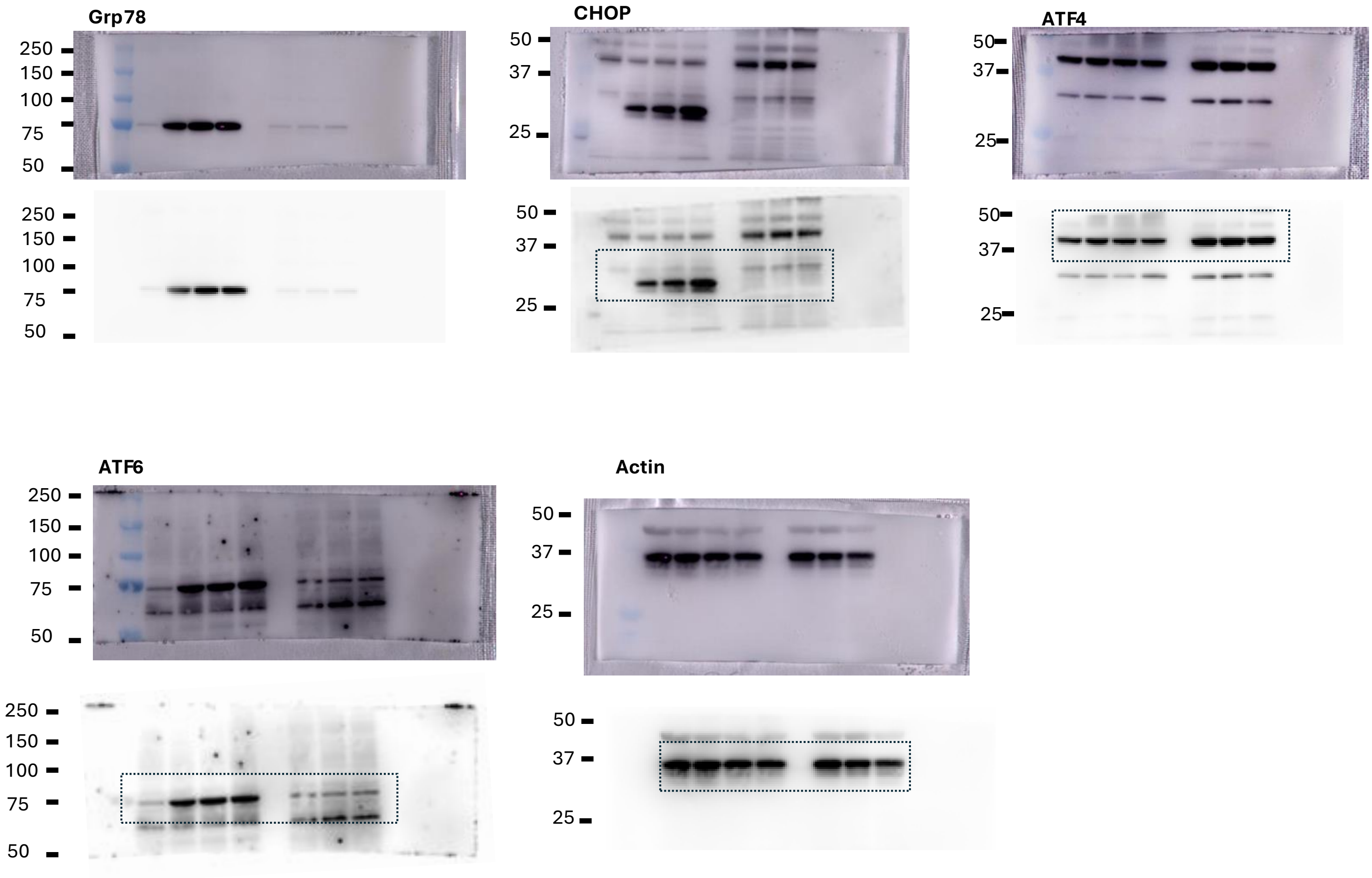

Figure S5A

Same membrane reprobed for Egr-1 and p-65Ser468 (Mouse #2)

Egr-1

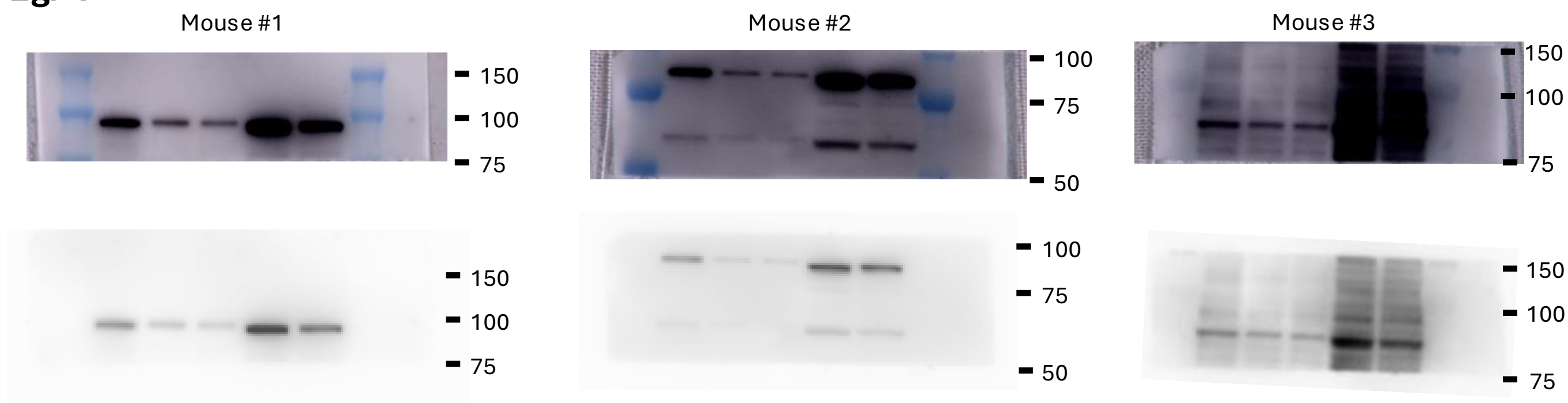

p-p65 ^{Ser468}

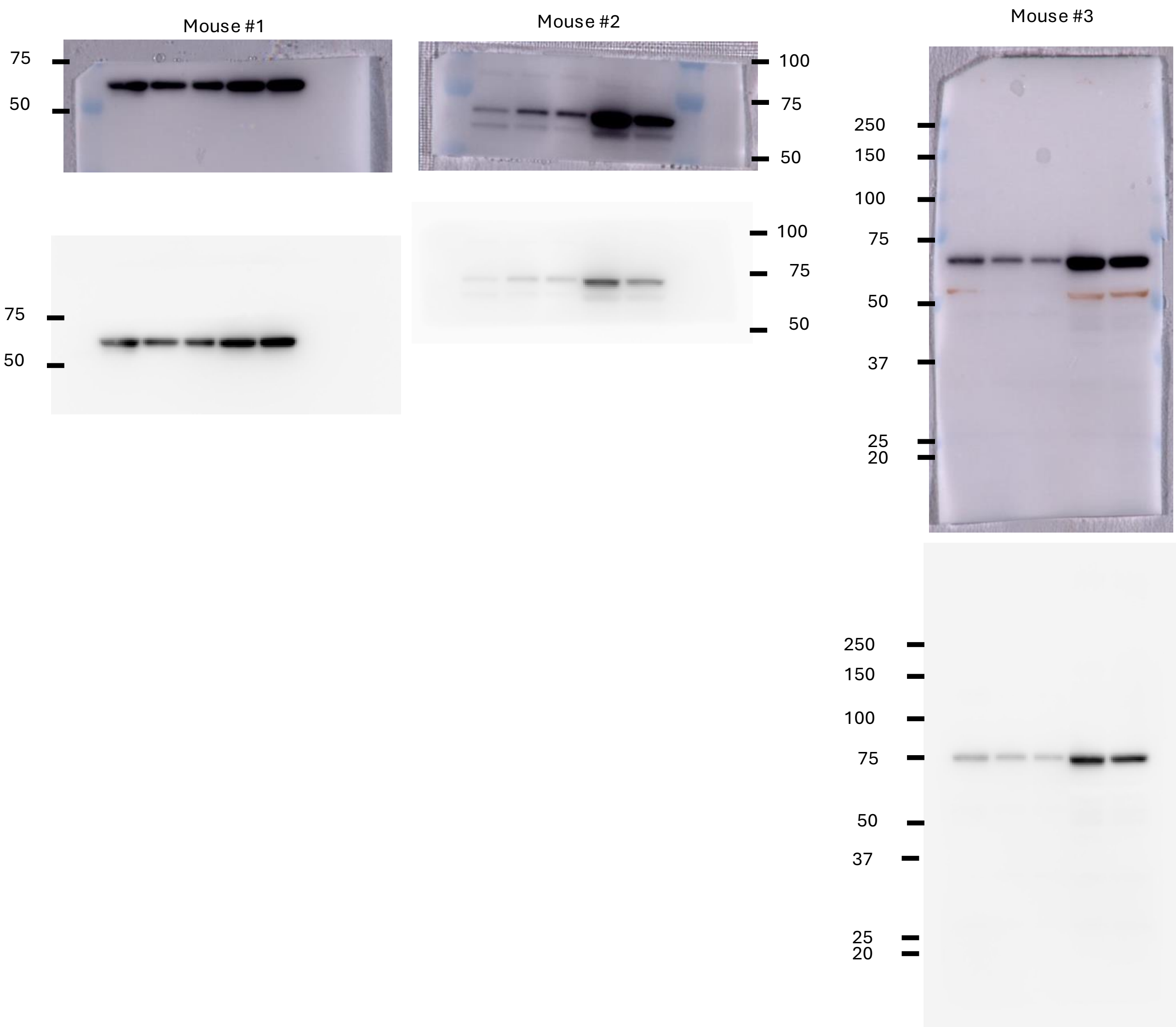

Figure S5A

p65

Mouse #1 Mouse #2 Mouse #3

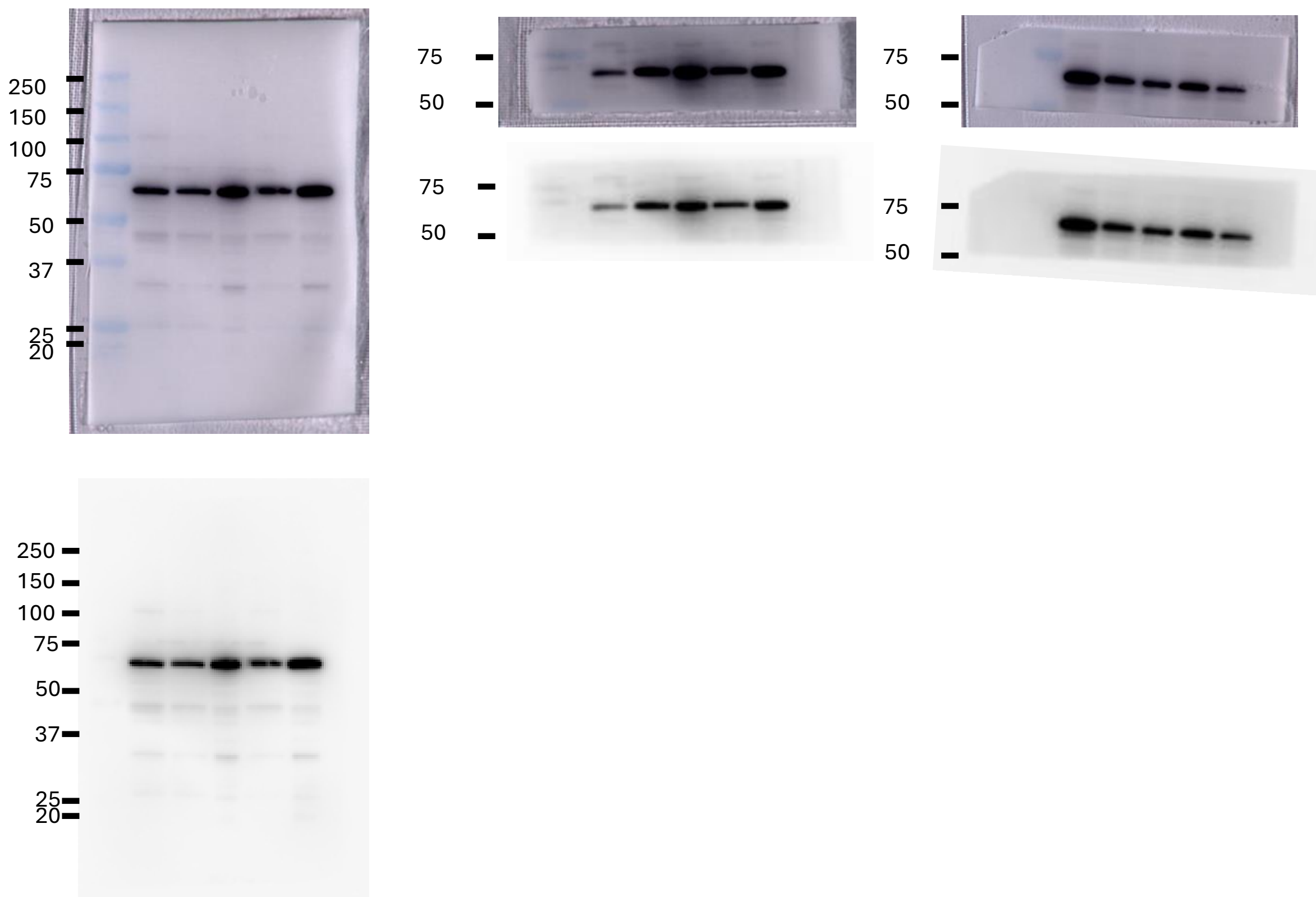

Actin

Mouse #1 Mouse #2 Mouse #3

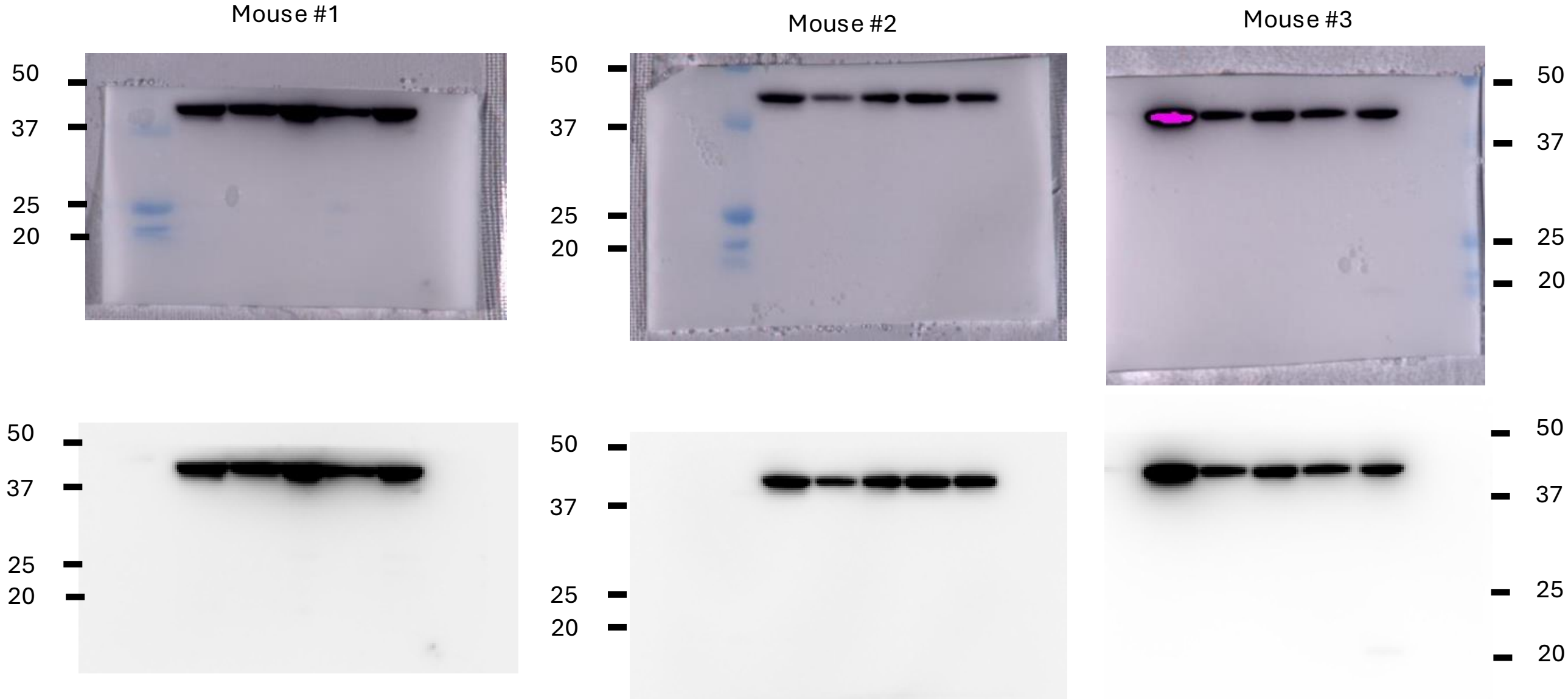

Figure S6A

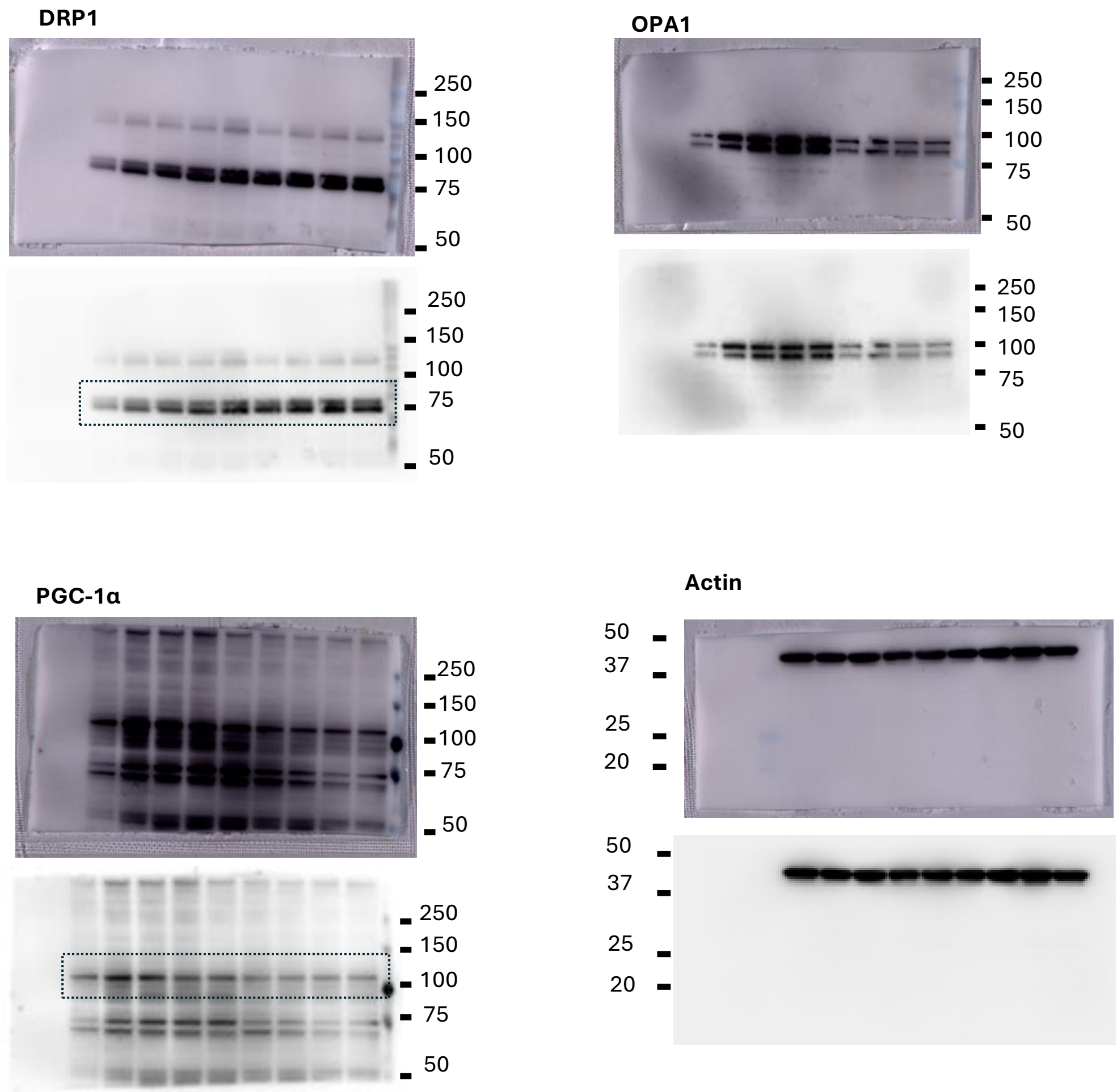
DRP1
250
150
100
75
50
250
150
100
75
50
OPA1
250
150
100
75
50
250
150
100
75
50
PGC-1α
250
150
100
75
50
250
150
100
75
50
Actin
50
37
25
20
50
37
25
20

Figure S8A

Same membrane reprobed for H3K18ac, H3K27ac and Actin (Mouse #1)
Same membrane reprobed for H3K18ac, H3K27ac and p-65Ser468 (Mouse #3)

H3K18ac

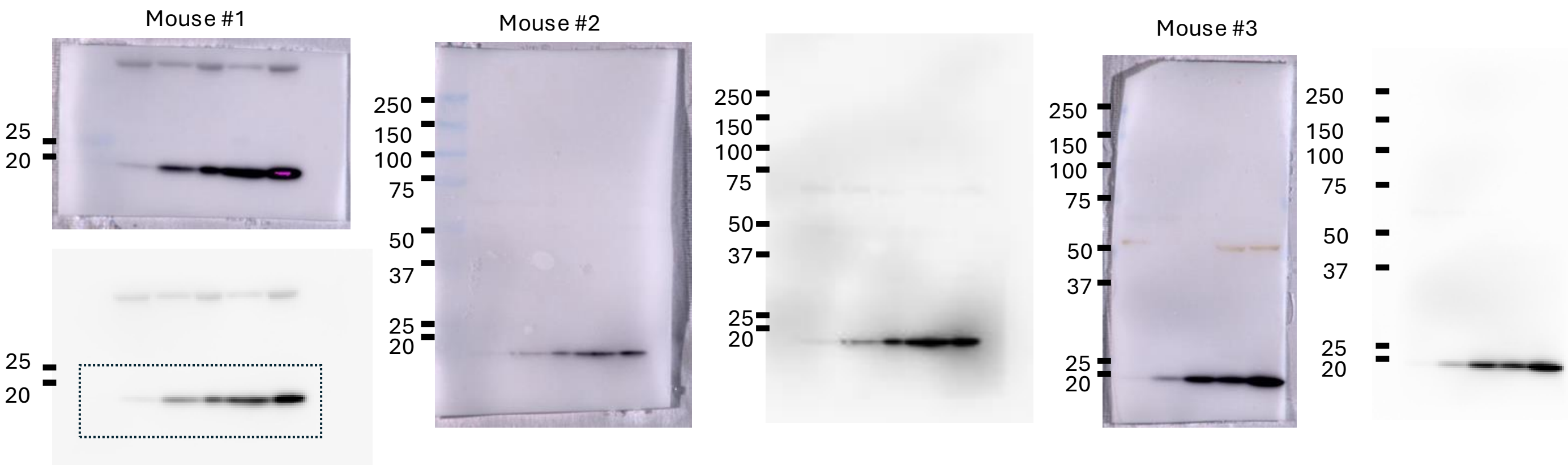

H3K27ac

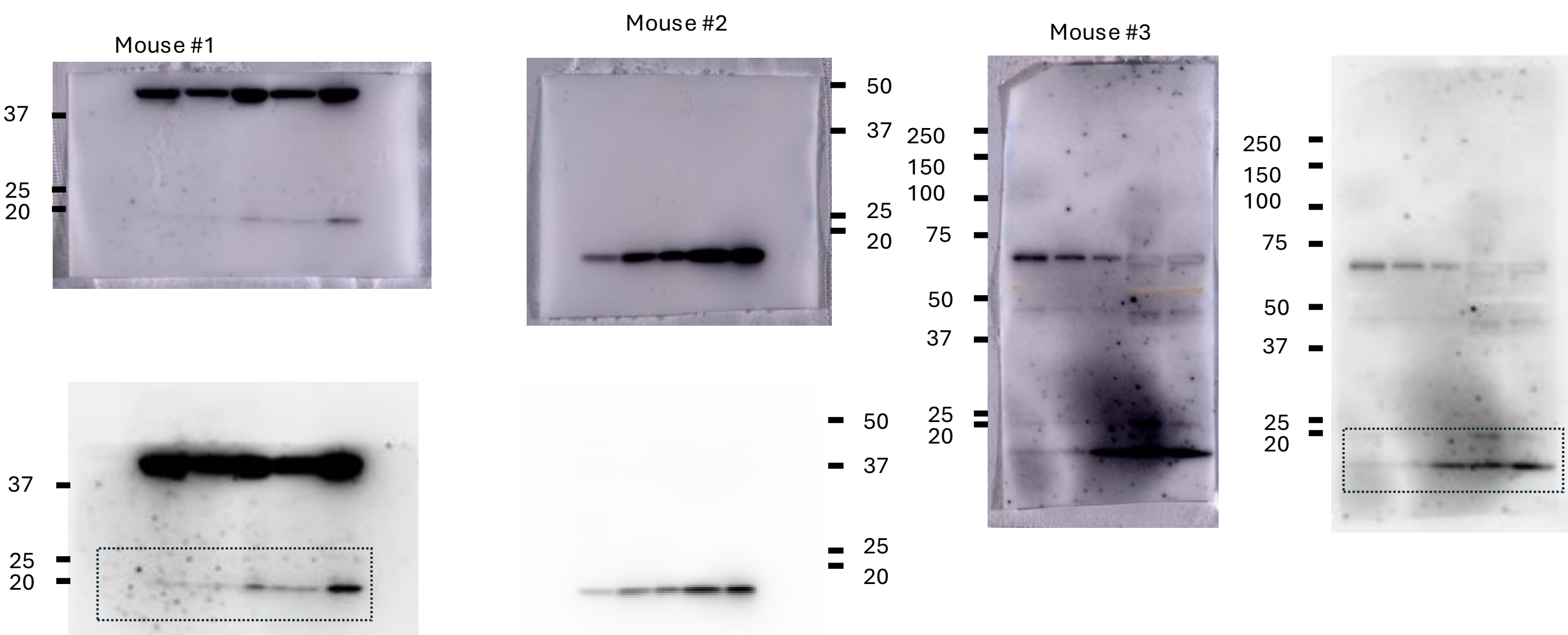

Figure S8A

Hdac5

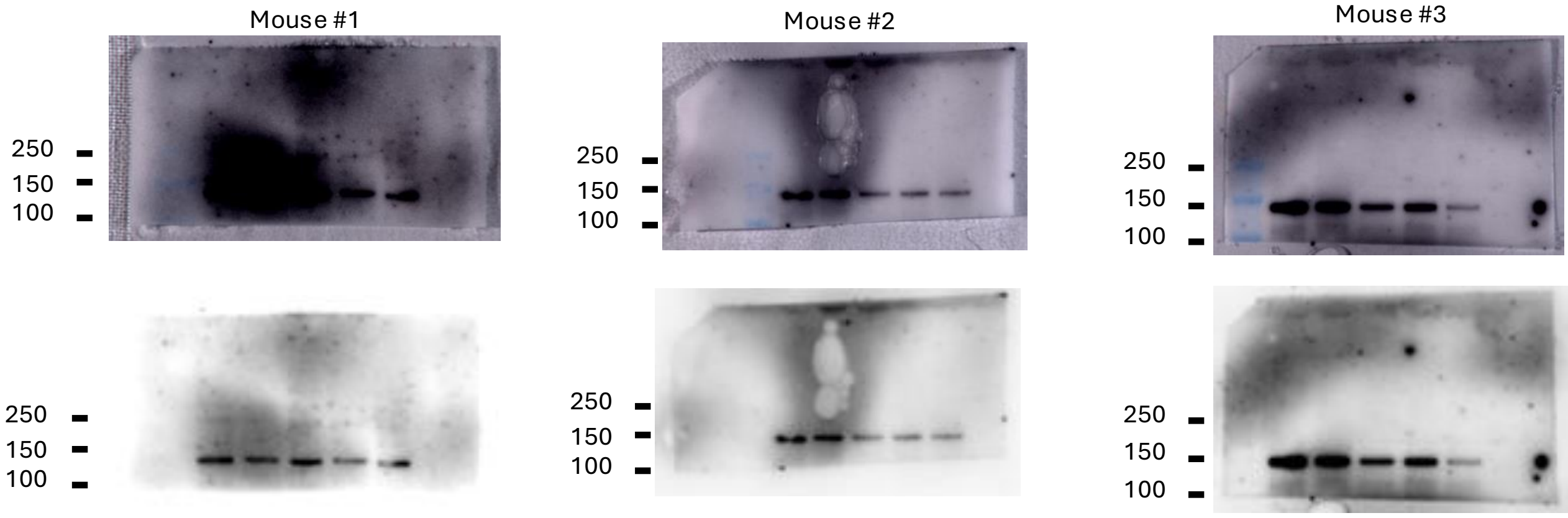

Histone H3

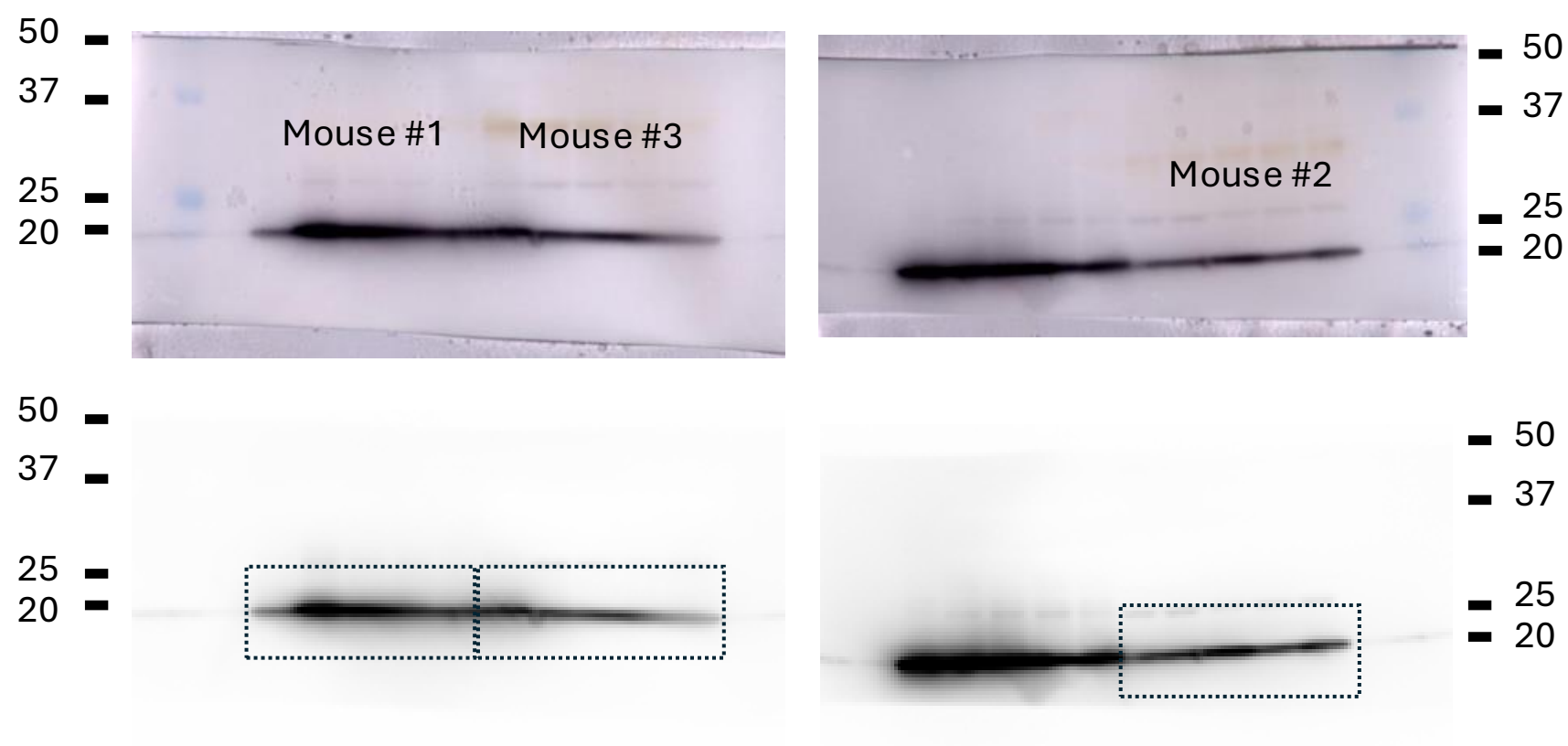

Figure S9A

Hdac5

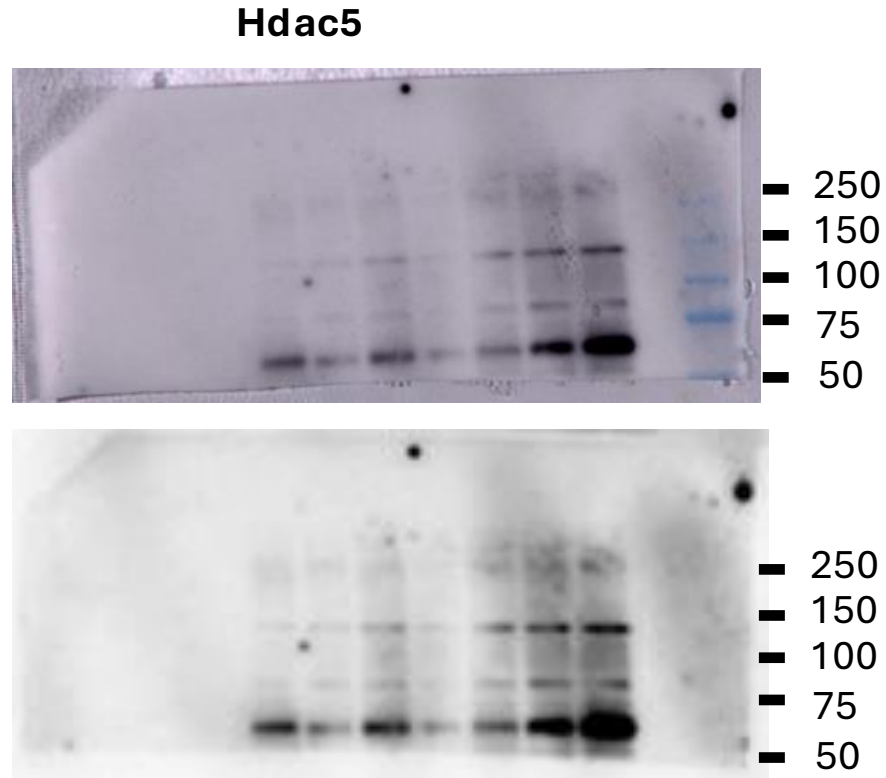

Actin

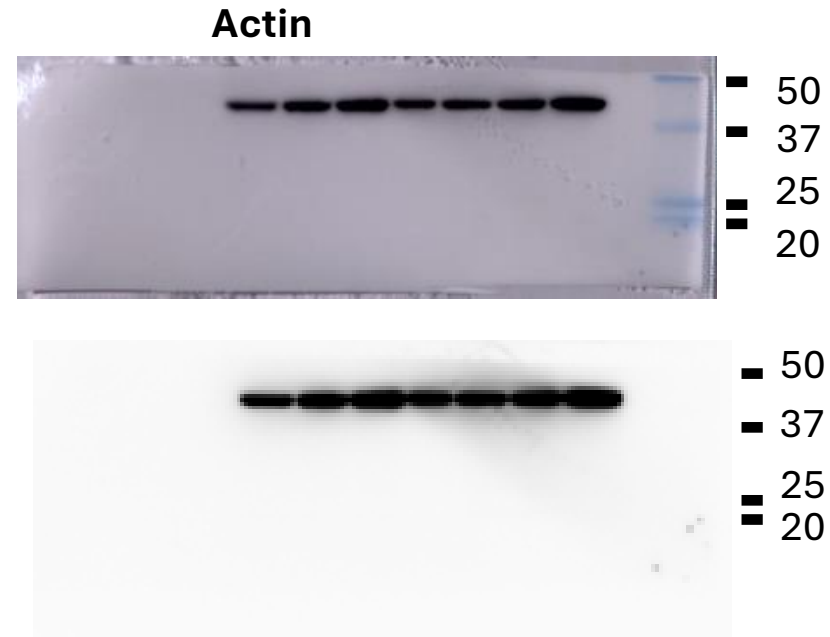

Figure S9B

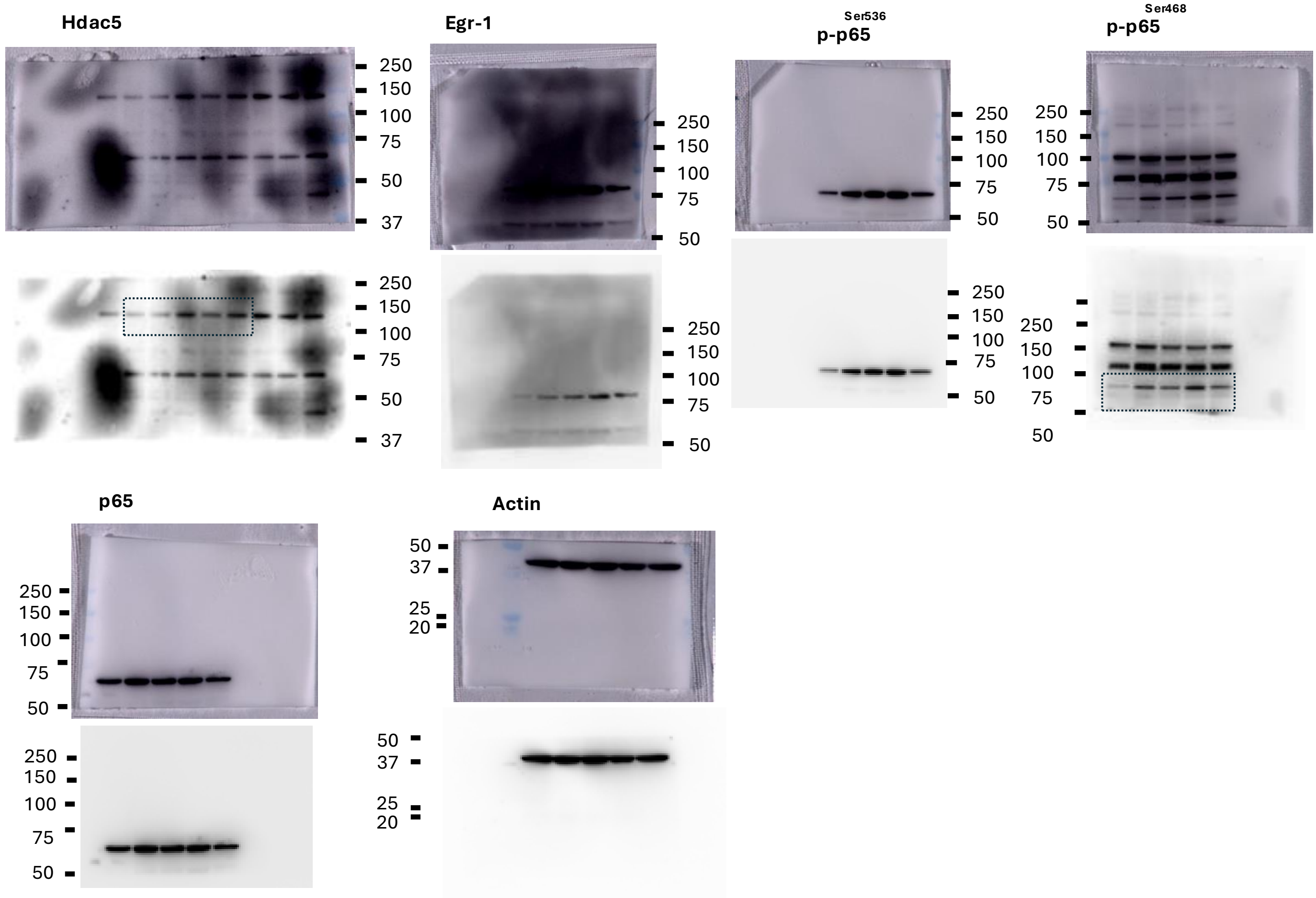

Figure S9D

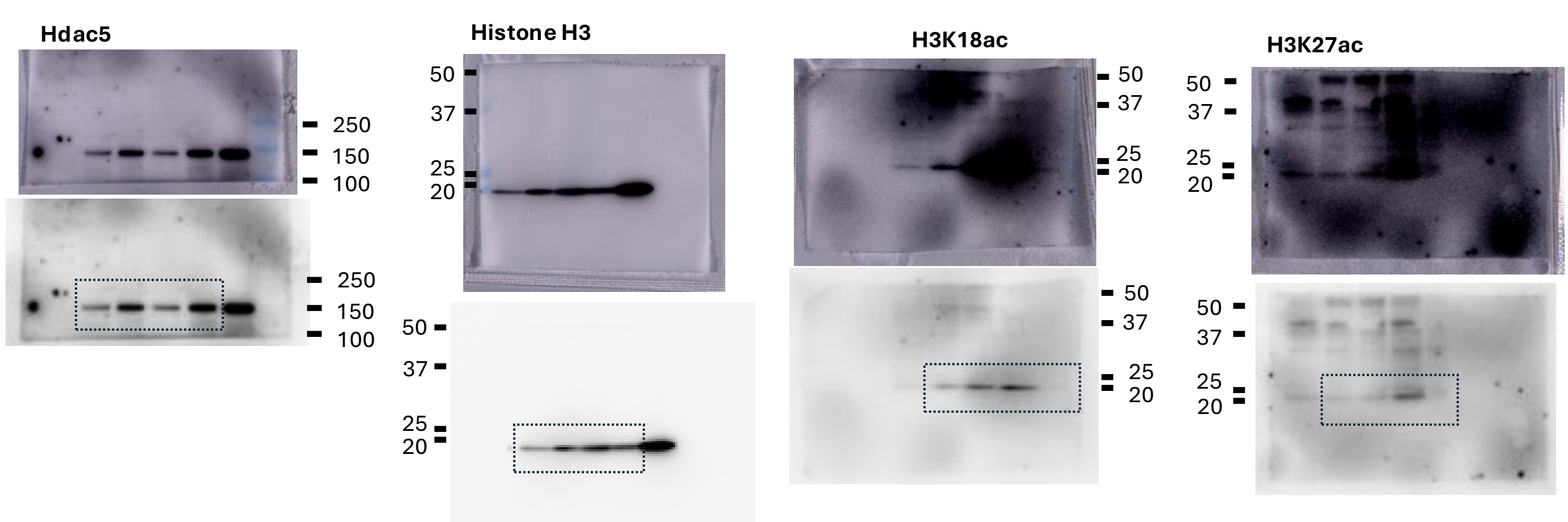

Supplement: Supplementary file 2 [file mmc2.pdf]
